# RNA-seq profiles of chicken type II pneumocyte in response to Escherichia coli infection

**DOI:** 10.1371/journal.pone.0217438

**Published:** 2019-06-05

**Authors:** Lu-Yuan Peng, Zhen-Qiang Cui, Zong-Mei Wu, Ben-Dong Fu, Peng-Fei Yi, Hai-Qing Shen

**Affiliations:** Department of Clinical Veterinary Medicine, College of Veterinary Medicine, Jilin University, Changchun, Jilin, China; Rutgers Biomedical and Health Sciences, UNITED STATES

## Abstract

Avian pathogenic Escherichia coli (APEC) causes great economic loss to the poultry industry worldwide. Chicken type II pneumocytes (CP II cells) secrete surfactants and modulate lung immunity to decrease the infection of the invading pathogen. Nevertheless, the pathogenesis of CP II cells to APEC infection remains poorly understood. Therefore, we conducted global gene expression profiling of CP II cells after APEC-O78 infection to explore the host-pathogen interaction. The differentially expressed genes of CP II cells to APEC infection were characterized by RNA-seq with EB-seq algorithm. In consequence, the mRNA of 18996 genes was identified, and CP II cells responded to APEC infection with marked changes in the expression of 1390 genes. Among them, there are 803 down-regulated mRNAs and 587 up-regulated mRNAs. The KEGG prediction and Gene Ontology terms analysis revealed that the major enriched pathways were related to NF-κB signaling pathway, apoptosis pathway, tight junction, and cytokine-cytokine receptor interaction and other pathways. We adopted qRT-PCR to verify the validity of the selected gene expression. The fold induction of qPCR was similar to the RNA-seq results. These results provide a better understanding of the pathogenesis of APEC, especially apoptosis pathway involved in APEC infection.

## Introduction

Avian pathogenic Escherichia coli (APEC) remains one of the major endemic disease disturbing the global poultry industry [1]. Autologous vaccines only have good efficacy on limited serotype-specific protection, so far diverse serogroups have been linked with disease, particularly O1, O2 and O78 [2]. A recent study showed that the most predominant serogroups were O78 (35.8%) and O2 (14.4%) in eastern China farms [3]. Chickens, turkeys, ducks etc. have currently been shown to be infected with APEC. Furthermore, there are evidences that APEC may lead to extraintestinal infections in humans [4–7].

It is common for poultry pathogens to invade the host through the lung surface and then spread to their target organs [8]. To induce septicemia, APEC must first breach the blood-air barrier in order to enter the bloodstream. The avian lung blood-air barrier consists of the following cells: squamous epithelial cells, endothelial cells and extracellular matrix [9]. Chicken type II pneumocytes (CP II cells), belonging to squamous epithelial cells, serve as a dynamic barrier, which secretes surfactants and modulate lung immunity to decrease the infection of invading pathogens. Human type II alveolar epithelial cell (AEC II cells), similar to CP II cells, could present antigens to CD4^+^ T cells as well [10–15]. When bacteria invade, AEC II cells can secrete antibacterial peptides, cytokines and chemokines, in particular the interleukin (IL)-8, which activate and guide monocytes and macrophage migrate to the site of infection [16]. Based on this, we speculated that CP II cells play a significant role in keeping the host defense. Although studies on APEC-host interactions are mostly focused on immune organs and immune cells, such as spleen, bursa, HD11 macrophages, leukocyte and so on [17–20], the response of CP II cells to APEC infection has never been studied in the genome-wide level.

Although some cytokines and chemokines in chicken to APEC infection has been examined by quantitative real-time PCR (qRT-PCR) [21], RNA-seq technology provides a comprehensive and objective perspective to understand all gene networks[22]. In order to identify differentially expressed genes or gene networks linked to APEC infection, we examined the gene expression changes of CP II cells defending against APEC infection.

## Material and methods

### Bacterial strains

APEC-O78 strain (CVCC1418) was obtained from CVCC (the China Veterinary Culture Collection Center, Beijing, China). DH5α was purchased from Takara Bio Inc (Da Lian, Liaoning, China). APEC-O78 was cultured in Luria-Bertani broth (LB) at 37°C.

### APEC-O78 infection of CP II cells

The culture of CP II cells was performed according to a previous method [23] with modification. In brief, CP II cells were freshly prepared from 13-day-old (Incubation stage) Hy-Line Brown specific pathogen free chicken embryos (Jilin Academy of Agricultural Sciences, Changchun, China)) and kept in DMEM (Hyclone, UT, USA) with 20% sterile fetal bovine serum (FBS) for 18 hours. Before infection, CP II cells were inoculated at a density of 2×10^6^ cells/mL in 6-well plates for 18 hours at 37°C under 5% CO_2_. The cell purity was tested by flow cytometry and TEM (see S1 Fig), showing that CP II cells accounted for 95% of the isolated cells. The original medium was replaced with DMEM. CP II cells were treated with APEC-O78 for 4 h at 37°C under 5% CO_2_ at a multiplicity of infection (MOI) = 100. After 4h, CP II cells were washed with PBS three times and added to 1000 μL Trizol Reagent (TAKARA Biotech, Dalian, China) in cell culture vessel. All assay was conducted in 4 replications.

### Quality control, sequencing library construction and RNA-seq mapping

Total RNA (control and infected cell) was extracted by Trizol. RNA quality was checked using the Bioanalyzer 2200 (Agilent) and stored at -80°C. The RNA quality with RIN (RNA integrity number) > 8.0 is acceptable for cDNA library construction. Bioinformatics data analysis and RNAseq were performed in Shanghai Novelbio Ltd. The sequencing cDNA library of each RNA sample was prepared with the Ion Total RNA-Seq Kit v2.0 following the manufacturer's instruction (Life technologies, USA). In order to remove the reads with lower quality and short sequence, Novelbio adopted the reads filtration to the criteria: all reads with the length >50bp; over 30% base quality >13. In short, the transcript of the poly (A)-containing RNA of chicken was analyzed. The trimmed reads were mapped to chicken genome (NCBI Database) by using Mapsplice program (v2.0). We adopted EB-Seq algorithms to filter the differentially expressed genes annotated by NCBI Database. All differentially expressed genes with the false discovery rate (FDR) under the following threshold set at FDR <0.05 and fold change > 1.5 or < 0.667 were retained for further analysis. RNA-seq data has not been deposited in a public database such as NCBI-GEO.

### Quantitative real-time PCR

Total RNA was used for cDNA synthesis by reversed first strand synthesis kit with oligo (dT) primers following the manufacturer's instruction (Thermo Scientific, Waltham, MA, USA). The PCR reactions were performed on 7500 real-time PCR system (Applied Biosystems, Foster, CA) with FastStart SYBR Green PCR Master Mix (Roche, Mannheim, Germany). PCR was performed as listed below: at 95°C for 3 min for 1 cycle, 45 cycles at 95°C for 15 s, 60°C for 1 min. The chicken ACTB gene served as a housekeeping gene. Primers for real-time PCR have been previously published[21, 24] and are listed in S1 Table. Melting-curve were conducted at the end of amplification to ensure data quality. Each sample was performed in quadruplicate. Fold inductions in target gene expression translated into critical threshold cycle (CT) values were calculated by using the Delta-Delta Ct algorithms.

### Transmission electron microscopy (TEM)

CP II cells were inoculated at a density of 2×10^6^ cells/mL into 6-well plates and treated with APEC-O78 at a MOI of 100. After 4 h infection, cells were washed with PBS three times and fixed in the presence of 2.5% glutaraldehyde for further 2 h. Samples were dehydrated by gradient ethanol solutions 50, 70, 80 and 90%, 10 min each, respectively, then by 100% ethanol 3 times for 10 min each. After that, ethanol was replaced with tert-butyl alcohol, kept for 10 min then overnight at -20°C. Afterwards the cells were embedded in epoxy resin. Samples were cut into the thickness of the 60 nm sections and stained with lead citrate. Sections were observed with a transmission electron microscope (Hitachi-7500, Tokyo, Japan).

### TUNEL assay

CP II cells were grown into 6-well plates for18 h and treated with APEC-O78 and DH5α at a MOI of 100. After 4h of infection, Cells were washed with PBS two times by centrifugation (300 × g) at 4°C, fixed with 70% ethanol for 24 h. The cells were stained using the DeadEnd Fluorometric TUNEL System (Promega, G3250), according to the manufacturer's instructions. The cells were analyzed with a FACSCalibur flow cytometer (BD Biosciences). Measure green fluorescence of fluorescein-12-dUTP at 520 ± 20nm and red fluorescence of propidium iodide at >620nm.

### Gene-Act network

According to the Gene ontology (GO) terms which we selected in APEC infection and control, one gene may interact with several other genes. All gene—gene interactions were pooled together to build the Gene-Act network based on the differential pathways, which helped us to reveal the signaling pathways and key regulatory genes in GC.

### Statistical analysis

The data were presented as means ± SD and evaluated using one-way ANOVA in SPSS 13.0 software (SPSS Inc., Chicago, IL, USA). Differences between groups were analyzed using a one-way ANOVA (Dunnett’s t-test) and a two-tailed Student’s t-test. The results were considered statistically significant at p < 0.05 or p < 0.01.

## Results

### Response of CP II cells to APEC-O78 infection

All samples met the quality standard (see S2 Fig). Mapping statistics were listed in S2 Table. A total of 13.2~15.6 and 12.5~14.3 million sequence reads for control and APEC infection were obtained by RNA-seq. Among the total reads, the mapping rates were about 87.4~89.4% for control group and 88.4~91.5% for APEC infection group. In two groups, 84.1~86.5% and 71.9~86.4% of the sequences were unique mapped rate, respectively. The expression levels of the genes in each sample (see S3A and S3B Table) can be measured by calculating the number of sequence reads (see S4 Table).

**Fig 1:**
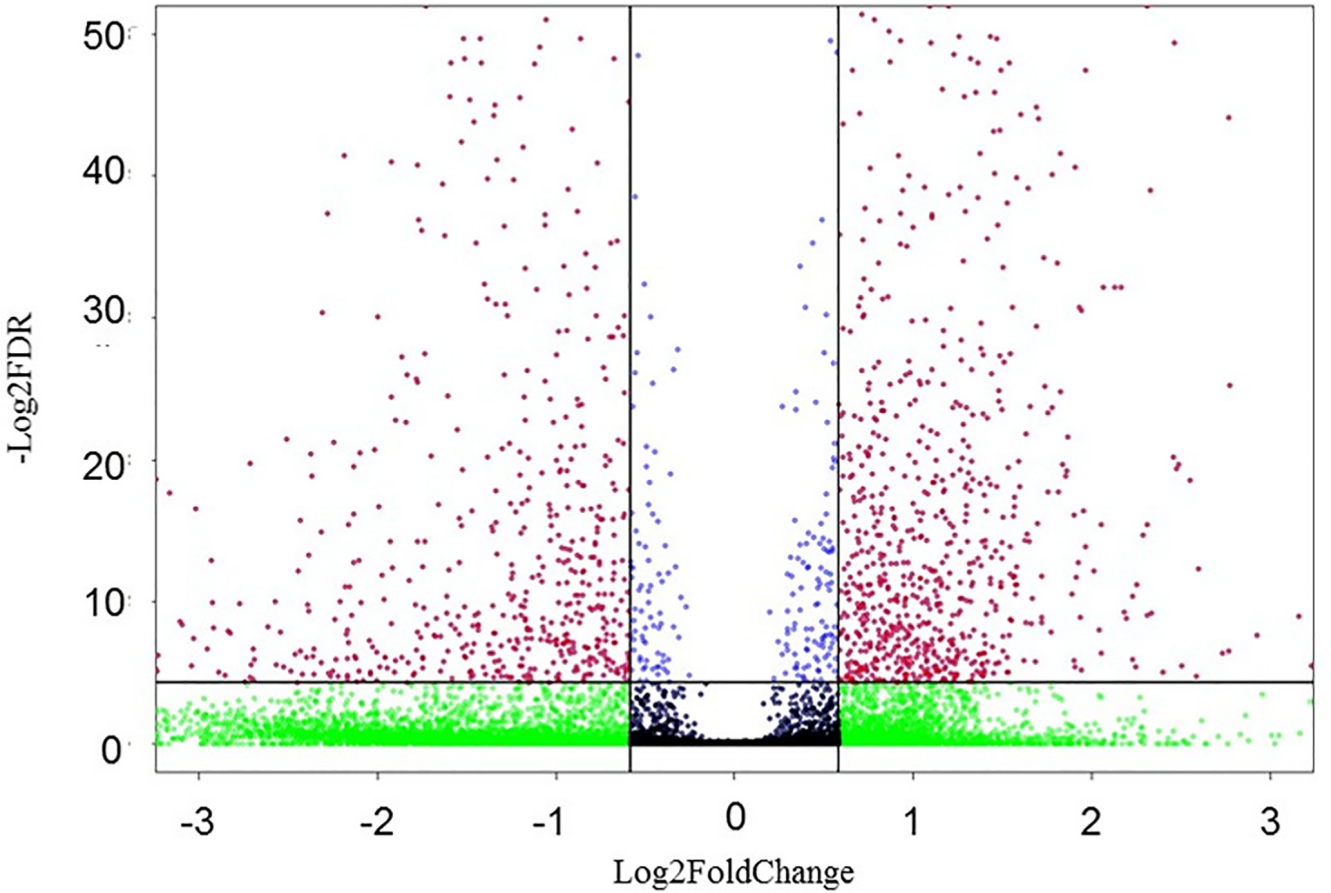
Volcano plot of differentially expressed genes of infected cells (APEC-O78 infection) and non-infected cells (Control). Red dots represent up-regulated genes, green, blue and black dots represent non-differentially expressed genes (Fold Change>1.5 or Fold Change<0.667, FDR<0.05). The horizontal axis represents the fold change between infected and control cells. The vertical axis represents the FDR‐value of the multiple‐test for the differences between samples.

**Fig 2:**
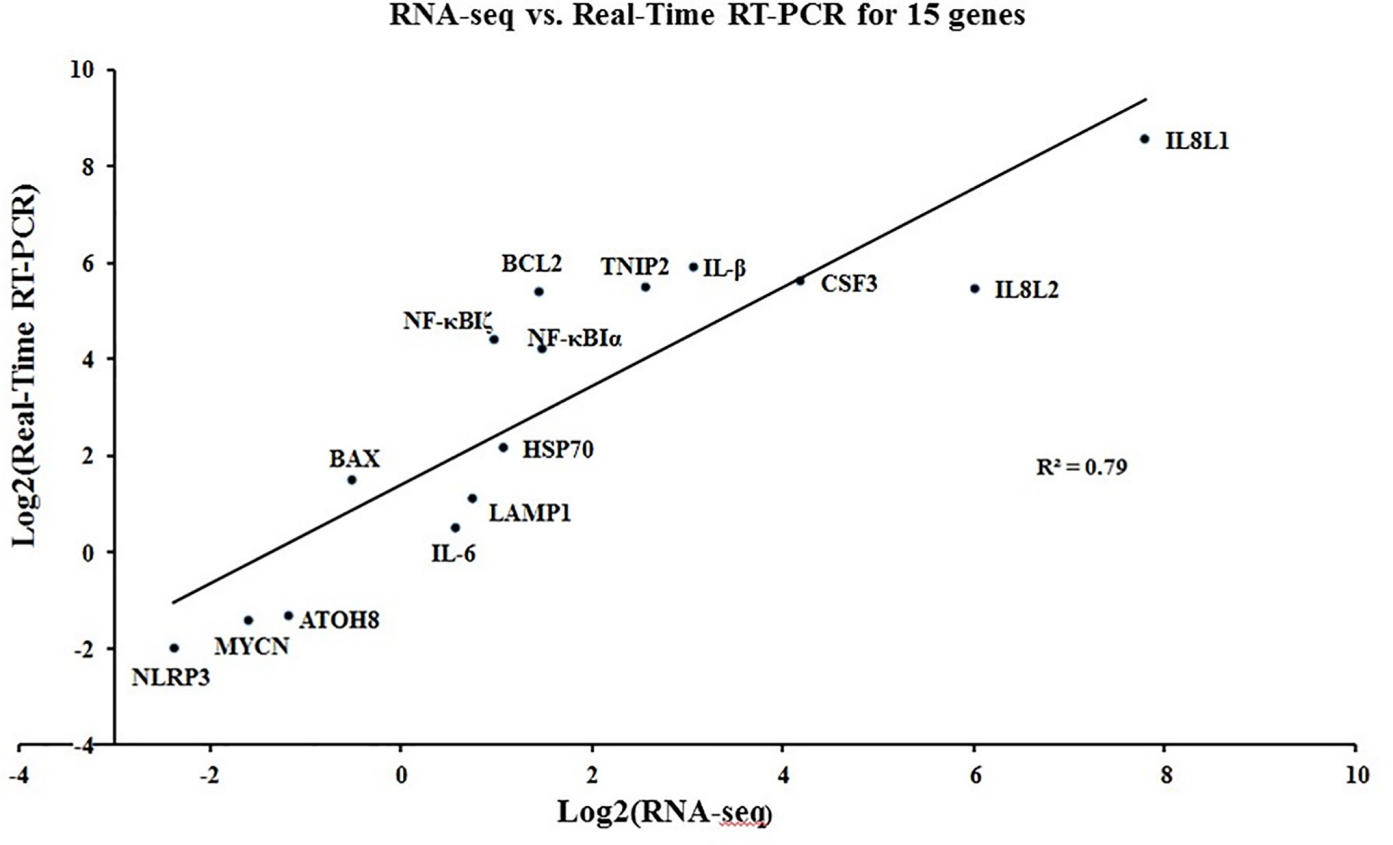
Correlation between RNA-seq and qRT-PCR data on 15 selected genes with various fold changes. Plotted by the logarithm of ratios of means between infected and uninfected samples. Primers are listed in S1 Table.

In consequence, the mRNA of 18996 genes was identified, and CP II cells responded to APEC-O78 infection with marked changes in the expression of 1390 genes (Fig 1). Among them, there are 587 down-regulated mRNAs and 803 up-regulated mRNAs (see S3 Fig). The reliability of the RNA-seq data were validated by qRT-PCR of 15 genes with varied fold changes in expression, in which no conflicts were observed between the qRT-PCR and RNA-seq datasets (Fig 2). The two datasets had a good correlation (R^2^ = 0.7977).

### GO analysis

**Fig 3:**
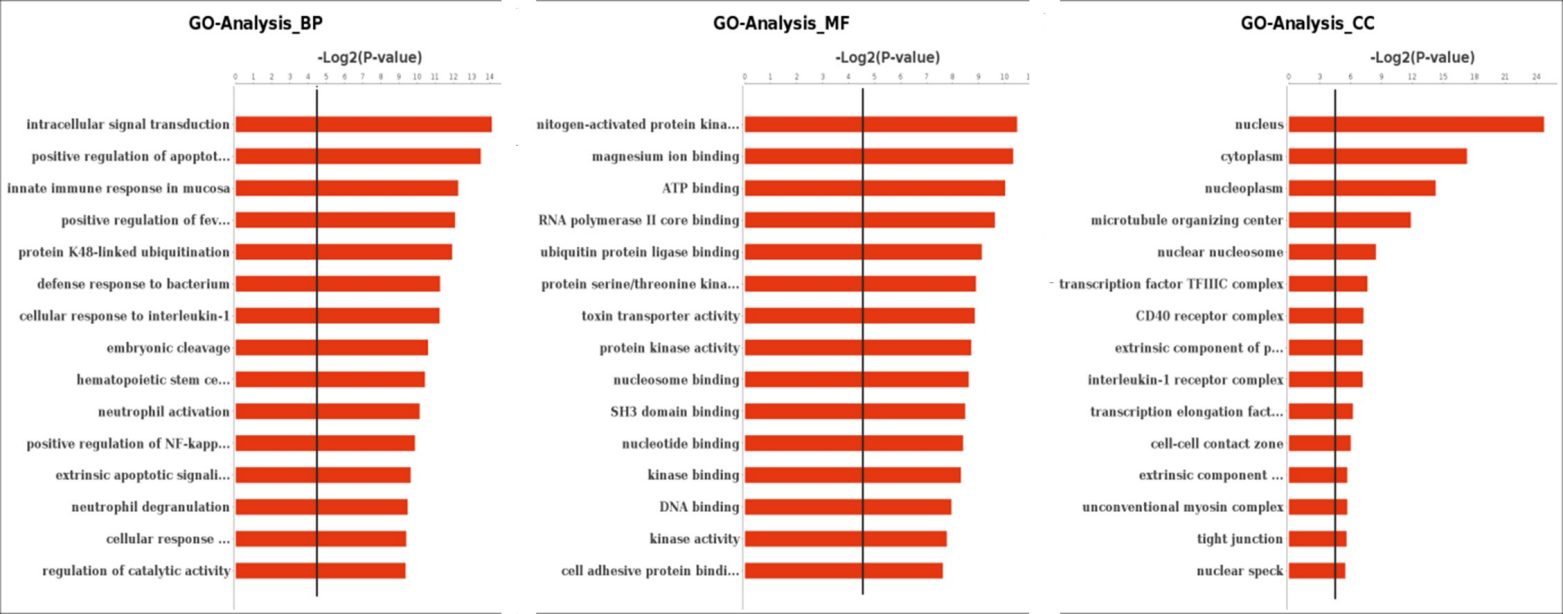
Gene ontology (GO) analysis of regulated genes in chicken type II pneumocyte in response to APEC-O78 infection. GO terms in biological process, molecular function and cellular components. The black line stands for “p = 0.05”.

GO analysis is a universal normative classification system of gene function and involved in three areas: namely biological process, molecular function, and cellular component. We focused on GO terms with a corrected P value < 0.05 and FDR < 0.05. When the infected cell was compared to control, 3567 GO biological processes had annotations for 977 genes, including 346 down-regulated genes and 641 up-regulated genes. Moreover, 521 GO cellular components were annotated for 999 genes, including 636 up-regulated genes and 363 down-regulated genes. For 1044 GO molecular function annotated for 966 genes, of which 626 genes were up-regulated while 340 genes were down-regulated (Fig 3, S3C–S3E Table).

The major category groups in the biological process were intracellular signal transduction, positive regulation of the apoptotic process, innate immune response in mucosa, defense response to bacterium and others. Besides, the major clusters of cellular components were focused on nucleus, cytoplasm, nucleoplasm, microtubule organizing center. In addition, the major clusters of molecular functions were magnesium ion binding, ATP binding, RNA polymerase II core binding and others.

### Pathway analysis

**Fig 4:**
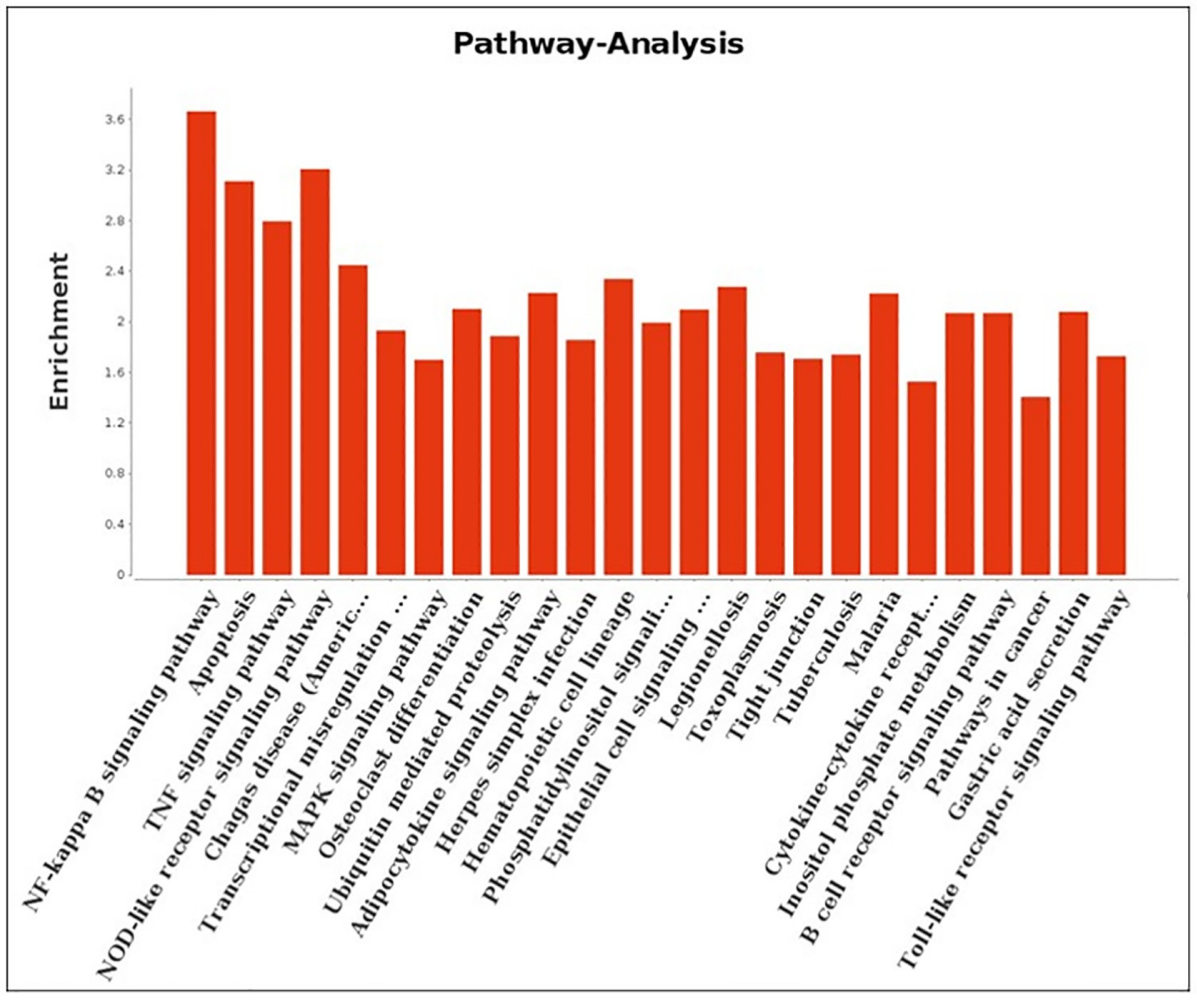
Pathway analysis of differentially expressed genes according to the KEGG database. Top-ranking regulated pathways identified by KEGG according to P-value <0.01 and FDR <0.01.

In order to further determine the gene function of differentially expressed genes in CP II cells after APEC-O78 infection, we mapped them using the KEGG Genomes database for the analysis of signaling pathways. The results revealed that the differentially expressed genes were involved in the NF-κB signaling pathway, apoptosis pathway, tight junction, and cytokine-cytokine receptor interaction and other pathways related to host defense responses against APEC-O78 infection (Fig 4, S3F Table).

### Gene-Act network

**Fig 5:**
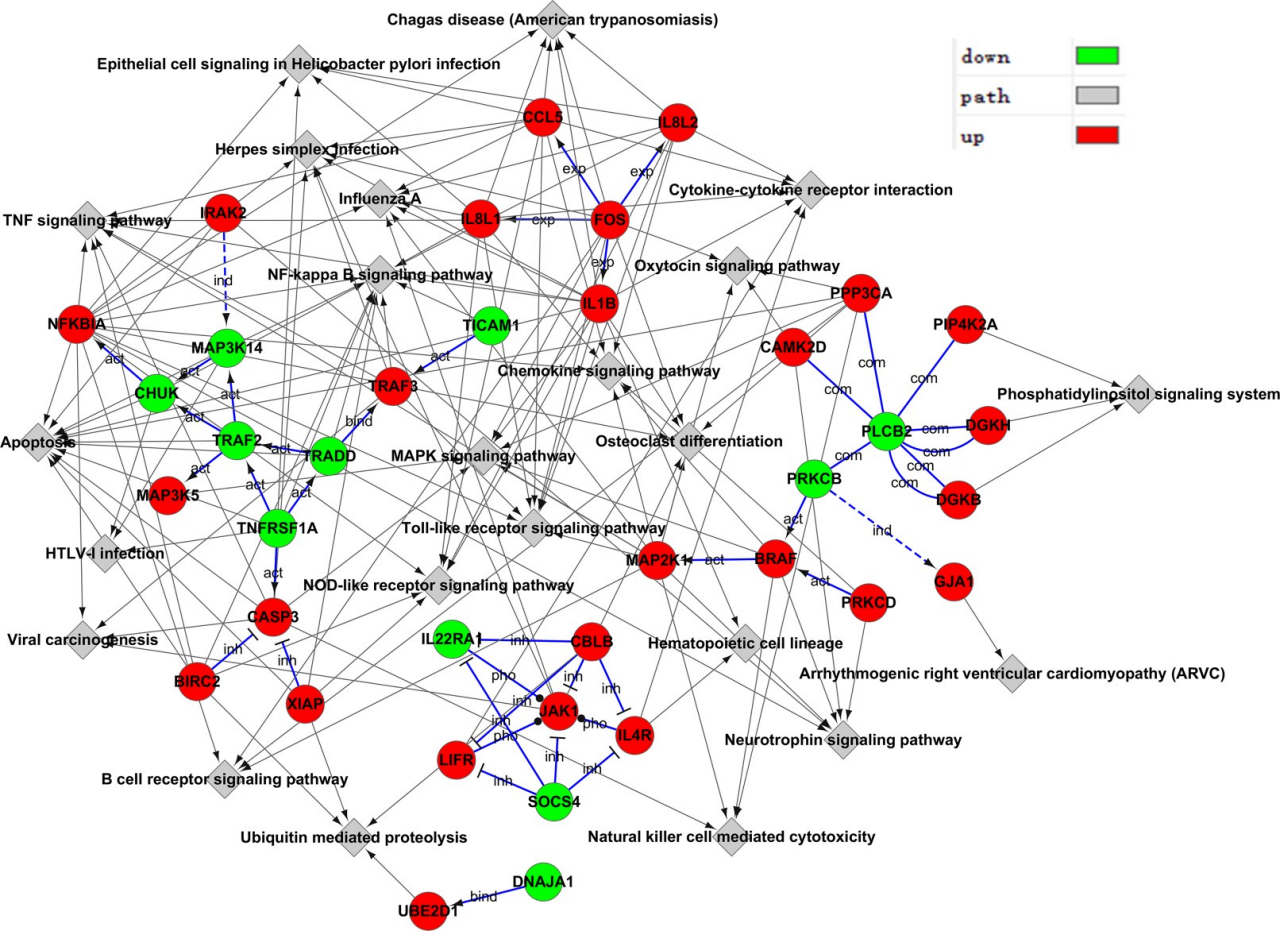
Gene-Act network of differential genes according to pathways in the database. Red dots represent up-regulated genes and green dots represent down-regulated genes. The arrows indicate the connection and regulatory relationship between two genes. Genes that have more connections with other genes have a higher degree score.

Based on GO categories, one gene may interact with several other genes. We pooled the differential genes and built a network of the interactions of differentially expressed genes. A high degree protein regulates or is regulated by many other proteins, which implies an important role in the Gene-Act network. The genes with high degree were known as key genes in the interaction network including PLCB2, TRAF2, JAK1, CBLB, FOS, SOCS4, TNFRSF1A and TRADD (Fig 5, S3G Table).

### TEM and TUNEL

**Fig 6:**
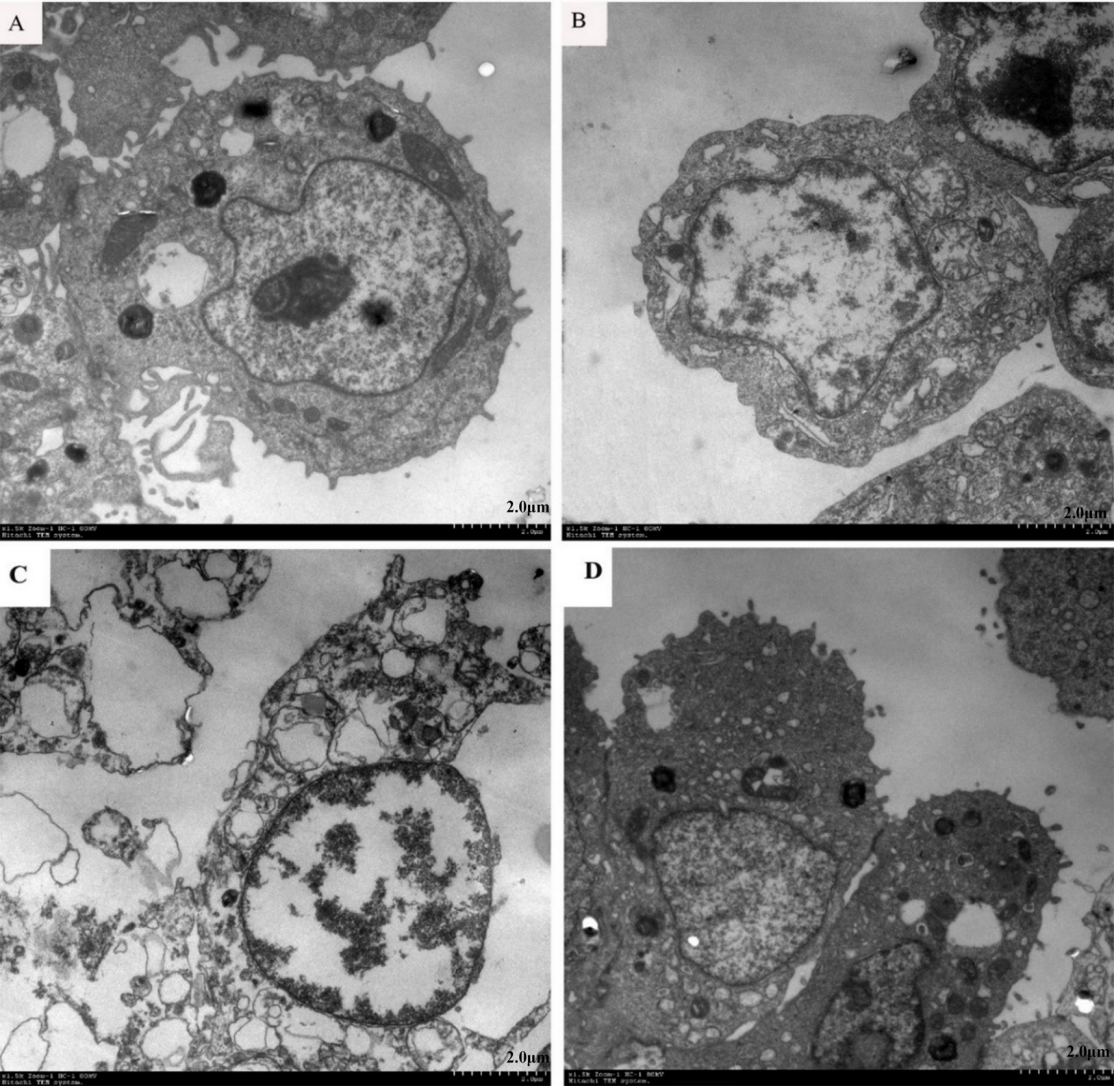
Electron micrographs of CP II cells. (A) Viable cell (untreated); (B) Apoptotic cell (treated with APEC for 4h [MOI = 100]); (C) Necrotic cells (treated with APEC for 4h [MOI = 100]); (D) Cells treated with DH5α for 4h [MOI = 100].

Electron microscopy of APEC-infected CP-II cells revealed morphological changes consistent with induction of both apoptosis and necrosis. Apoptotic cells (Fig 6B) were characterized by membrane blebbing and nuclear condensation, while necrotic cells were typically larger and lighter with plasma membrane lesions (Fig 6C). Electron microscopy of DH5α-infected CP-II cells (Fig 6D) shows that the cell morphology was intact and had no significant difference compared with the viable cell (untreated) group (Fig 6A).

**Fig 7:**
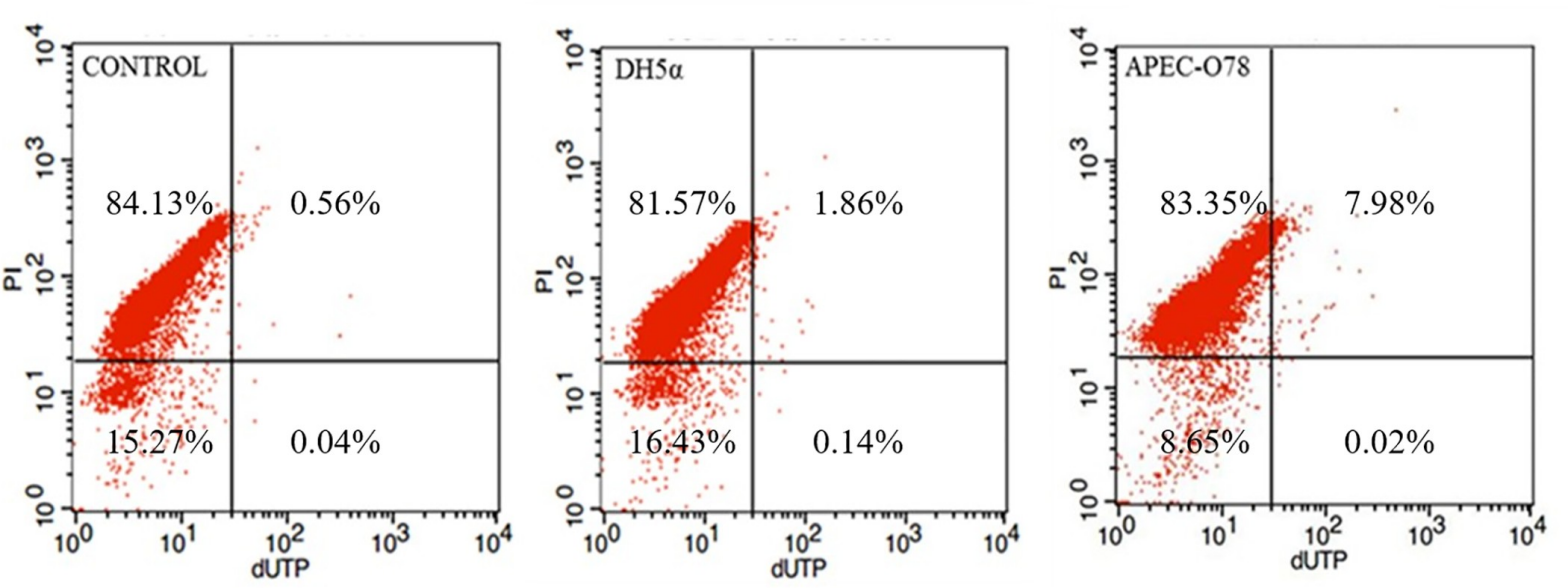
The apoptosis rate of APEC-O78-induced CP II cells. The CP II cells infected by APEC-O78 and DH5α were at a MOI of 100 for 4h. Then the cells were stained using the TransDetect Annexin V-FITC/PI Cell Apoptosis Detection Kit. Finally, the cells were analyzed with a FACSCalibur flow cytometer.

Tunnel results (Fig 7) showed that percentages of apoptotic cells in DH5α-infected CP-II cells (4-h incubation) were 2% and in control were 0.6%, while percentages of apoptotic cells in APEC-infected CP-II cells (4-h incubation) were 8%.

### Changes in the expression levels of related immune resistance genes of CP II cells infected by APEC- O78

**Fig 8:**
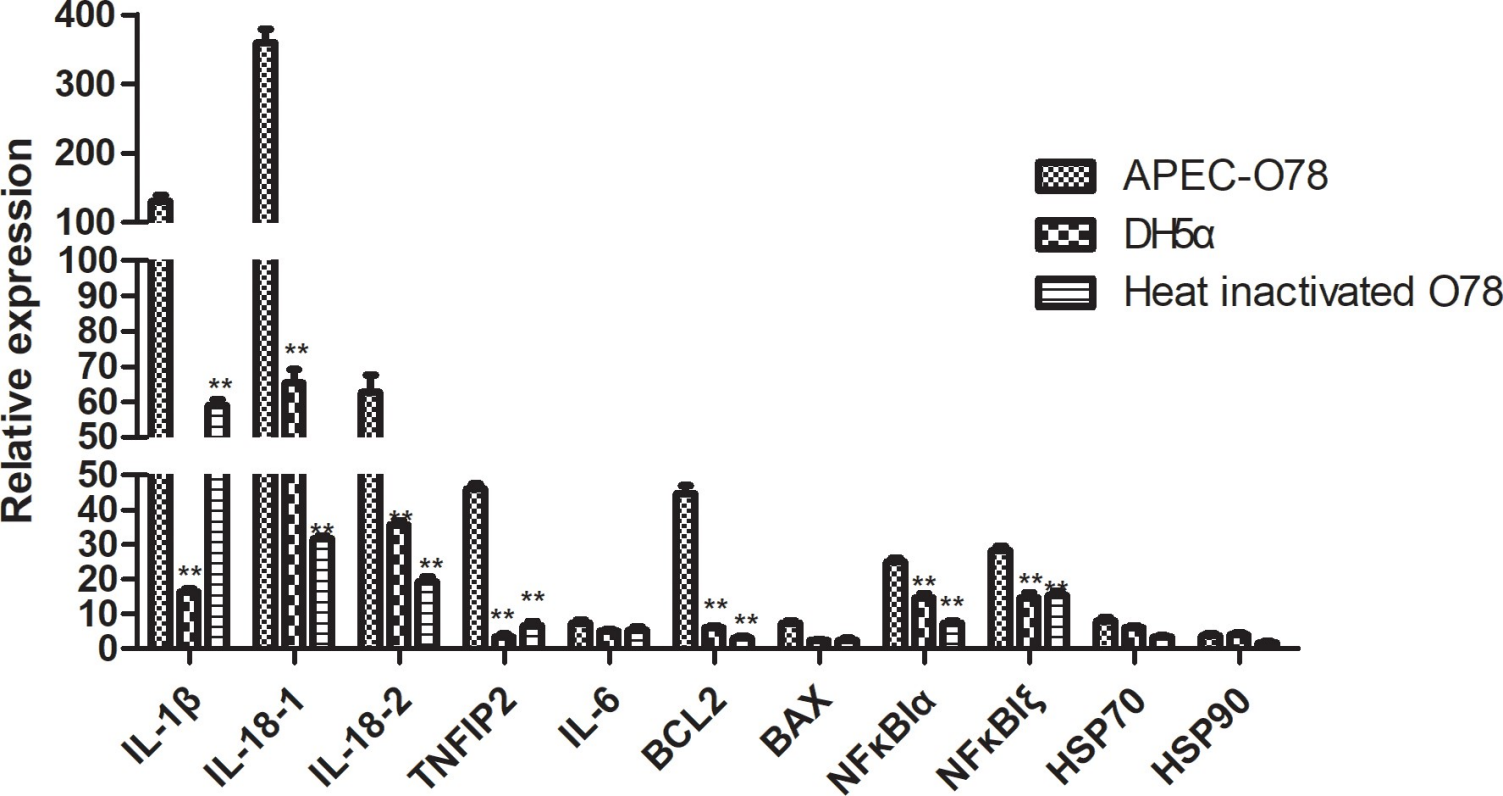
Changes in the expression levels of related immune resistance genes of CP II cells infected by APEC- O78. After DH5α, APEC-O78 and heat inactivated APEC-O78 treated for 4 h with CP II cells, the expression levels of related immune resistance genes of CP II cells was detected by qRT-PCR. *P < 0.05 and **P < 0.01 are significantly different from the DH5α and heat inactivated APEC-O78 group.

The qRT-PCR results (Fig 8) showed that APEC-infected CP-II cells significantly affected the mRNA levels of seven genes (IL-1β, IL8-1, IL8-2, TNIP2, NFKBIξ, NFKBIα and BCL2), comparing treated to DH5a and heat inactivated O78.The expression of five genes (IL-6, Bax, Hsp70, Hsp90, and TLR4) was not significantly changed in each group.

## Discussion

In recent years, research about bacteria-host interactions were rapidly increasing. However, most reports concentrate on studying gene expression profile in immunologically related tissues and cell such as spleen, bursa, HD11 macrophages, and leukocyte [18–20, 25]. To the best of our knowledge, the current is the first report about the remarked differential responses of CP II cells to the infection of APEC-O78. After infection of APEC-O78, 1390 differentially expressed genes were identified in the CP II cells. These top 15 up-regulated biological process terms included regulation of Toll signaling pathway, apoptotic process, and intracellular signal transduction. Although any significant up-regulation in the mRNA levels of Toll-like receptor (TLR) 1–7 and TLR9 was not found in this study, we found that some genes involved in TLR4 and TLR9 pathways were up-regulated. The up-regulated genes of TLR4 pathways might be related to endotoxin stimulation. In clinical studies, TLR9 agonists had a good effect on the treatment of pathogenic infections, allergies and malignant neoplasms[26].

Further, KEGG prediction and GO analysis showed that the significantly enriched pathways were mainly related to NF-kappa B signaling pathway, apoptosis pathway, tight junction, cytokine-cytokine receptor interaction, and other pathways. NF-kappa B signaling pathway mediates immunity, inflammation, and other important cellular functions[27]. Evidence showed that various NF-κB defects are indicated by immune dysregulation, with features of autoimmunity and inflammation[28]. NF-κB is a transcription factor that involved in the expression of inflammatory cytokines, chemokines, and growth factors genes and is responsible for the immune and inflammatory response[29, 30]. It is thus suggested that NF-κB inhibition alleviates the inflammatory response, and various natural herbs and chemical agent affect anti-inflammatory response by inhibiting NF-κB signaling pathway[31–33]. Other studies also suggested that NF-κB has an ability to facilitate different aspects of development and selection of T-regulatory cells in lymphoid and non-lymphoid tissues by regulating the expression of various TNF receptor superfamily members[34]. In addition, most studies have shown that NF-kappa B is important in the induction of the apoptotic pathway[35, 36].

Apoptosis is an essential part of normal development in multicellular organisms, but it is also induced by disease [37]. When pathogens infect the host, the pathogen could ‘‘kidnap” the host’s apoptotic pathway to promote its pathogenesis [38]. It is reported that APEC-induced apoptosis occurred in macrophages, challenged-susceptible birds and chicken embryo intestinal cells [19, 39–41]. In order to test whether APEC-O78 could induce the apoptosis of CP II cells, we used TEM scanning electron microscopy and TUNEL flow cytometry to detect APEC-induced apoptosis in CP II cells. We adopted TUNEL flow cytometry on CP II cells, after CP II cells infected with E. coli for 4h. Our result showed that there was 8% apoptotic CP II cells in the infected group. At the same time, the results of qRT-PCR showed that O78 can induce higher gene expression of BCL2 and IL-8, IL -β, TNIP2 related in immune resistance comparing to DH5a and heat inactivated O78. Moreover, it confirmed that APEC-O78 cannot induced CP II cell apoptosis through modulating BAX and BCL2 expression. At the same time, we can observe that O78, DH5a and heat inactivated O78 have different levels of impact on transcription factor NF-κB pathway to varying degrees.

Recently, the EPEC T3SS effector protein has been shown to regulate apoptosis and promote host cell survival, such as T3SS effector proteins such as EspZ, NleH1 and NleH2[42, 43]. Some studies show that EPEC can activate at least three separate anti-apoptotic pathways, such as tryptophan kinase pathway, protein kinase C pathway and NF-κB transcription factor, which may induce IL-8 expression and activate anti-apoptotic pathway. In this study, we also found that IL8L1 (28.57 fold change) and IL8L2 (25.47 fold change)expression was significantly up-regulated after APEC infection. The epithelial cell premature death is not conducive to bacterial adhesion and infection, suggesting that APEC and EPEC similar, there is a defense mechanism, which can delay the host cell apoptosis, prolong the adhesion time, prevent infected cells from being cleared by the host. APEC-O78 does not induce CP II cells to produce strong apoptosis, as previously reported that *Salmonella enteritidis* can not trigger chicken embryo fibroblasts apoptosis[44]. Li et al. found that bacterial strains that cause apoptosis and necrosis are selective for cell types[45]. In addition, chicken embryo fibroblasts and CP II cells are non-phagocytic cells. For phagocytic cells, APEC infection with HD11 macrophages, can cause severe apoptosis. Percentages of apoptotic cells in APEC-infected CP II cells (4-h incubation) were 8% in Tunnel, are similar to percentages of apoptotic cells (12%) in flow cytometric analysis of annexin V-FITC and PI (see S4 Fig). We report here that APEC induces a mixture of apoptosis and necrosis in CP II cells. but this was much weaker than APEC-induced HD11 macrophages apoptosis. Bastiani et al. found resident murine peritoneal macrophages infected in vitro with an avian strain of E. coli underwent apoptosis 4 h after infection (55.6% of apoptosis in infected cells versus 3.5% in non-infected cells) [40].

Ama et al. found 19 genes differentially expressed in chicken embryo fibroblasts after infection with *Salmonella enteritidis* [21]. They also found that IL8L1, IL8L2, CSF3, and IL1β were up-regulated, but the fold changes were far smaller. APEC-O78 can strongly induce the apoptosis of CP II cells, but *Salmonella enteritidis* can not trigger the apoptosis of the chicken embryo fibroblasts[45].

It is likely that the main cause for the differences is the different gram-negative bacteria. In addition, both chicken embryo fibroblasts and CP II cells belong to the non-phagocytic cells. For phagocytic cells such as HD11 macrophages exposed to APEC, the differentially regulated signal transduction pathways are similar [20]. The induction of IL8 and IL1β, and apoptosis in macrophages exposed to APEC-O78 were also found. However, IL-6 in CP II cells exposed to APEC-O78 was not changed in this study.

In summary, gene expression profile of CP II cells infected with APEC-O78 showed not only changes of inflammation and immune response pathways, but also changes of apoptosis pathway. At present, we do not know whether the induction of CP II cells apoptosis is restricted to APEC-O78 or is common to other APEC isolates. The investigation of inhibition of APEC-O78 induced apoptosis in CP II cells could provide a new therapeutic idea in the treatment of colibacillosis in poultry.

## Supporting information

S1 Fig The cell purity was tested by flow cytometry and TEM. A. Osmiophilic lamellar bodies of chicken type II pneumocytes observed by TEM (magnification 2500×). The arrow shows an osmiophilic lamellar body. B. Chicken type II pneumocytes purity was analyzed by flow cytometry using FITC-CD74(green line)as a marker for Chicken type II pneumocytes and negative control(black line).
(PDF)

S2 Fig The samples quality score. The horizontal axis represents the base number or base Range, the vertical axis represents the mass fraction. Quality scores greater than 20 indicates that the mapping accuracy is greater than 99%. The data can be used for subsequent analysis.
(PDF)

S3 Fig Clustering heatmap showing the differential mRNAs. Each column represents one sample and each row represents one differential mRNA.
(PDF)

S4 Fig Flow cytometric analysis of annexin V-FITC/PI double-staining. (A) un-treated chicken type II pneumocytes (the un-treated control group); (B) Chicken type II pneumocytes were treated for 4 h with APEC-O78 at a multiplicity of infection (MOI) = 100. Cells were incubated with Annexin V-FITC in a buffer containing propidium iodide (PI) and analyzed by flow cytometry.
(PDF)

S1 Table List of qRT-PCR primers used in this study. (PDF)

S2 Table Mapping statistics. (PDF)

S3 Table The RNA-seq data and data analysis. (XLS)

S4 Table Selected data from the RNA-seq of chicken type II pneumocyte, with a focus on transcripts representing gallus gallus only. (PDF)
