# Supplementary figures and images for: RNA-seq profiles of chicken type II pneumocyte in response to Escherichia coli infection
Source: PLoS One. 2019 Jun 5;14(6):e0217438. doi: 10.1371/journal.pone.0217438 (PMC6550405; doi:10.1371/journal.pone.0217438)

A

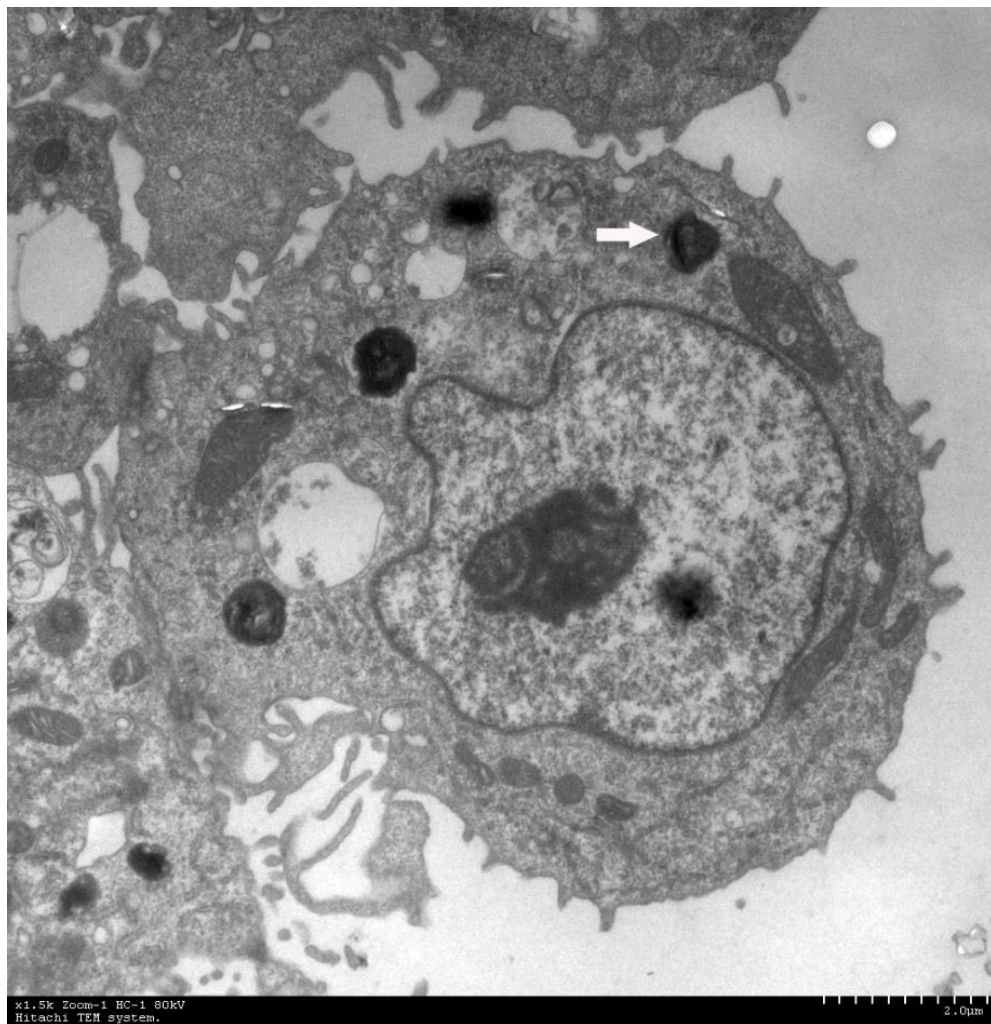

B

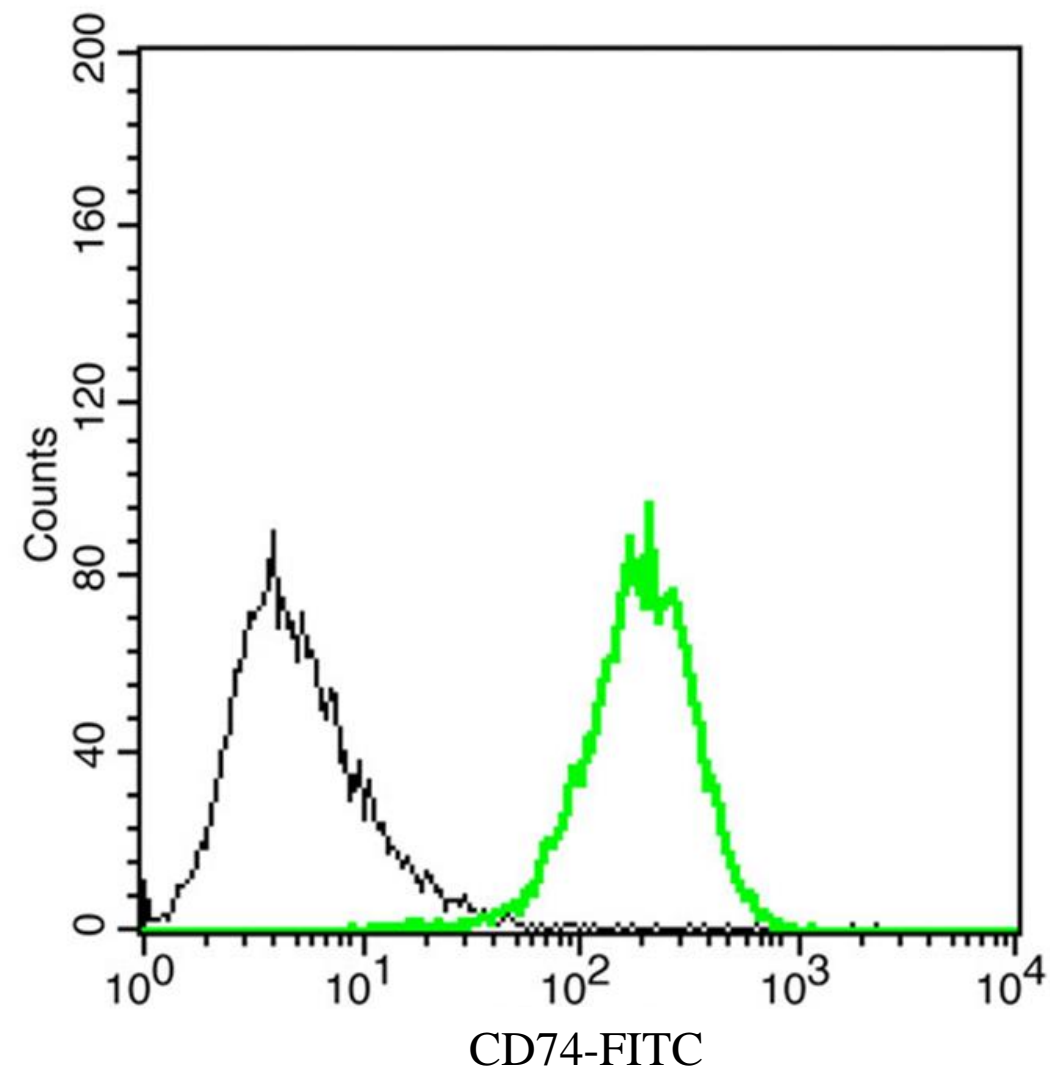

Supplement: S1 Fig — A. Osmiophilic lamellar bodies of chicken type II pneumocytes observed by TEM (magnification 2500×). The arrow shows an osmiophilic lamellar body. B. Chicken type II pneumocytes purity was analyzed by flow cytometry using FITC-CD74(green line)as a marker for Chicken type II pneumocytes and negative control(black line). (PDF) [file pone.0217438.s001.pdf]

APEC infection

Control

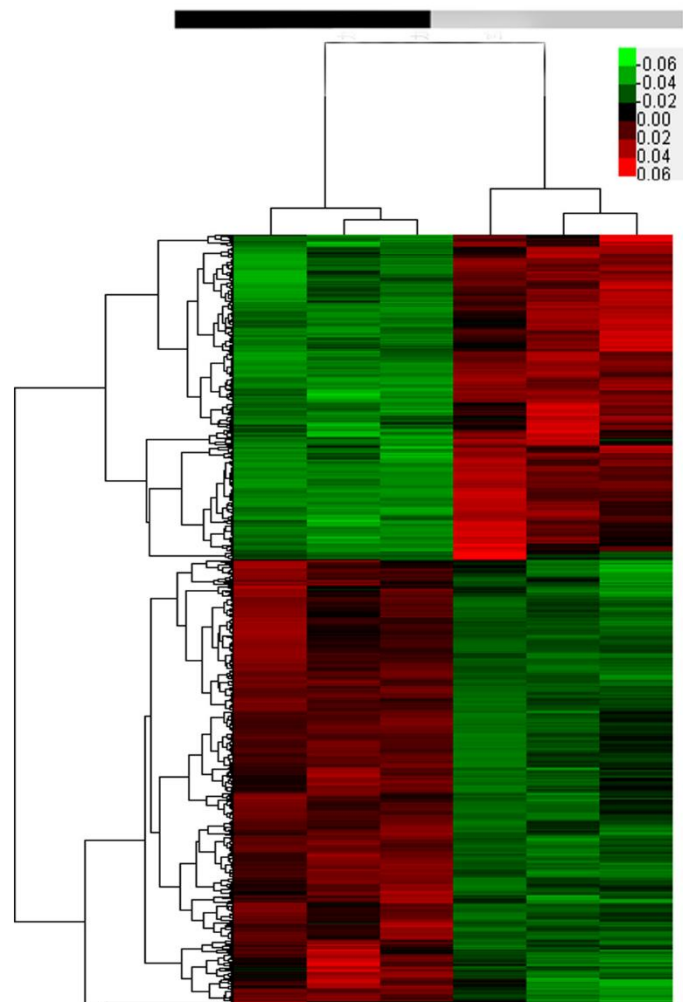

Supplement: S3 Fig — Each column represents one sample and each row represents one differential mRNA. (PDF) [file pone.0217438.s003.pdf]

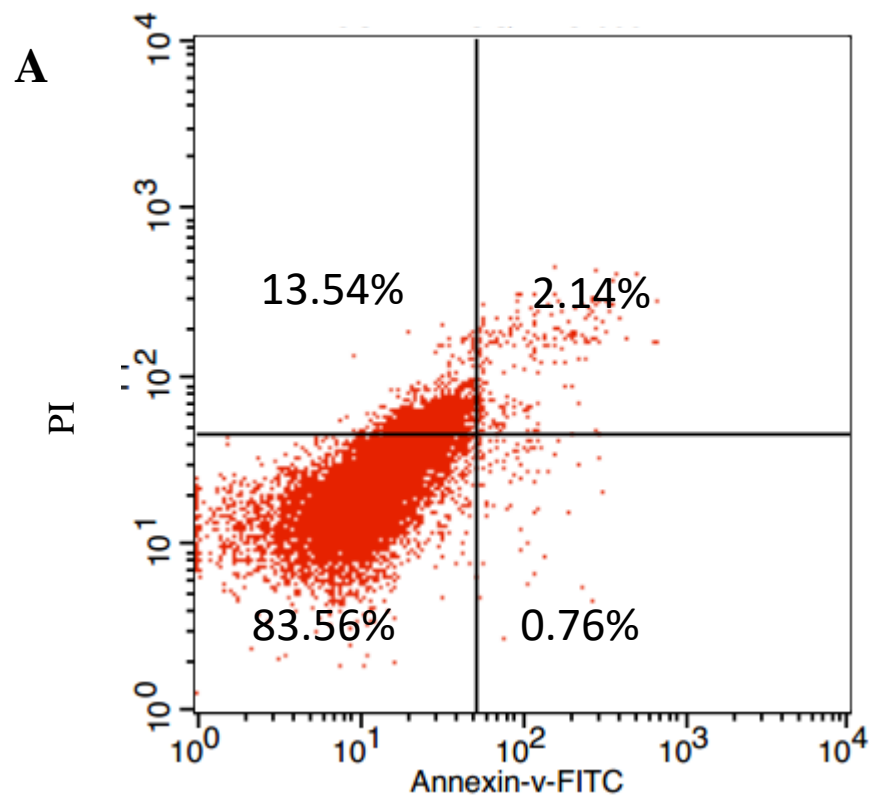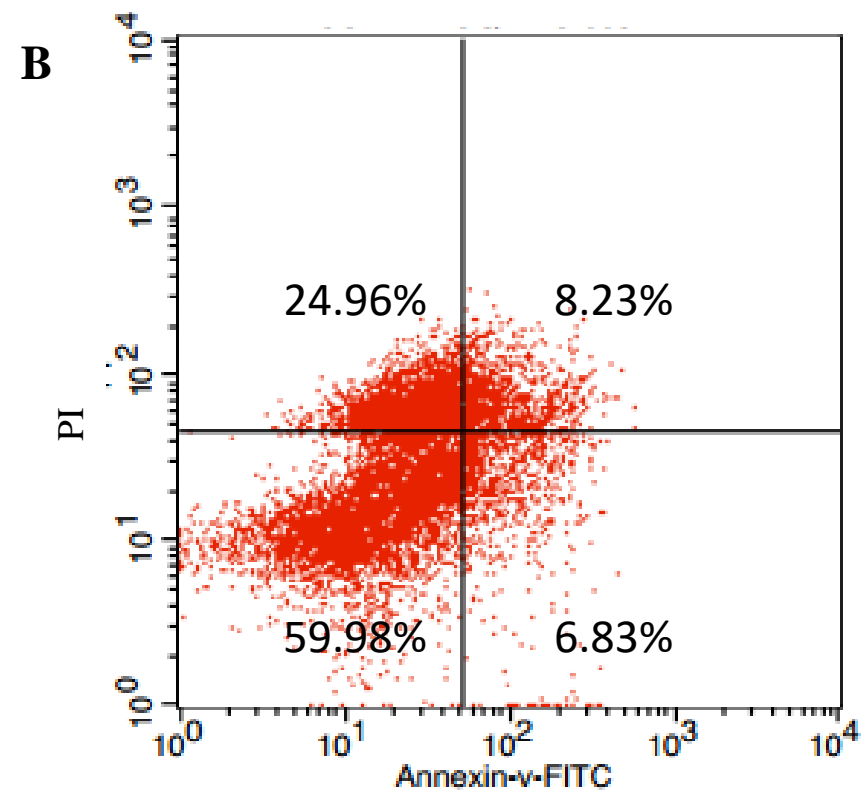

Supplement: S4 Fig — (A) un-treated chicken type II pneumocytes (the un-treated control group); (B) Chicken type II pneumocytes were treated for 4 h with APEC-O78 at a multiplicity of infection (MOI) = 100. Cells were incubated with Annexin V-FITC in a buffer containing propidium iodide (PI) and analyzed by flow cytometry. (PDF) [file pone.0217438.s004.pdf]
