# Supplementary material for: RNA-seq profiles of chicken type II pneumocyte in response to Escherichia coli infection
Source: PLoS One. 2019 Jun 5;14(6):e0217438. doi: 10.1371/journal.pone.0217438 (PMC6550405; doi:10.1371/journal.pone.0217438)
Supplement: S2 Fig — The horizontal axis represents the base number or base Range, the vertical axis represents the mass fraction. Quality scores greater than 20 indicates that the mapping accuracy is greater than 99%. The data can be used for subsequent analysis. (PDF) [file pone.0217438.s002.pdf]

Quality Score

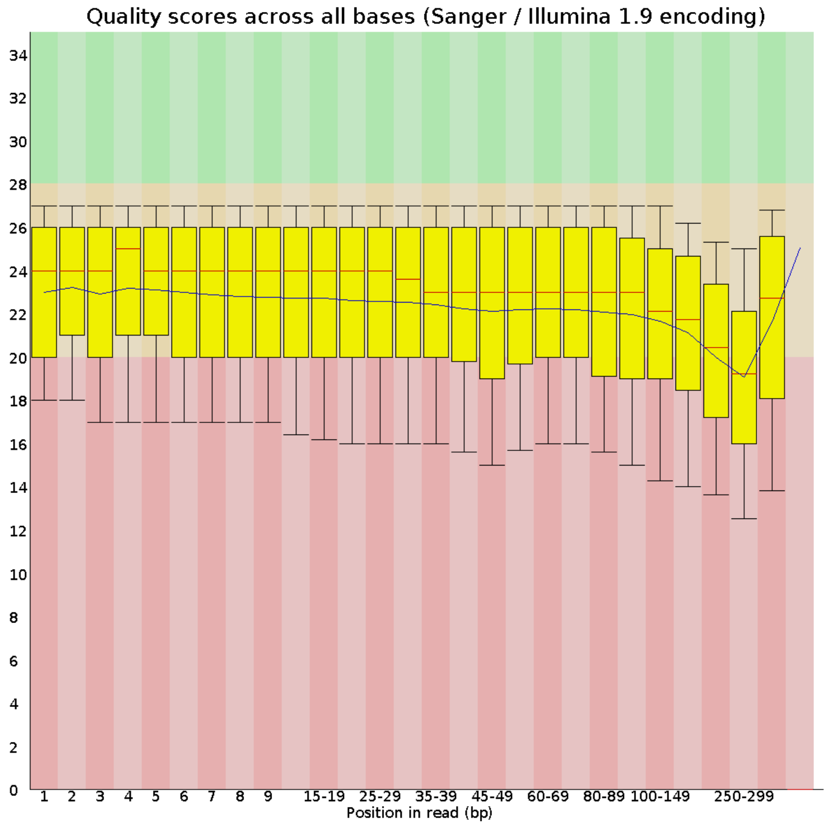

APEC infection 1 QualityScore_AfterFilter

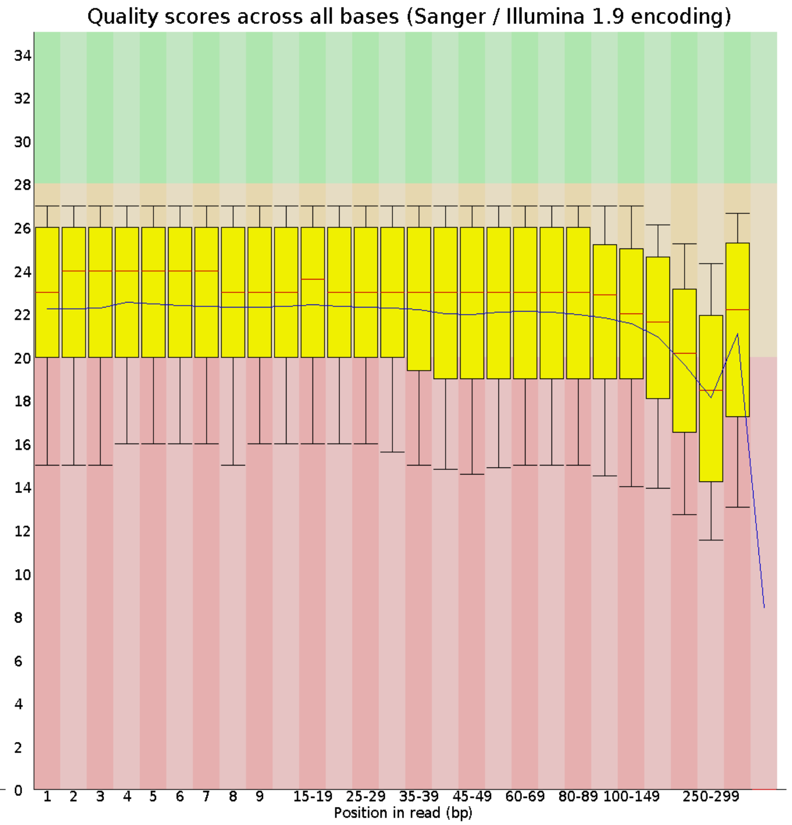

APEC infection 1 QualityScore_BeforeFilter

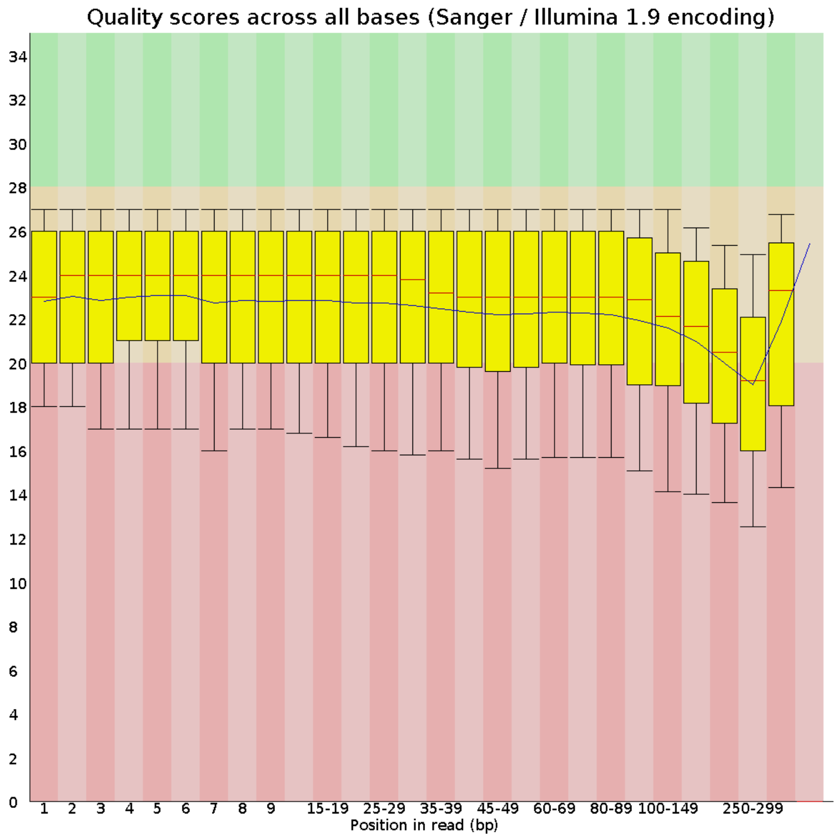

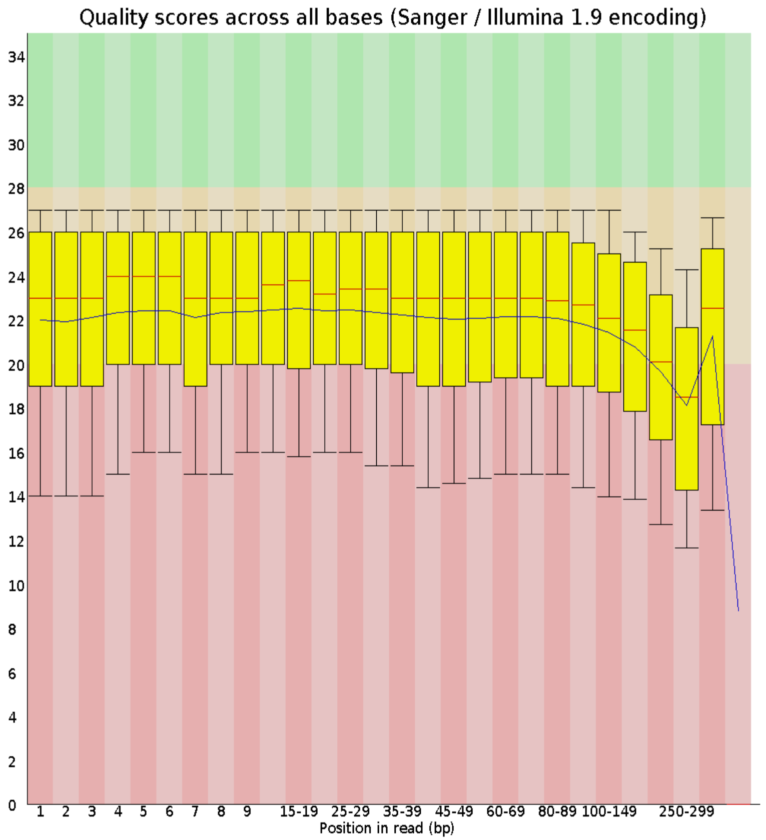

APEC infection2 QualityScore_AfterFilter APEC infection 2 QualityScore_BeforeFilter

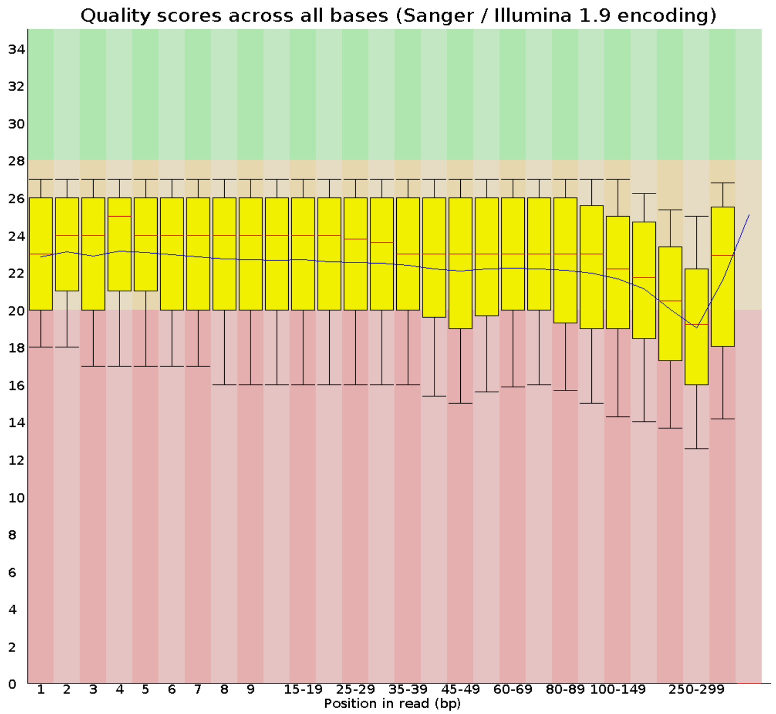

APEC infection 3 QualityScore_AfterFilter

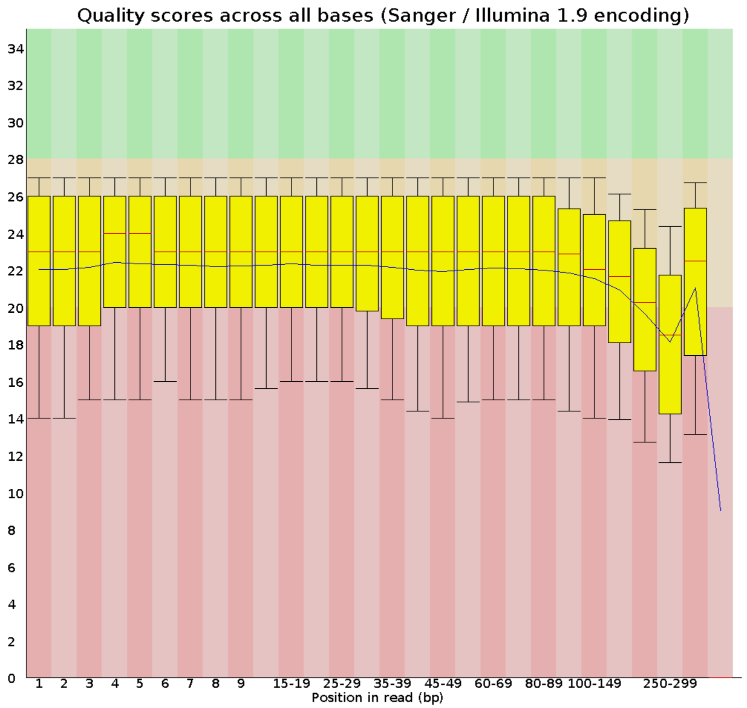

APEC infection 3 QualityScore_BeforeFilter

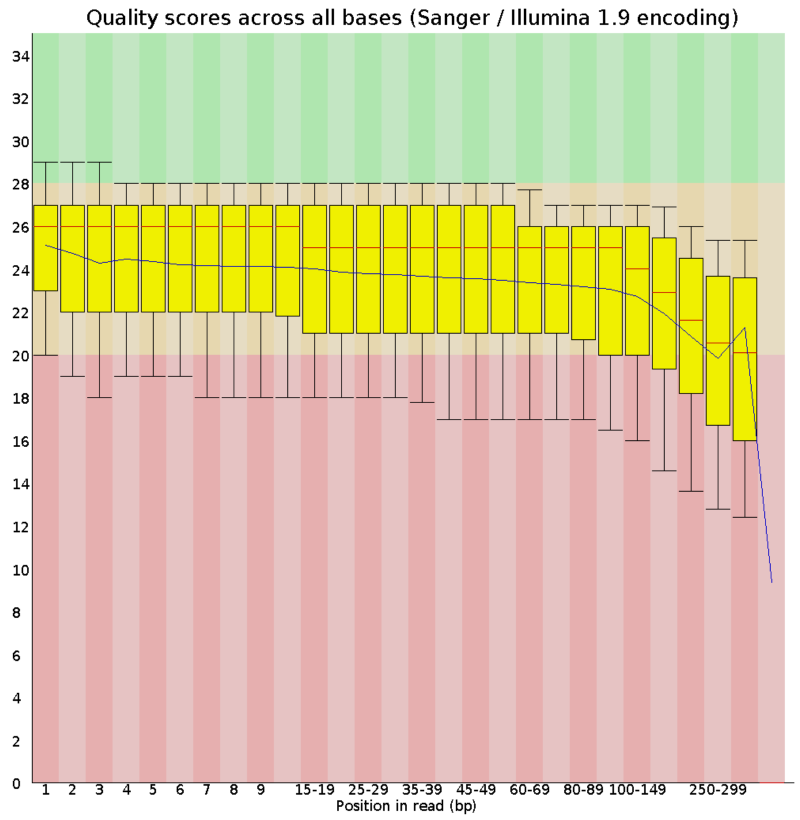

Control1 QualityScore_AfterFilter

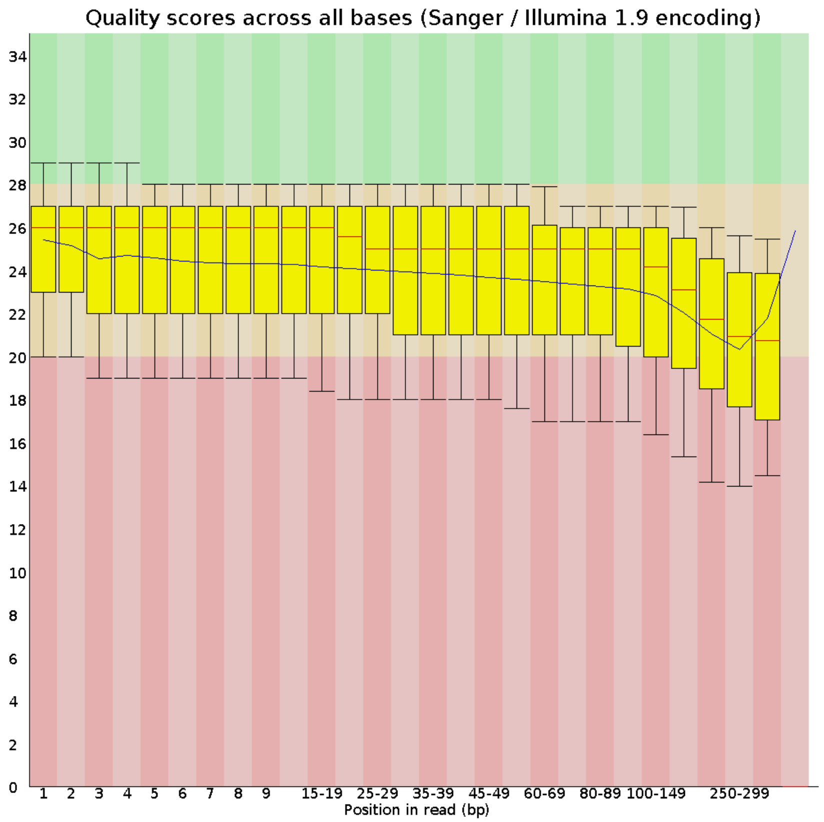

Control1 QualityScore_BeforeFilter

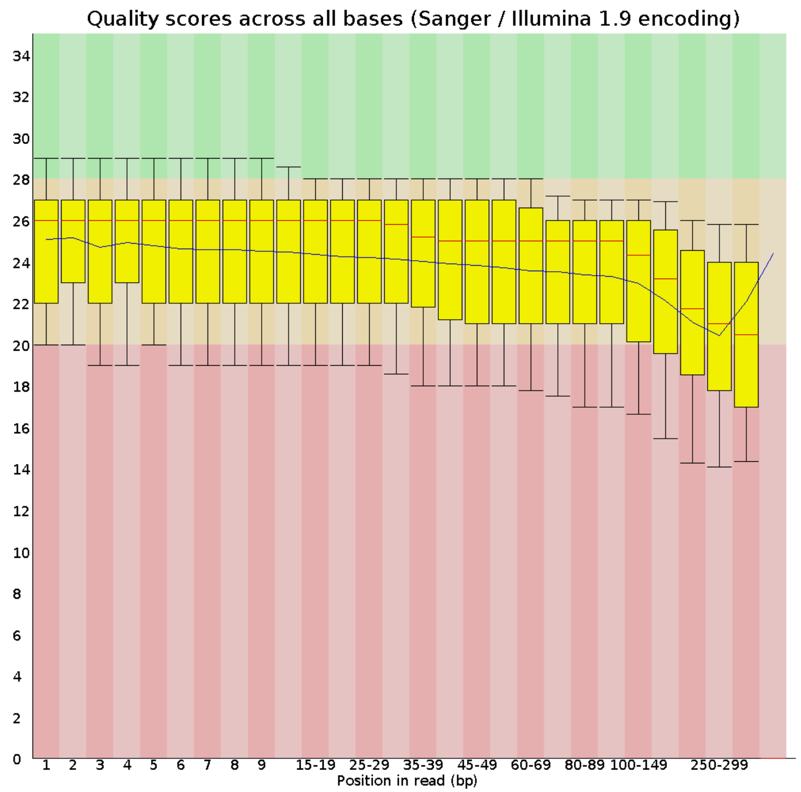

Control2 QualityScore_AfterFilter

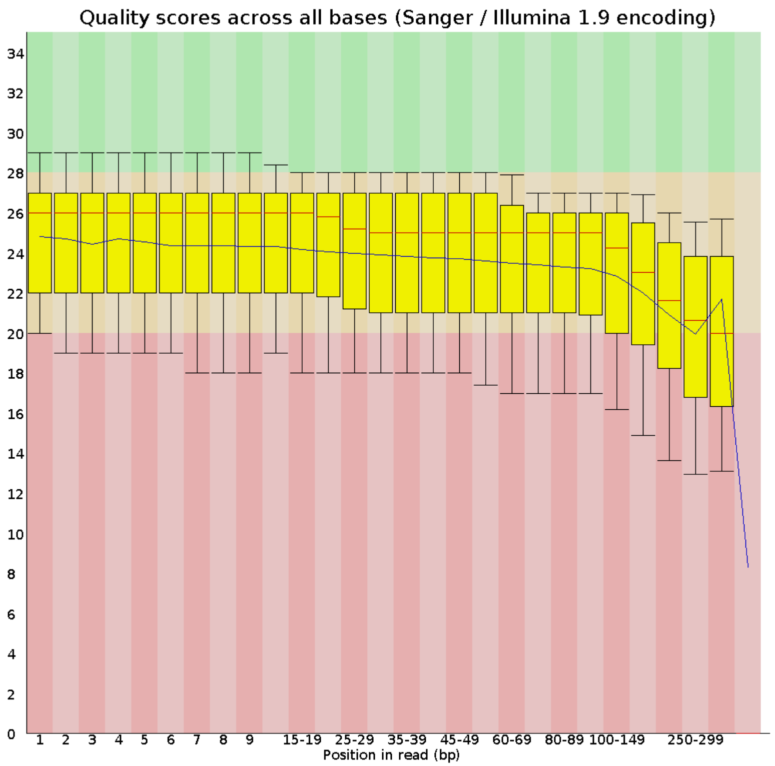

Control2 QualityScore_BeforeFilter

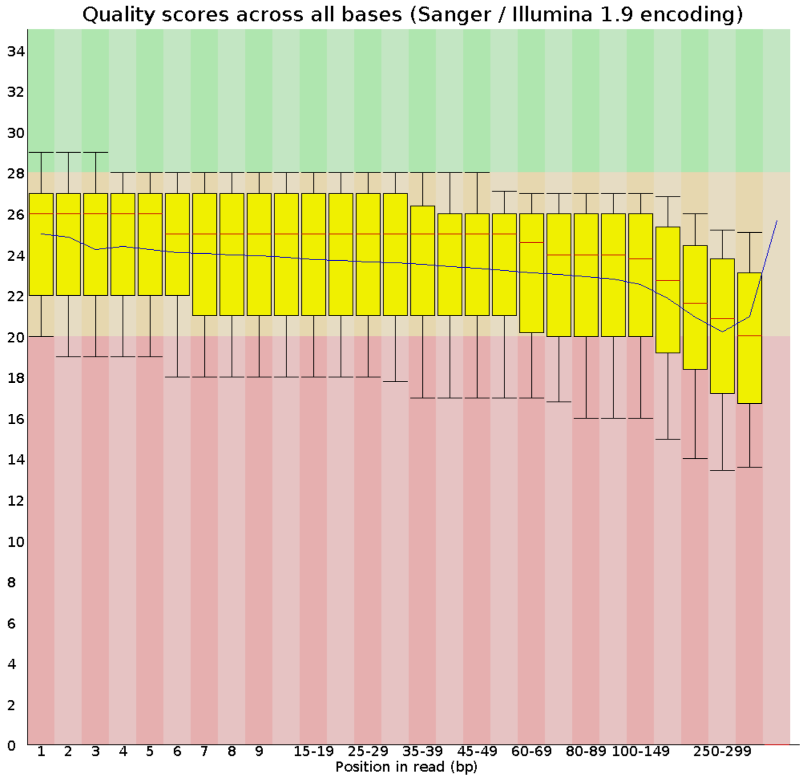

Control3 QualityScore_AfterFilter

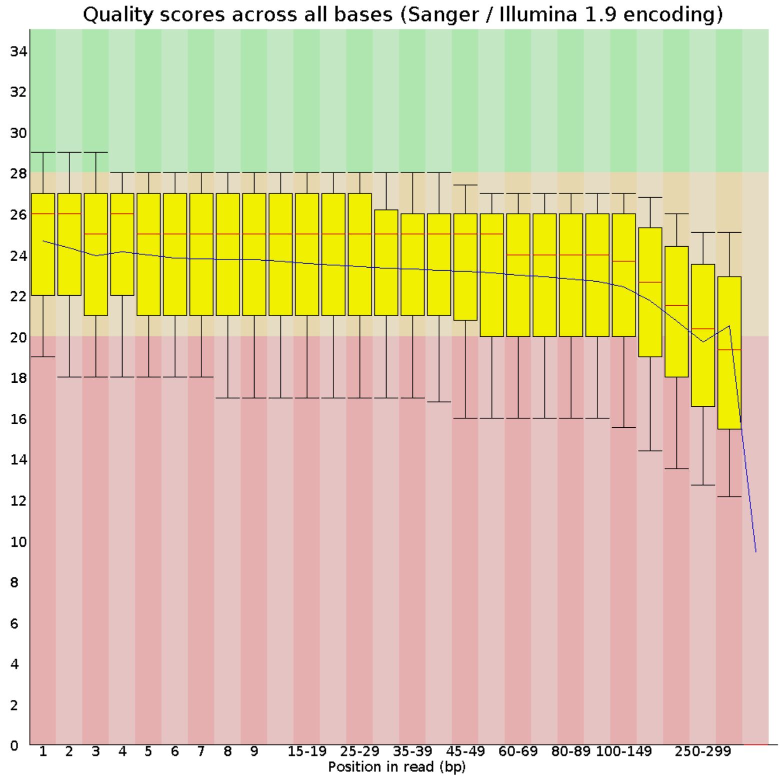

Control3 QualityScore_BeforeFilter

Sequence GC Content

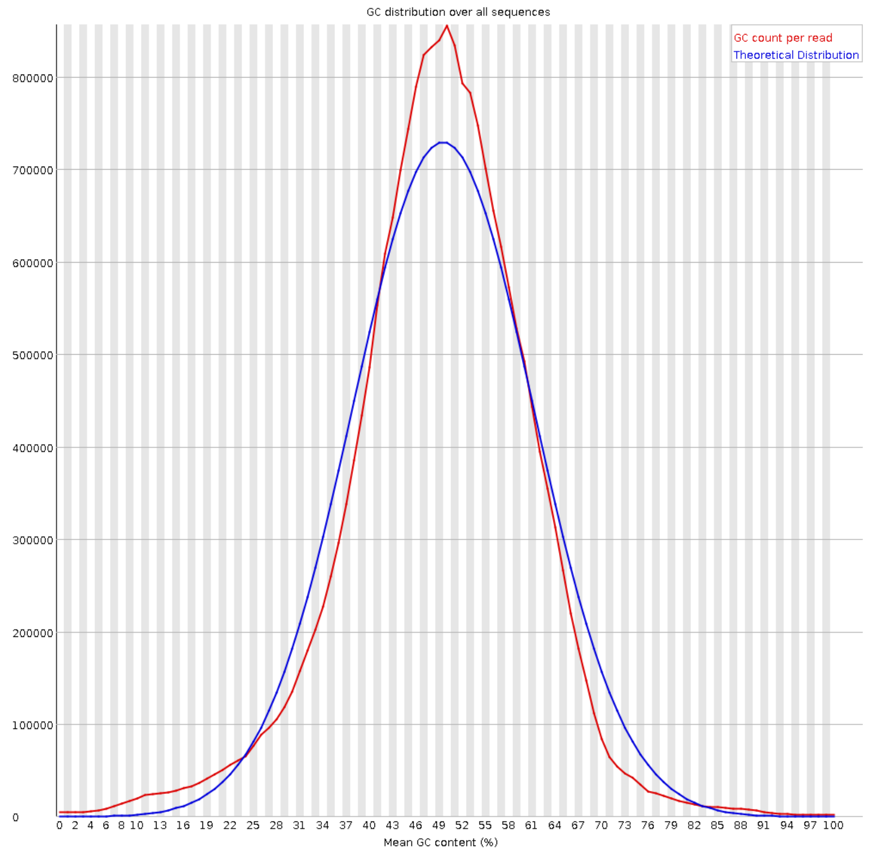

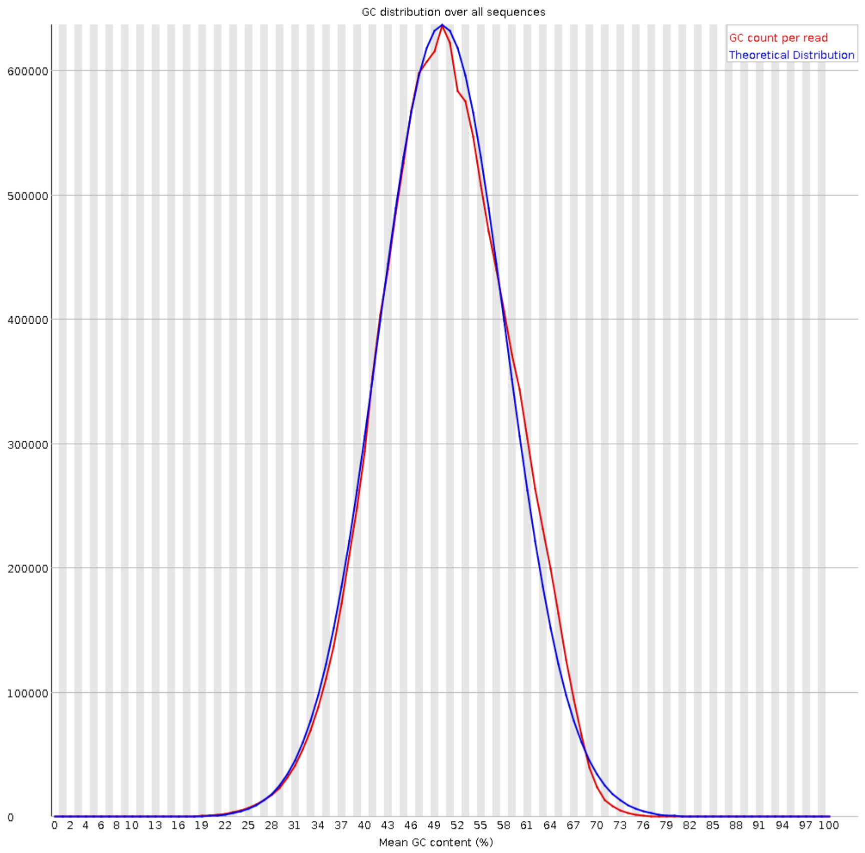

APEC infection1 Sequence GC Content_AfterFilter

APEC infection 1 Sequence GC Content_Before Filter

The horizontal axis represents the average GC content, The vertical axis represents reads. The blue line is the theoretical GC content distribution, the red line is the actual GC content distribution, the actual line distribution in the figure with the normal distribution Closely,it indicates that the results are better, can be used for the following analysis

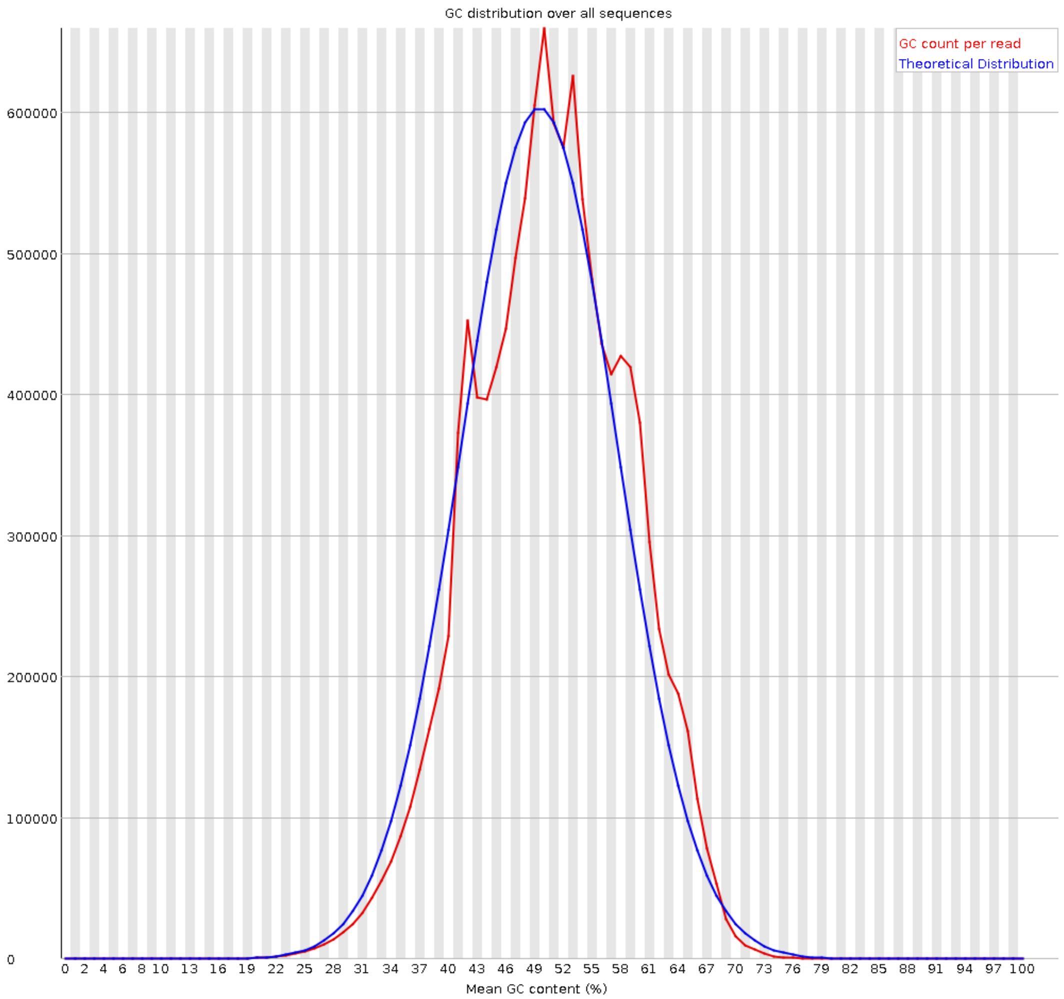

APEC infection2 Sequence GC Content_AfterFilter

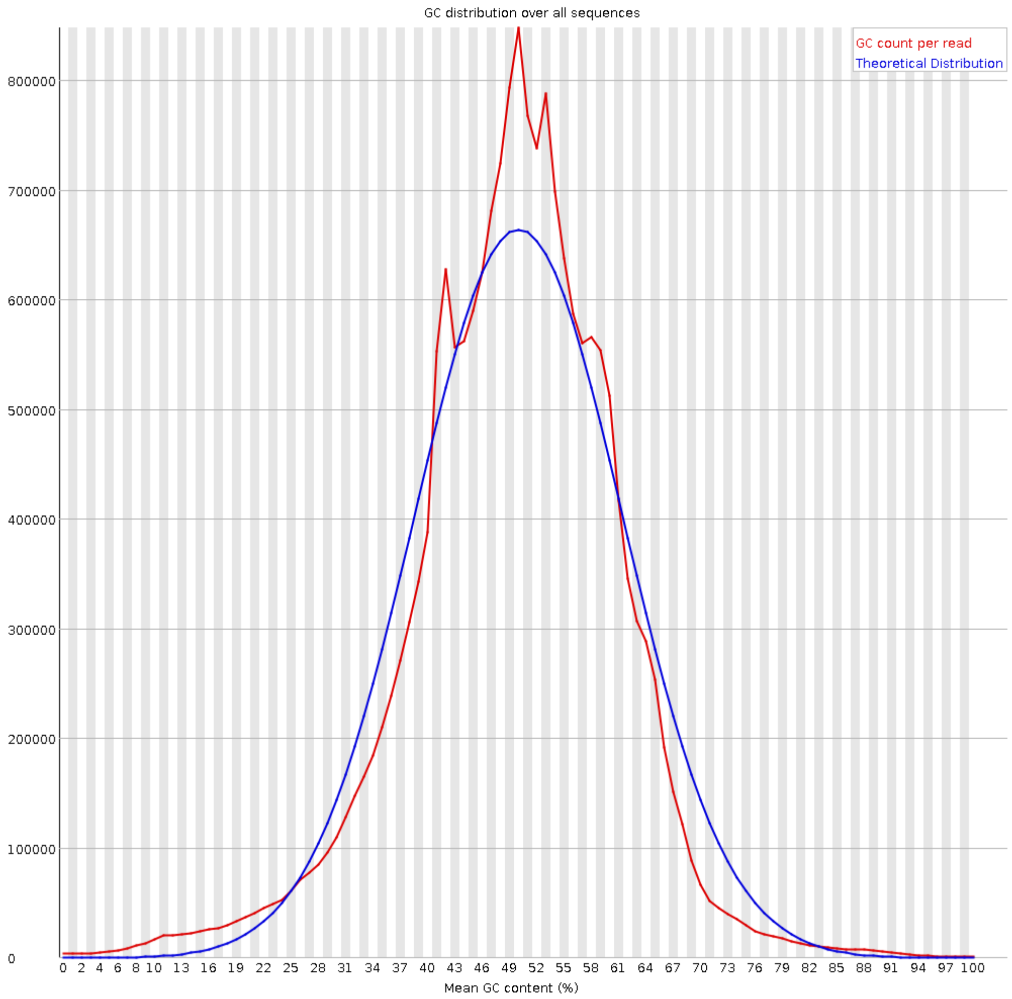

APEC infection 2 Sequence GC Content_Before Filter

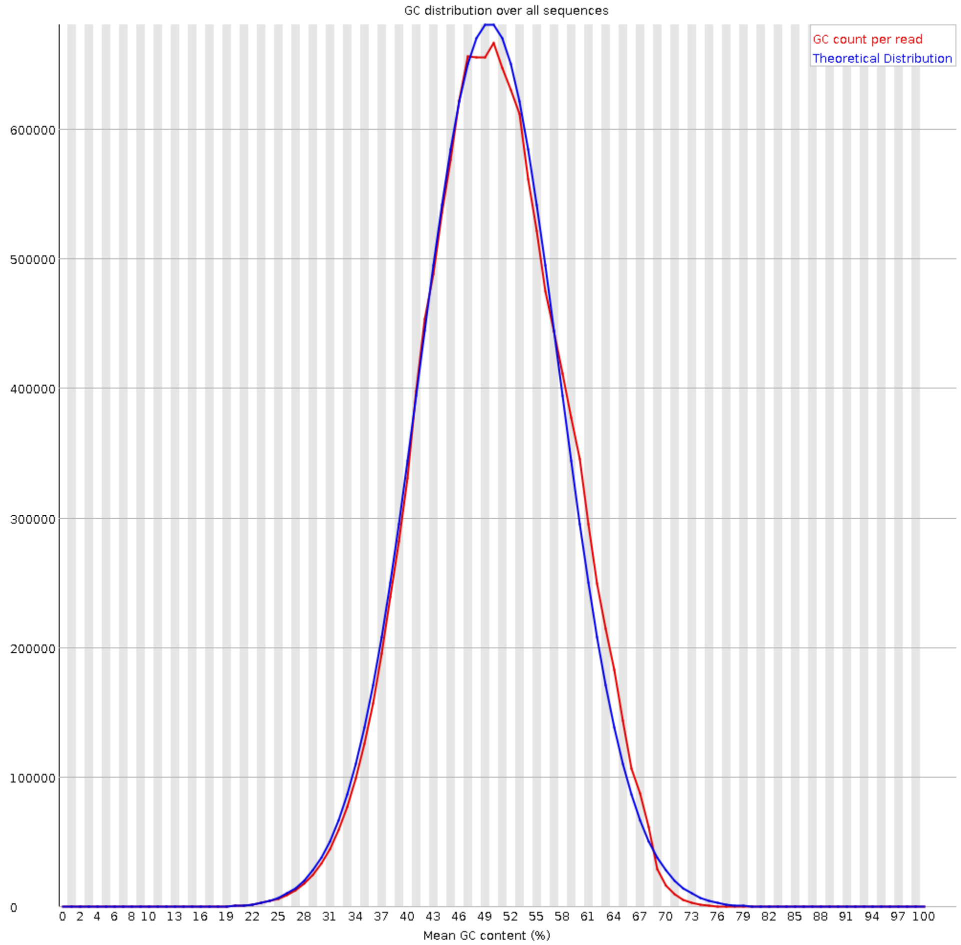

APEC infection3 Sequence GC Content_AfterFilter

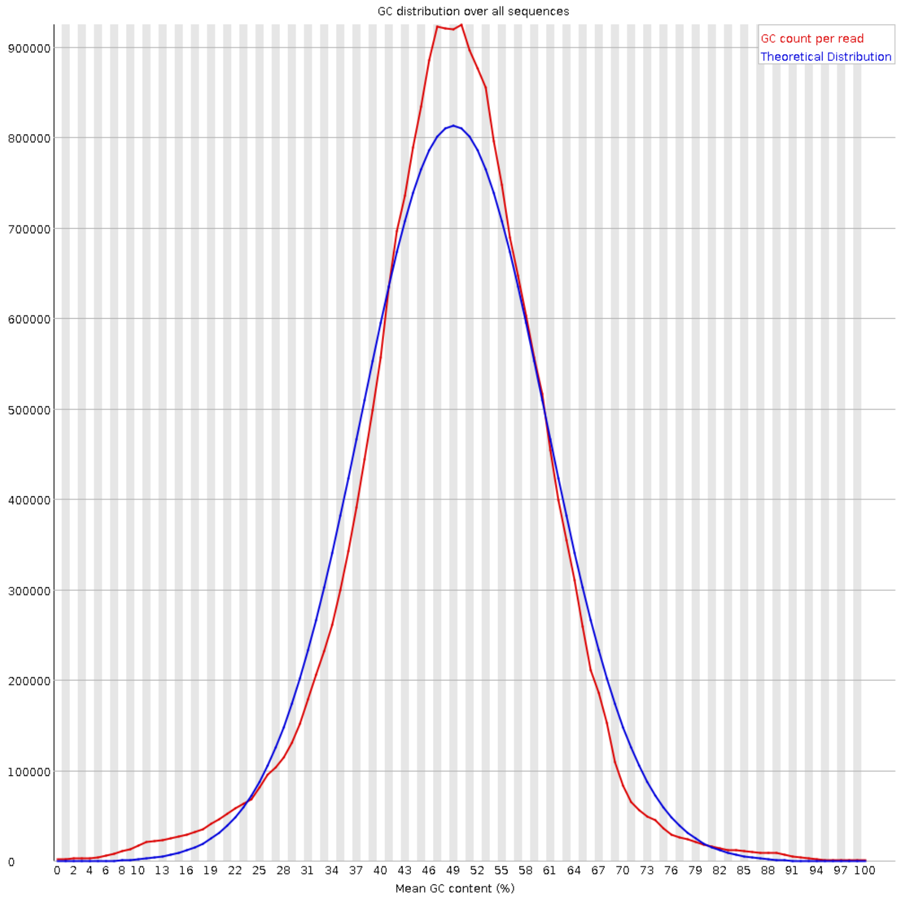

APEC infection 3 Sequence GC Content_Before Filter

S3.Fig 2 The horizontal axis represents the average GC content, vertical The blue line is the theoretical GC content distribution, the red line is the actual GC content distribution, the actual line distribution in the figure with the normal distribution
Close, the results are better, can be used for the following analysis

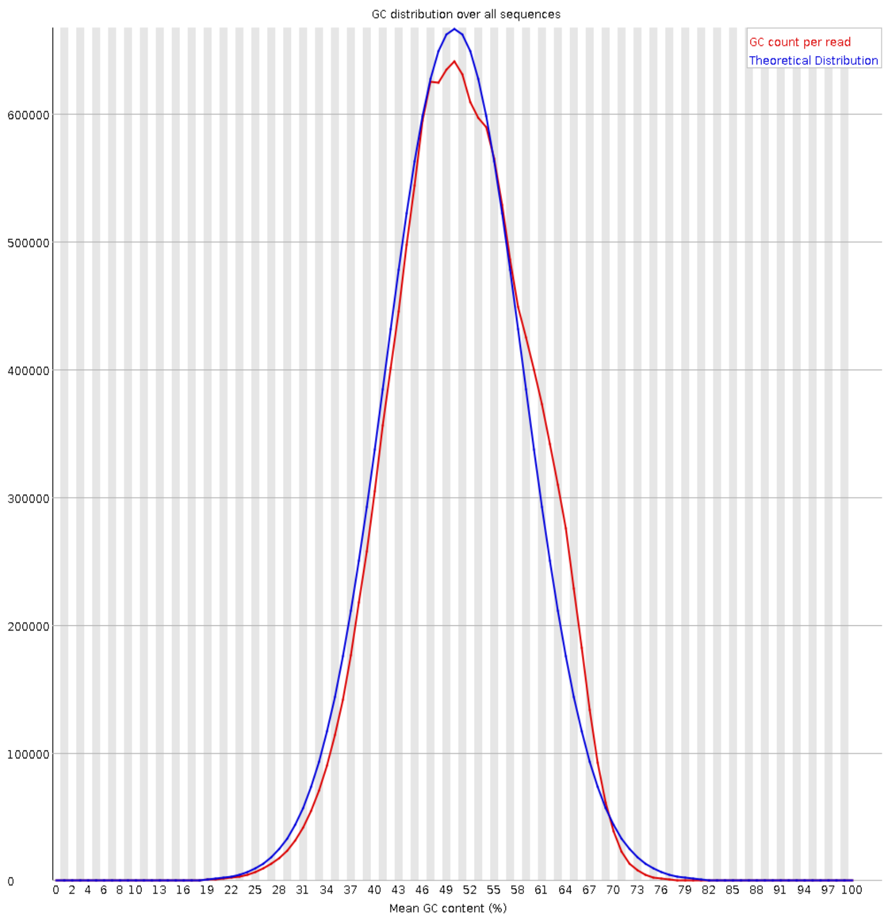

Control 1 Sequence GC Content_AfterFilter

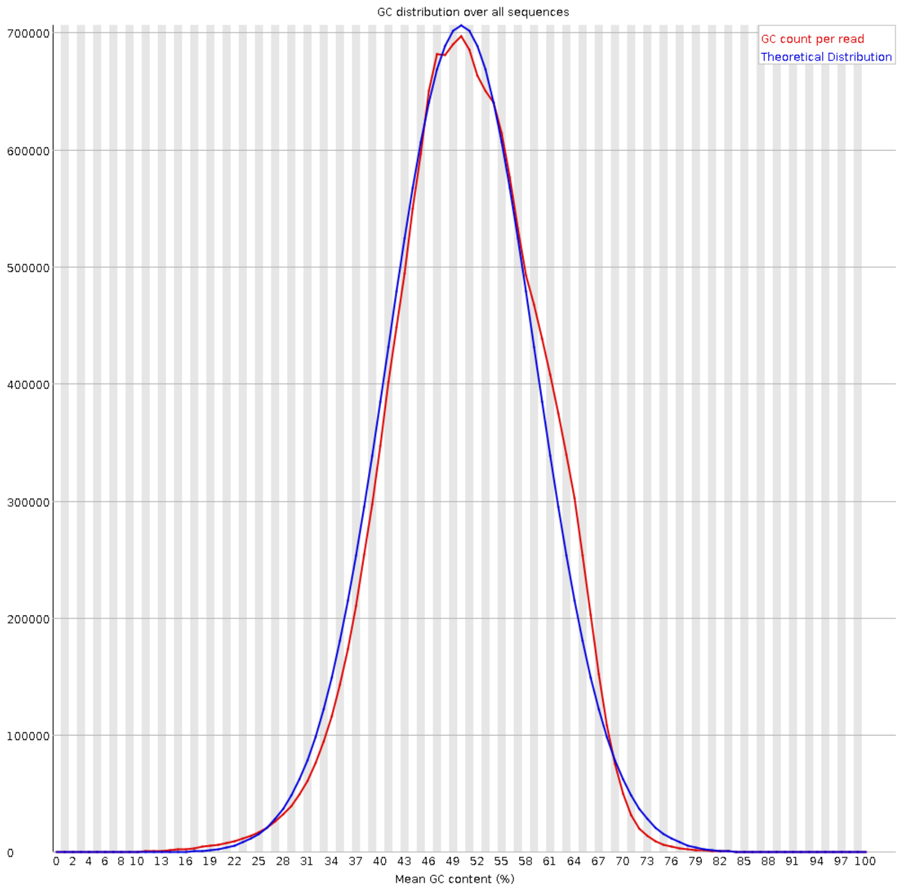

Control 1 Sequence GC Content_Before Filter

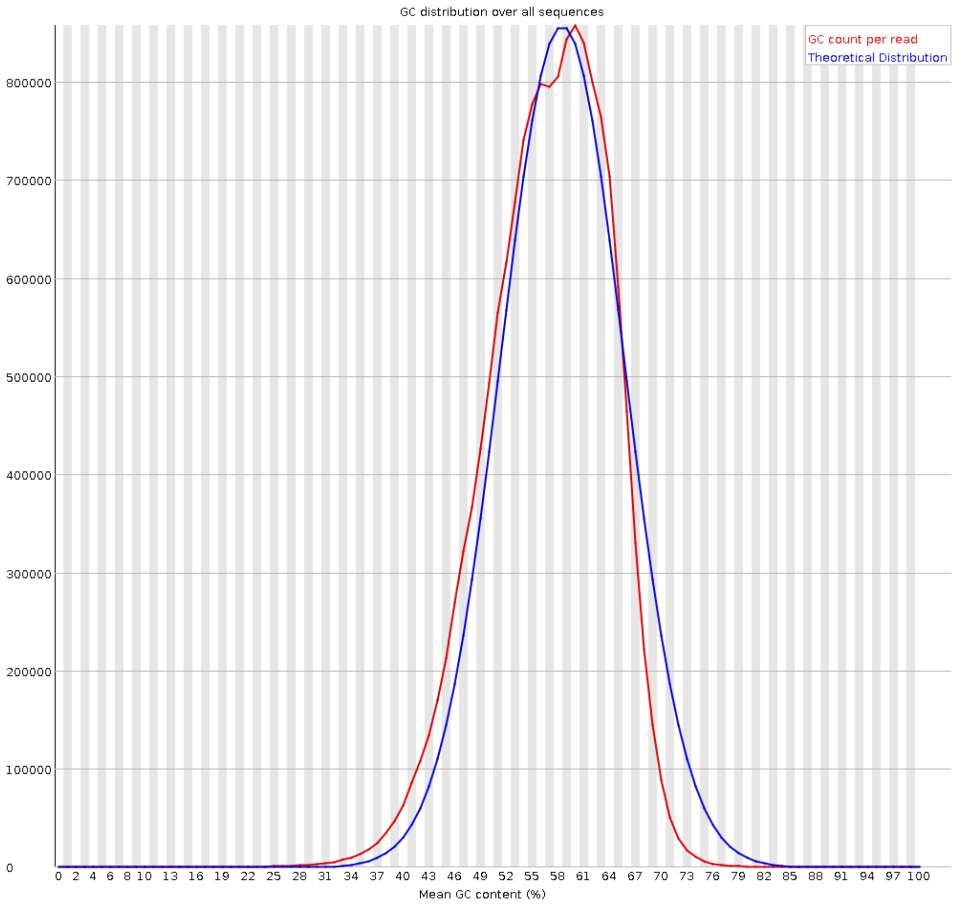

Control 2 Sequence GC Content_AfterFilter

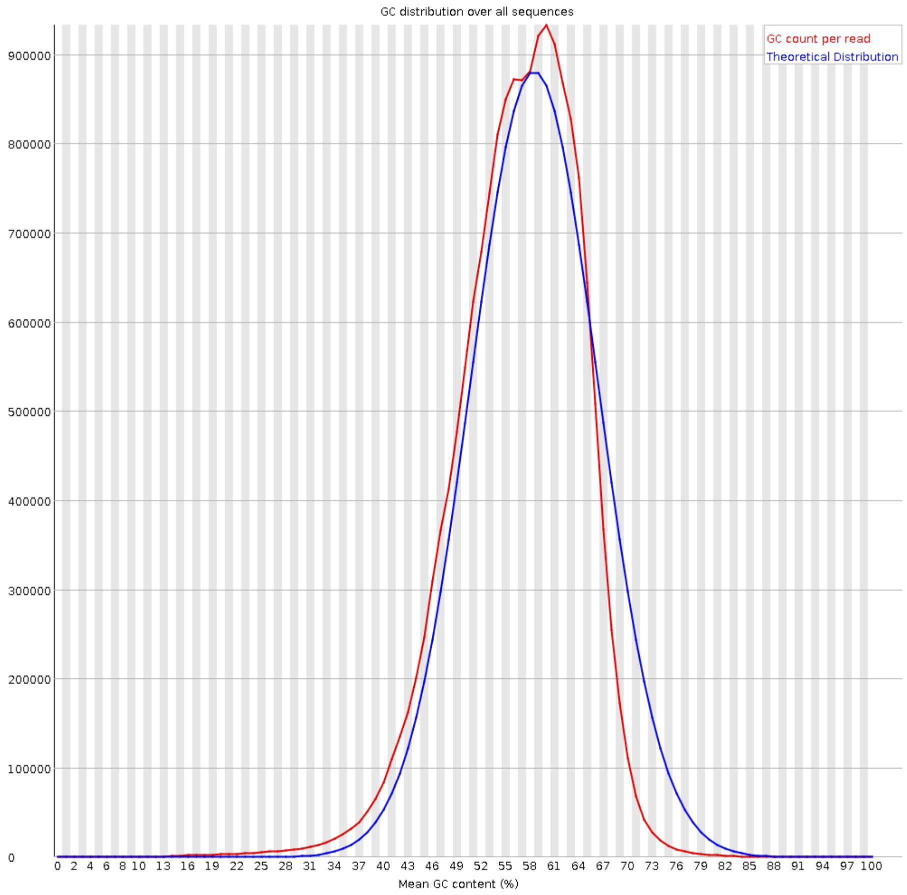

Control 2 Sequence GC Content_Before Filter

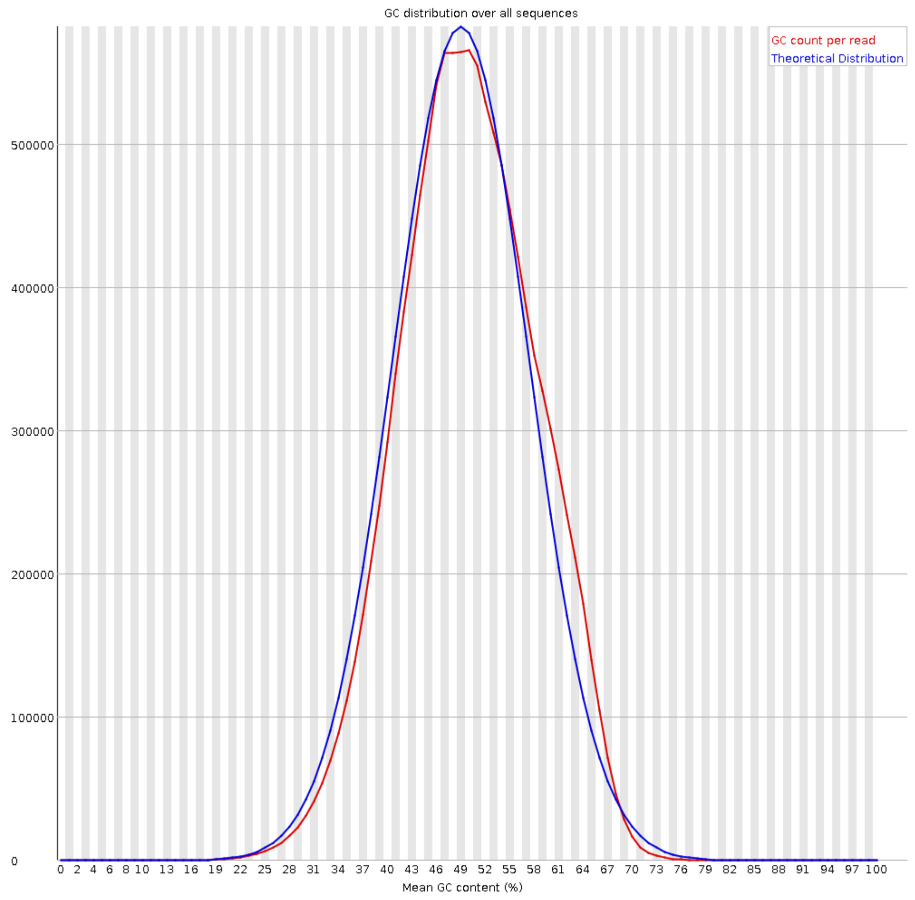

Control 3 Sequence GC Content_AfterFilter

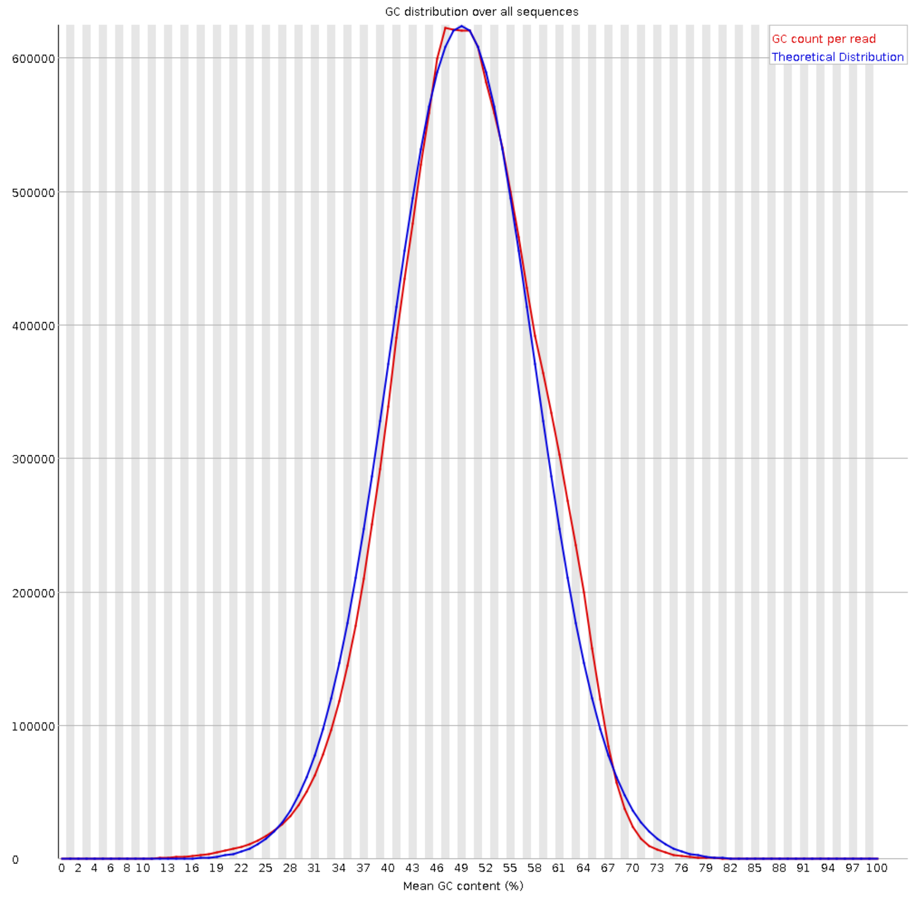

Control 3 Sequence GC Content_Before Filter
