# Supplementary material for: RNA-seq profiles of chicken type II pneumocyte in response to Escherichia coli infection
Source: PLoS One. 2019 Jun 5;14(6):e0217438. doi: 10.1371/journal.pone.0217438 (PMC6550405; doi:10.1371/journal.pone.0217438)
Supplement: S1 Table — (PDF) [file pone.0217438.s005.pdf]

S1. Table List of RT PCR primers used in this study

	Gene/Locus	Forward primer	Reverse primer	References
1	CSF3	F-AACCTCTCCTCCAACATCCAG	R-GTACGCCGTCTCCAGGAAG	Ama Szmolka et al.,2015
2	IL8L2	F-CAAGCCAAACACTCCTAACCAT	R-AGCTCATTCCCCATCTTTACC	Ama Szmolkaet al., 2015
3	IL1β	F-GAAGTGCTTCGTGCTGGAGT	R-ACTGGCATCTGCCCAGTTC	Chranova et al., 2011
4	IL8L1	F-CCTCACTGCAAGAATGTGGA	R-GGAGGAGGTAGGACGTTTTTG	Ama Szmolkaet al., 2015
5	CCL17	F-GCTCCTCAGCATCTTCCAGT	R-CCTTGGTCCCGTTCTTTGT	Ama Szmolkaet al., 2015
6	IL6	F-GCTACAGCACAAAGCACCTG	R-GACTTCAGATTGGCGAGGAG	Berndt et al., 2007
7	ACTB	F-ATGTGGATCAGCAAGCAGGAGTA	R-TTTATGCGCATTTATGGGTTTTGT	GuoA et al., 2011
8	HSPA2	F-TCTCATCAAGCGTAACACCAC	R-TCTCACCTTCATACACCTGGAC	Soheil Varastehal., 2015
9	MYCN	F-ACCACTTTTCCATCGGTCAG	R-TGGCACATCTTCACTCTCCA	this study
10	ATOH8	F-AGCCCAACACATCATCCTTC	R-GCACATCTCACCTGGCACTA	this study
11	NLRP3	F-TCATATCCCACCACCACCTT	R-CACTGACACAGAAGGGCAGA	this study
12	BCL2	F-GTAACAGCACACGCACATCC	R- GCAAGTCCTCAGCCTTTGAC	this study
13	BAX	F- ATTATGGGATGGCGTTATGG	R- GCGGCTTCACTTCTTCTCC	this study
14	NFKBIZ	F- GCCAGAAACTTGGAAAATGAAC	R- TGAACTGTCTTCCCCTTTAGGA	Ama Szmolka et al.,2015
15	NFKBIα	F- TGAGGATGAGGAGAGCAGTG	R- TCTTCCTCCAATACAGCAGTCA	Ama Szmolka et al.,2015
16	TNFIP2	F- TGACCAGATTGCTCCTGAAA	R- TCGTACTTCTGCCATTTTGC	this study
17	HSP90	ATGCCGGAAGCTGTGCAAAC ACAGGACCAA	R-GGAATCAGGTTAATTTTCAG GTCTTTTCCA	Soheil Varastehal., 2015
18	TLR-4	F-CTGACCTACCCATCGGACAC	R-GCCTGAGAGAGGTCAGGTTG	Soheil Varastehal., 2015
