# Supplementary material for: RNA-seq profiles of chicken type II pneumocyte in response to Escherichia coli infection
Source: PLoS One. 2019 Jun 5;14(6):e0217438. doi: 10.1371/journal.pone.0217438 (PMC6550405; doi:10.1371/journal.pone.0217438)
Supplement: S2 Table — (PDF) [file pone.0217438.s006.pdf]

#Item	APEC infection 1
**Statistics**	**Result**
All	13563888
UnMapped	1160813
Mapped	12403075
MappedRate	0.914
UniqueMapped	11725249
UniqueMappedRate	0.864
RepeatMapped	677826
JunctionAllMapped	4154534
JunctionUniqueMapped	4138986
AllBase	2052318050
UnMappedBase	138468360
MappedBase	1913849690
UniqueMappedBase	1815262115
RepeatMappedBase	98587575

#Item	APEC infection 2
**Statistics**	**Result**
All	12557179
UnMapped	1455958
Mapped	11101221
MappedRate	0.884
UniqueMapped	9030741
UniqueMappedRate	0.719
RepeatMapped	2070480
JunctionAllMapped	2716938
JunctionUniqueMapped	2705733
AllBase	1868113503
UnMappedBase	192572016
MappedBase	1675541487
UniqueMappedBase	1381122886
RepeatMappedBase	294418601

#Item	APEC infection 3
**Statistics**	**Result**
All	14314412
UnMapped	1223210
Mapped	13091202
MappedRate	0.915
UniqueMapped	12232180
UniqueMappedRate	0.855
RepeatMapped	859022
JunctionAllMapped	4350915
JunctionUniqueMapped	4333371
AllBase	2181930019
UnMappedBase	149452087
MappedBase	2032477932
UniqueMappedBase	1908905365
RepeatMappedBase	123572567

#Mapping_Statistics

#Item	control 1
**Statistics**	**Result**
All	13294410
UnMapped	1540420
Mapped	11753990
MappedRate	0.884
UniqueMapped	11394098
UniqueMappedRate	0.857
RepeatMapped	359892
JunctionAllMapped	3957894
JunctionUniqueMapped	3948257
AllBase	2201734135
UnMappedBase	198665053
MappedBase	2003069082
UniqueMappedBase	1943811619
RepeatMappedBase	59257463

#Mapping_Statistics

#Item	control 2
**Statistics**	**Result**
All	15609688
UnMapped	1968074
Mapped	13641614
MappedRate	0.874
UniqueMapped	13127738
UniqueMappedRate	0.841
RepeatMapped	513876
JunctionAllMapped	5389519
JunctionUniqueMapped	5376311
AllBase	2273043083
UnMappedBase	238676032
MappedBase	2034367051
UniqueMappedBase	1961761548
RepeatMappedBase	72605503

#Mapping_Statistics

#Item	control 3
**Statistics**	**Result**
All	14984846
UnMapped	1589747
Mapped	13395099
MappedRate	0.894
UniqueMapped	12961296
UniqueMappedRate	0.865
RepeatMapped	433803
JunctionAllMapped	4815163
JunctionUniqueMapped	4792947
AllBase	2304950402
UnMappedBase	195280045
MappedBase	2109670357
UniqueMappedBase	2045128761
RepeatMappedBase	64541596
