# Supplementary material for: RNA-seq profiles of chicken type II pneumocyte in response to Escherichia coli infection
Source: PLoS One. 2019 Jun 5;14(6):e0217438. doi: 10.1371/journal.pone.0217438 (PMC6550405; doi:10.1371/journal.pone.0217438)
Supplement: S4 Table — (PDF) [file pone.0217438.s008.pdf]

S4. Table. Selected data from the RNA-seq of chicken type II pneumocyte, with a focus on transcripts representing Gallus gallus only.

Up- and down-regulated transcripts are listed in descending order of their expression fold change＞1.5，FDR≤0.05.

AccID	APEC infection	Control	Log2FC	FDR	Style	Symbol	Blast_Symbol
IL8L1	8845.43083	39.07138192	7.82267632	0	up	IL8L1	CXCL8
LOC107056355	4191.689772	63.25914553	6.050114067	0	up	LOC107056355	
IL8L2	6268.718468	96.78437973	6.017252503	0	up	IL8L2	CXCL8
PTGS2	7986.603271	281.3781556	4.826999814	0	up	PTGS2	PTGS2
HIST1H2B7L4	164.1059195	8.844069424	4.213773119	0	up	HIST1H2B7L4	HIST2H2BE
CSF3	156.706788	8.635322436	4.181673815	0	up	CSF3	CSF3
CD83	261.9558333	30.43461304	3.105538661	0	up	CD83	CD83
IL1B	284.8163207	34.03146761	3.065090545	0	up	IL1B	IL1B
IRG1	491.6608647	59.1287347	3.055732214	0	up	IRG1	IRG1
HIST1H111R	224.4576926	27.7523191	3.015763302	0	up	HIST1H111R	HIST1H1D
PLAU	958.4568589	129.0663666	2.892600398	0	up	PLAU	PLAU
LOC101750628	104.3140132	14.36165559	2.860639003	0	up	LOC101750628	
ATF3	3587.66925	585.3501156	2.615675186	0	up	ATF3	
TNIP2	344.1113485	58.1283495	2.565561621	0	up	TNIP2	TNIP2
PFKFB3	1848.715453	356.3279432	2.375245659	0	up	PFKFB3	
CHAC1	1663.015607	332.8252115	2.320965081	0	up	CHAC1	CHAC1
NDRG1	9646.523374	2200.053519	2.132470466	0	up	NDRG1	xp_005250810
LOC107054477	143.6941025	33.20828095	2.113385904	0	up	LOC107054477	
INPP5F	940.042039	221.3812475	2.086192255	0	up	INPP5F	INPP5F
TGFBR3	664.9691053	156.94291	2.083047459	0	up	TGFBR3	TGFBR3
RRAD	677.0483267	167.4469225	2.015554954	0	up	RRAD	RRAD
SLC16A6	562.804805	145.0762664	1.955823124	0	up	SLC16A6	xp_005277841
LPP	1341.889608	370.6527568	1.856125845	0	up	LPP	LPP
PLIN2	7206.802293	2024.011611	1.832141702	0	up	PLIN2	PLIN2
BIRC2	2615.024406	788.3227426	1.72996611	0	up	BIRC2	BIRC2
UNC119	961.9160978	291.1281528	1.724256703	0	up	UNC119	UNC119
LOC107054109	144.0716612	43.85115296	1.716099906	0	up	LOC107054109	
ETS2	2658.279264	836.7072461	1.66769784	0	up	ETS2	ETS2
ZC3H12A	2173.96457	688.6732073	1.658436973	0	up	ZC3H12A	ZC3H12A
ENO2	776.0418658	250.8467899	1.629328009	0	up	ENO2	xp_005277807
LOC419429	141.9074301	45.92025901	1.627747445	0	up	LOC419429	PTPN11
CCSER1	424.3254669	138.012097	1.620376541	0	up	CCSER1	CCSER1
LOC101751641	1369.237863	448.6274669	1.609783236	0	up	LOC101751641	
GABRA4	395.2322067	129.5287247	1.609428445	0	up	GABRA4	GABRA4
SLC25A29	620.7000118	203.5920078	1.608215245	0	up	SLC25A29	SLC25A29
TRIM24	354.1795817	116.2897356	1.606757282	0	up	TRIM24	
ZFYVE28	250.2041459	83.4712865	1.583753784	0	up	ZFYVE28	ZFYVE28
PPM1K	533.313242	178.9270605	1.575611557	0	up	PPM1K	PPM1K
SLC25A37	584.9838682	200.518313	1.544662839	0	up	SLC25A37	SLC25A37
DENND5A	1518.518084	523.1406658	1.537393264	0	up	DENND5A	
GFOD1	2128.856894	763.377944	1.479609563	0	up	GFOD1	GFOD1
SLC43A2	589.4255551	211.6148251	1.477868909	0	up	SLC43A2	SLC43A2
LOC378902	393.633368	142.1097165	1.469847322	0	up	LOC378902	TNFRSF10A
BACH2	513.3738462	190.5008504	1.430212362	0	up	BACH2	BACH2
USP49	361.2220083	135.9663138	1.409636533	0	up	USP49	xp_005249048
ABHD2	3888.137231	1485.02592	1.388591027	0	up	ABHD2	ABHD2
IFRD1	5167.021979	1983.087034	1.381585024	0	up	IFRD1	IFRD1
LARP4B	1518.372558	596.0958628	1.348909558	0	up	LARP4B	
PLSCR5	224.4493907	88.55460119	1.341751005	0	up	PLSCR5	PLSCR5

AREG	2703.151919	1075.314599	1.329883792	0	up	AREGB	xp_001125684
PDXP	336.5566229	134.0679246	1.327885129	0	up	PDXP	
DUSP1	3243.277179	1299.035582	1.320011377	0	up	DUSP1	DUSP1
HECW2	748.6239484	302.2844488	1.30833444	0	up	HECW2	
CHMP4C	1071.717963	448.8505432	1.255618246	0	up	CHMP4C	CHMP4C
RC3H1	1207.323697	545.1564081	1.14707042	0	up	RC3H1	
CRIM1	1516.499941	693.3857932	1.129015259	0	up	CRIM1	CRIM1
GABARAPL1	2475.994061	1137.512942	1.122124894	0	up	GABARAPL1	GABARAPL1
FAM110B	324.5762139	150.2204589	1.111475967	0	up	FAM110B	FAM110B
USP40	235.2451538	109.5890973	1.102060727	0	up	USP40	USP40
TIPARP	2541.796196	1197.31566	1.086044804	0	up	TIPARP	TIPARP
MXI1	719.4234335	341.4346945	1.075231489	0	up	MXI1	MXI1
JMY	573.4988082	278.97308	1.039664575	0	up	JMY	JMY
TMPRSS2	244.8578658	120.6765105	1.020799657	0	up	TMPRSS2	TMPRSS2
MITF	807.8592509	401.980516	1.006978386	0	up	MITF	MITF
AP000295.9	650.069698	330.6839555	0.975141358	0	up	AP000295.9	
RASSF3	2703.063114	1399.128853	0.950066364	0	up	RASSF3	RASSF3
FAM13A	3485.834142	1805.241968	0.949311703	0	up	FAM13A	
IPP	455.9157291	237.5652321	0.940443471	0	up	IPP	IPP
MYNN	527.8347247	275.7964724	0.93648226	0	up	MYNN	MYNN
TMEM38A	411.9285334	216.2856262	0.929456271	0	up	TMEM38A	TMEM38A
MAP1LC3B	2506.569357	1328.743505	0.915651514	0	up	MAP1LC3B	MAP1LC3B
OXSR1	939.4723466	504.4574025	0.897118246	0	up	OXSR1	OXSR1
NMD3	598.2678562	331.1783918	0.853183007	0	up	NMD3	NMD3
PKP2	976.9178845	553.9680761	0.818434461	0	up	PKP2	PKP2
ADCK3	2226.094088	1273.098239	0.806170822	0	up	ADCK3	xp_005273263
GJA1	1850.075601	1066.120907	0.795213165	0	up	GJA1	GJA1
C26H6ORF89	775.539252	449.8272073	0.785828878	0	up	C26H6ORF89	C6orf89
PDCD6IP	5509.392147	3249.93676	0.761481509	0	up	PDCD6IP	PDCD6IP
TMF1	1837.21946	1114.16583	0.721559993	0	up	TMF1	xp_005264827
IRF6	3134.21291	1915.794763	0.710160172	0	up	IRF6	xp_005273175
SLC37A3	1723.941836	1066.732703	0.692512383	0	up	SLC37A3	xp_005250115
SRPRB	1885.333212	1181.023811	0.674781475	0	up	SRPRB	xp_005247741
BAG3	3160.678599	1988.655331	0.668441085	0	up	BAG3	
NAV2	772.0189257	491.8857898	0.650312837	0	up	NAV2	NAV2
TMEM131	2430.196526	1559.252824	0.640218115	0	up	TMEM131	TMEM131
TOP1	2678.022303	1719.08965	0.639523193	0	up	TOP1	TOP1
EIF5B	3617.296111	2332.294698	0.633161609	0	up	EIF5B	EIF5B
MAP2K1	1749.241632	1146.706255	0.609233717	0	up	MAP2K1	MAP2K1
ARFGAP2	530.0418369	848.5546181	-0.678901285	0	down	ARFGAP2	ARFGAP2
SIK2	398.0432673	643.5598628	-0.693149091	0	down	SIK1	SIK2
ACSBG2	448.2030508	731.5064469	-0.70671811	0	down	ACSBG2	xp_005259707
LOC101750205	1039.70052	1705.997055	-0.714447129	0	down	LOC101750205	
LOC770305	269.0588818	452.4510502	-0.749839786	0	down	LOC770305	
TFIP11	292.1223983	521.8894184	-0.837171171	0	down	TFIP11	xp_005261535
CSTF1	171.4040896	307.375276	-0.842599593	0	down	CSTF1	CSTF1
SART3	429.6920428	773.7868712	-0.84863319	0	down	SART3	SART3
DNAJA1	736.819991	1330.131223	-0.852184471	0	down	DNAJA1	xp_005251502
ASB1	190.3074303	358.4082732	-0.913272048	0	down	ASB1	ASB1
RANBP3	551.4761586	1054.819083	-0.935625156	0	down	RANBP3	RANBP3
PPP2R5D	736.1267942	1416.699431	-0.944507517	0	down	PPP2R5D	
RIPK4	1022.5984	2043.991758	-0.999149704	0	down	RIPK4	RIPK4
TNFRSF11A	150.3854228	312.277057	-1.054161848	0	down	TNFRSF11A	TNFRSF11A
DROSHA	154.0491184	333.8122502	-1.115646473	0	down	DROSHA	DROSHA

FBXL18	85.48232636	195.8812443	-1.19628119	0	down	FBXL18	FBXL18
HSPA8	14483.30516	33244.20654	-1.198712074	0	down	HSPA8	xp_005271592
MB21D1	80.3720327	185.4151048	-1.205993302	0	down	MB21D1	MB21D1
LOC101749275	130.436692	302.9143249	-1.215560047	0	down	LOC101749275	SPATA2
FLRT3	344.3358316	805.0008086	-1.225173917	0	down	FLRT3	
OGFR	128.6084218	304.4556057	-1.243246757	0	down	OGFR	xp_005260244
LOC101747869	68.5621053	175.0101014	-1.35195488	0	down	LOC101747869	MYPOP
MYOM3	452.3387192	1155.286838	-1.352775695	0	down	MYOM3	
BLM	69.60138881	188.8813844	-1.440292523	0	down	BLM	BLM
F13A1	163.3867146	450.265339	-1.462484745	0	down	F13A1	F13A1
MCM10	52.57360139	146.9556679	-1.482970532	0	down	MCM10	
C2CD3	54.94069805	159.6503255	-1.538968349	0	down	C2CD3	xp_005273951
MYCN	106.1015591	322.3317729	-1.603100549	0	down	MYCN	MYCN
CDCP2	216.0744527	659.9174024	-1.610756956	0	down	CDCP2	CDCP2
PGM2L1	43.52701478	134.9868246	-1.632835616	0	down	PGM2L1	PGM2L1
IL22RA1	252.875541	806.8663041	-1.673902141	0	down	IL22RA1	IL22RA1
CLSPN	45.84346335	153.0050246	-1.738791086	0	down	CLSPN	CLSPN
LOC100857913	30.03154765	114.2684633	-1.927876562	0	down	LOC100857913	NWD1
LOC107057277	36.67354165	139.9508448	-1.932108693	0	down	LOC107057277	
ZBTB7C	42.77874089	255.2642817	-2.577025755	0	down	ZBTB7C	xp_005258288
VDHAP	14.36835939	115.6142406	-3.008351865	0	down	VDHAP	
LOC107054036	23.83834098	196.2047429	-3.041004176	0	down	LOC107054036	
ANKRD29	111.5654505	35.15530919	1.666075836	1.11E-16	up	ANKRD29	ANKRD29
NR4A3	324.7228866	112.8151755	1.525247923	1.11E-16	up	NR4A3	NR4A3
SMURF1	1715.818101	675.3968418	1.345089278	1.11E-16	up	SMURF1	SMURF1
HSP25	1797.327483	362.6323955	2.309273582	2.22E-16	up	HSP25	CRYAB
CREBRF	255.2814553	111.1343739	1.199783626	2.22E-16	up	CREBRF	CREBRF
LONRF3	362.4697292	169.9313975	1.092908079	2.22E-16	up	LONRF3	LONRF3
SRSF5	297.1197405	984.6135707	-1.728513164	2.22E-16	down	SRSF5	SRSF5
AFAP1	1024.176834	625.1878979	0.712103075	3.33E-16	up	AFAP1	xp_005248049
POLR3E	1099.69094	639.4808352	0.782125093	4.44E-16	up	POLR3E	
PBX3	94.77571974	196.9958513	-1.055575835	4.44E-16	down	PBX3	PBX3
KSR1	984.3570343	540.2871804	0.865455237	7.77E-16	up	KSR1	KSR1
CLK1	662.4664565	245.9035301	1.429754961	9.99E-16	up	CLK1	CLK1
CXORF23	627.5054688	262.8779444	1.255234928	9.99E-16	up	CXORF23	
ARRDC3	395.0463931	142.9169066	1.466845497	1.11E-15	up	ARRDC3	ARRDC3
KIAA0195	406.5094181	738.4816185	-0.861273234	1.11E-15	down	KIAA0195	KIAA0195
VSIG4	35.79319408	95.94697806	-1.422552077	1.11E-15	down	VSIG4	VSIG4
B3GNT4	51.83569954	148.6060412	-1.519474829	1.11E-15	down	B3GNT4	B3GNT4
LONP2	635.8012746	334.3565137	0.927188686	1.22E-15	up	LONP2	LONP2
CBX7	76.86977998	13.95782958	2.461341917	1.33E-15	up	CBX7	
UBE2H	1681.924942	786.3189854	1.096926732	1.33E-15	up	UBE2H	UBE2H
TOPORS	145.8706122	311.406389	-1.094109283	1.67E-15	down	TOPORS	TOPORS
WBP4	900.2742307	384.469821	1.227494167	2.33E-15	up	WBP4	WBP4
LOC424892	193.15176	77.37417702	1.319810759	2.89E-15	up	LOC424892	BDH1
NOTUM	544.3779758	869.9626608	-0.676344781	2.89E-15	down	NOTUM	
LOC107052553	89.88507047	256.5258584	-1.512950845	2.89E-15	down	LOC107052553	
CZH18ORF25	567.2777407	310.3706892	0.870062937	3.33E-15	up	CZH18ORF25	C18orf25
RASD1	278.3086341	95.79750954	1.538625611	3.55E-15	up	RASD1	RASD1
DIS3L	698.803714	272.5989427	1.35810731	3.66E-15	up	DIS3L	DIS3L
ILDR2	159.1461118	426.3048814	-1.421533665	3.66E-15	down	ILDR2	ILDR2
TSEN54	134.5316361	404.4151162	-1.58789145	3.66E-15	down	TSEN54	LLGL2
TLE1	120.5806748	262.4469313	-1.122027021	3.77E-15	down	TLE1	TLE1
LOC418249	402.6043471	103.352314	1.961792059	5.11E-15	up	LOC418249	

RYK	1164.786143	415.4277753	1.487395515	5.11E-15	up	RYK	RYK
DHX40	514.4637663	326.4452158	0.656228573	5.11E-15	up	DHX40	DHX40
ZMAT3	438.83015	196.5035573	1.15910722	1.30E-14	up	ZMAT3	ZMAT3
SLC1A4	2262.295403	886.2911085	1.351934779	1.50E-14	up	SLC1A4	SLC1A4
FAM126B	249.5904642	91.07081513	1.454502114	1.53E-14	up	FAM126B	FAM126B
TRPS1	534.1764953	219.3543972	1.284052869	1.84E-14	up	TRPS1	xp_005251103
KLHL26	50.50934474	152.4624242	-1.593831489	1.90E-14	down	KLHL26	
HEG1	102.5522849	235.7635366	-1.200980971	1.97E-14	down	HEG1	HEG1
NCOA5	264.8344844	739.1709261	-1.480817023	2.13E-14	down	NCOA5	NCOA5
LOC107055032	704.9683269	1063.955473	-0.593807428	2.48E-14	down	LOC107055032	
LOC107054120	63.41007723	161.0544819	-1.344764771	2.78E-14	down	LOC107054120	
RNF165	938.9439229	291.8999938	1.685564817	3.20E-14	up	RNF165	RNF165
KIAA1191	700.3054238	432.1209124	0.69654921	4.29E-14	up	KIAA1191	KIAA1191
SSX2IP	108.5297856	35.73876052	1.602529534	4.52E-14	up	SSX2IP	SSX2IP
JADE3	528.4893097	1343.627392	-1.346186919	4.61E-14	down	JADE3	
PPARG	776.0120676	114.077982	2.766058722	5.26E-14	up	PPARG	PPARG
EAF2	324.3222635	99.86215139	1.69941817	5.55E-14	up	EAF2	EAF2
KBTBD4	179.5570668	494.6662745	-1.462013108	6.48E-14	down	KBTBD4	KBTBD4
ORAI2	607.1125873	398.9705616	0.605681785	7.05E-14	up	ORAI2	ORAI2
CRB2	2303.4585	4320.563343	-0.907417827	9.38E-14	down	CRB2	CRB2
MCTP2	216.8456236	77.51315498	1.484155246	9.84E-14	up	MCTP2	
INHBA	2326.486246	852.5532891	1.44829074	1.04E-13	up	INHBA	INHBA
LOC107054666	31.32010636	90.39240112	-1.529112385	1.68E-13	down	LOC107054666	
RGS1	121.0202483	6.786624788	4.156410386	1.76E-13	up	RGS1	RGS1
E4F1	133.542564	303.5572755	-1.184669107	2.18E-13	down	E4F1	
ODC1	2638.887968	1019.487307	1.37208629	3.03E-13	up	ODC1	xp_005246222
CGRRF1	326.4123492	92.21708012	1.823589749	3.05E-13	up	CGRRF1	CGRRF1
MYO1F	16.19720292	73.57030642	-2.183378903	3.26E-13	down	MYO1F	MYO1F
RNF126	1848.449509	980.8298872	0.914240791	3.35E-13	up	RNF126	RNF126
ERCC6L	66.92796208	168.6525641	-1.333373262	4.10E-13	down	ERCC6L	ERCC6L
LOC107051736	30.33438766	114.7958136	-1.920043936	4.53E-13	down	LOC107051736	
AATF	275.4019626	469.8928363	-0.770792933	4.70E-13	down	AATF	AATF
LOC107054808	87.6194066	300.250649	-1.776845015	5.39E-13	down	LOC107054808	
LOC107052041	66.10934458	17.68737516	1.902134246	5.97E-13	up	LOC107052041	
SDC4	2833.099104	1674.736611	0.758446851	6.16E-13	up	SDC4	SDC4
BCL2	130.5100889	47.71574848	1.451623929	8.00E-13	up	BCL2	BCL2
LOC420486	143.2931831	41.75292525	1.779020795	8.28E-13	up	LOC420486	FASN
MBNL2	926.06071	471.1525636	0.974912482	8.89E-13	up	MBNL2	MBNL2
RASGRP1	993.5124897	332.6089586	1.578711077	9.73E-13	up	RASGRP1	RASGRP1
LOC420294	144.7920628	377.7382841	-1.383404491	1.05E-12	down	LOC420294	CYLD
TMEM104	192.53692	454.8101979	-1.240129486	1.11E-12	down	TMEM104	xp_005257536
L3MBTL1	243.6794939	757.863446	-1.636953072	1.31E-12	down	L3MBTL1	xp_005260445
TGFB2	640.5905296	307.2127717	1.060164275	1.57E-12	up	TGFB2	TGFB2
CREB5	393.5268348	164.4988983	1.258384095	1.59E-12	up	CREB5	CREB5
LOC107055306	173.420915	55.58544484	1.641498837	1.60E-12	up	LOC107055306	
TICAM1	189.9360052	362.8806649	-0.933981773	1.70E-12	down	TICAM1	TICAM1
CSNK1D	2176.102155	1135.028128	0.939018234	1.79E-12	up	CSNK1D	CSNK1D
CPNE8	406.0361196	80.91692401	2.327094687	1.81E-12	up	CPNE8	CPNE8
SLC22A23	323.2552443	141.0176311	1.196798225	2.27E-12	up	SLC22A23	SLC22A23
RIPK3	243.783703	94.9573418	1.36025023	2.68E-12	up	RIPK3	
KIAA1551	874.6146612	304.2122727	1.523569176	3.42E-12	up	KIAA1551	
DHRSX	969.4749849	585.7777239	0.726850342	4.28E-12	up	DHRSX	
CHST12	185.8769621	342.3894102	-0.88129011	5.05E-12	down	CHST12	CHST12
FAM214A	1542.17403	630.0947166	1.291324961	5.16E-12	up	FAM214A	xp_005254603

FAM102A	309.3140701	162.4472484	0.929101153	5.60E-12	up	FAM102A	FAM102A
MIRLET7D	12.31898645	59.83212155	-2.280036657	5.73E-12	down	MIRLET7D	
HIF1A	3605.075991	1680.261334	1.101344035	5.94E-12	up	HIF1A	HIF1A
HOMER3	247.7540172	517.2624551	-1.061988029	6.06E-12	down	HOMER3	xp_005251712
B4GALT4	1023.441981	476.0712364	1.104179948	6.77E-12	up	B4GALT4	xp_005247913
PRDM11	123.3879452	421.4570347	-1.772184111	7.63E-12	down	PRDM11	PRDM11
UBQLN1	1866.748318	1064.660908	0.810133423	8.09E-12	up	UBQLN1	UBQLN1
CHUK	396.9037362	828.7908149	-1.062218871	9.75E-12	down	CHUK	CHUK
STK38L	2893.378351	1041.792816	1.473686595	9.88E-12	up	STK38L	STK38L
CLDN5	134.3195837	329.1557278	-1.293100639	1.02E-11	down	CLDN5	CLDN5
PTP4A3	390.9753714	195.5128948	0.999813969	1.08E-11	up	PTP4A3	xp_005250822
MFI2	38.6315537	130.2723475	-1.753679274	1.31E-11	down	MFI2	MFI2
EIF3F	1462.195108	974.4430837	0.585486003	1.57E-11	up	EIF3F	EIF3F
CYSLTR1	36.89129094	113.8353799	-1.625596836	1.65E-11	down	CYSLTR1	xp_005262118
ENTPD4	732.35655	274.9288878	1.413487696	1.92E-11	up	ENTPD4	ENTPD4
ADD3	1648.509627	1002.910809	0.716969002	2.03E-11	up	ADD3	ADD3
LOC422171	980.067713	1545.215439	-0.656854664	2.11E-11	down	LOC422171	ARHGAP20
RSBN1	264.031499	426.57857	-0.692101437	2.35E-11	down	RSBN1	
FAM53B	74.82945732	203.84886	-1.445821671	2.43E-11	down	FAM53B	xp_005270357
AFF4	3336.98208	1756.051153	0.926209068	2.56E-11	up	AFF4	AFF4
TNFAIP3	668.2612546	342.7713729	0.963165604	2.77E-11	up	TNFAIP3	
LOC101750431	25.09435215	2.340024393	3.422767228	3.22E-11	up	LOC101750431	
TMEM186	113.2109874	201.5342197	-0.832010842	4.08E-11	down	TMEM186	
CTH	486.8496478	146.7125515	1.730483997	4.86E-11	up	CTH	CTH
RGS9	170.0253436	69.97890725	1.280757766	5.78E-11	up	RGS9	
CMIP	1404.167044	804.063302	0.804333582	6.26E-11	up	CMIP	CMIP
LOC101749989	3.333693176	49.13619015	-3.881592766	6.32E-11	down	LOC101749989	
PMAIP1	52.98516598	15.15247532	1.806035019	6.40E-11	up	PMAIP1	
TRADD	248.853709	483.1435588	-0.957154038	7.46E-11	down	TRADD	TRADD
TFCP2L1	122.35159	43.17253537	1.502847124	7.71E-11	up	TFCP2L1	TFCP2L1
EPHA1	178.2705398	306.9284699	-0.783834163	7.75E-11	down	EPHA1	EPHA1
ITPRIPL2	272.074578	613.8448164	-1.173871819	8.23E-11	down	ITPRIPL2	ITPRIPL2
LOC417447	531.8271875	322.1994212	0.723003629	1.36E-10	up	LOC417447	
C3ORF70	77.97706372	206.3999772	-1.404321076	1.74E-10	down	C3ORF70	
KCNQ1	573.3130292	131.1416281	2.128197356	2.04E-10	up	KCNQ1	KCNQ1
TESK2	79.16534786	18.93874994	2.063527966	2.09E-10	up	TESK2	TESK2
PRIMPOL	125.7290492	28.08989694	2.162194781	2.12E-10	up	PRIMPOL	
PIGQ	103.9101772	184.6858284	-0.829736206	2.19E-10	down	PIGQ	
ENTPD2L	91.41517586	196.8149831	-1.106334462	2.32E-10	down	ENTPD2L	
REL	881.152196	518.7068125	0.764471911	2.34E-10	up	REL	REL
LOC101751133	76.48228598	145.4094918	-0.926923896	3.01E-10	down	LOC101751133	
PIK3AP1	451.2606411	249.2882019	0.856146347	3.29E-10	up	PIK3AP1	PIK3AP1
SPTY2D1	572.2788731	350.8933524	0.705685731	3.44E-10	up	SPTY2D1	SPTY2D1
TADA2A	60.92141552	158.7731759	-1.381945827	3.71E-10	down	TADA2A	xp_005257684
RFFL	1027.738987	579.2273987	0.82727216	3.74E-10	up	RFFL	RFFL
FJX1	103.1670268	250.9262138	-1.28228125	4.65E-10	down	FJX1	
JMJD6	441.7297798	1117.742599	-1.339351991	4.77E-10	down	JMJD6	JMJD6
GOPC	808.873142	501.0134235	0.691064201	5.12E-10	up	GOPC	
HESX1	68.04107926	23.15810796	1.554888637	5.45E-10	up	HESX1	HESX1
MMP16	324.3167033	85.06037484	1.930844211	5.54E-10	up	MMP16	MMP16
KIF16B	465.3331406	201.1589725	1.209927848	5.87E-10	up	KIF16B	KIF16B
PPARA	326.9834231	85.28312909	1.938885221	6.60E-10	up	PPARA	xp_005261715
LOC107053805	15.67939373	77.60617897	-2.307301747	7.29E-10	down	LOC107053805	
BICD2	4710.734459	2857.810872	0.721041567	7.72E-10	up	BICD2	BICD2

ZNF592	432.8145685	662.9755209	-0.615206541	8.37E-10	down	ZNF592	ZNF592
LOC101751989	35.34281179	85.34392186	-1.271871587	8.50E-10	down	LOC101751989	
APCDD1	341.3267057	585.060275	-0.777431968	8.51E-10	down	APCDD1	xp_005258140
ULK1	93.47037068	373.4219193	-1.998225594	8.85E-10	down	ULK1	ULK1
SEPT11	3390.924417	2059.384999	0.719465062	8.90E-10	up	SEPT11	SEPT11
LOC107054677	1236.55921	588.6242401	1.070912462	1.02E-09	up	LOC107054677	
ELK4	499.8373546	251.2714066	0.992212214	1.10E-09	up	ELK4	ELK4
HOOK1	442.1477743	170.3097584	1.376367526	1.19E-09	up	HOOK1	HOOK1
SLC22A5	242.0116295	75.01710369	1.689784907	1.41E-09	up	SLC22A5	SLC22A5
RP11-101E3.5	572.8913851	899.0513869	-0.650141935	1.45E-09	down	RP11-101E3.5	
PELI1	935.8207931	614.2991524	0.607290892	1.52E-09	up	PELI1	PELI1
ASB7	164.4474522	73.13328193	1.169026645	1.69E-09	up	ASB7	ASB7
MAGI3	436.0621378	835.540569	-0.938176149	1.71E-09	down	MAGI3	MAGI3
PRDM10	416.1260954	822.9392919	-0.983765244	1.79E-09	down	PRDM10	PRDM10
RRAGB	1152.074141	736.1838106	0.646095634	1.83E-09	up	RRAGB	
CCL20	147.6785482	1.917770851	6.266886118	1.94E-09	up	CCL20	CCL20
HORMAD2	832.7981186	1284.419088	-0.625077295	2.16E-09	down	HORMAD2	xp_005261421
TPRA1	288.7279851	463.242246	-0.682055879	2.27E-09	down	TPRA1	TPRA1
GTF2I	227.8236601	369.2101876	-0.696524779	2.31E-09	down	GTF2I	
ATXN1L	600.5851821	1061.827156	-0.822108158	2.54E-09	down	ATXN1L	
KCMF1	1093.766377	453.7050072	1.269478131	2.68E-09	up	KCMF1	KCMF1
LRRC8D	452.3161185	201.4468589	1.166932098	3.76E-09	up	LRRC8D	LRRC8D
NINJ1	331.6544164	127.3655553	1.380705569	4.07E-09	up	NINJ1	NINJ1
IL17RD	143.2771528	49.28047471	1.539720515	5.29E-09	up	IL17RD	IL17RD
FDXACB1	14.06260266	46.90284464	-1.737811796	5.44E-09	down	FDXACB1	FDXACB1
WTIP	566.6192443	216.4799588	1.388146131	5.71E-09	up	WTIP	WTIP
DSTYK	154.5888184	308.1406438	-0.995153016	5.73E-09	down	DSTYK	DSTYK
NFKBIA	4104.475146	1469.219962	1.482147348	5.95E-09	up	NFKBIA	NFKBIA
GRM7	44.86255646	163.2432521	-1.863439618	6.10E-09	down	GRM7	GRM7
XKR6	137.4292304	57.16169676	1.265568244	7.26E-09	up	XKR6	
GLUD1	868.3594782	441.7976411	0.974906689	7.75E-09	up	GLUD1	
TRAF3	1022.367507	359.3630389	1.508399952	8.10E-09	up	TRAF3	TRAF3
TFPI2	211.8687253	121.4518087	0.802786667	8.20E-09	up	TFPI2	TFPI2
CSF2	68.24991487	2.679333189	4.670881343	9.83E-09	up	CSF2	
PHF20	524.7752893	873.367537	-0.73488912	1.02E-08	down	PHF20	PHF20
SENP2	372.3244246	216.5851192	0.781626137	1.03E-08	up	SENP2	SENP2
MYLK3	178.4741538	83.22175644	1.100682519	1.12E-08	up	MYLK3	MYLK3
MMACHC	87.90166607	35.01556755	1.327894039	1.14E-08	up	MMACHC	MMACHC
CENPO	76.34437886	170.5997959	-1.160022079	1.19E-08	down	CENPO	CENPO
ITPR2	1319.699927	805.9041912	0.711529686	1.23E-08	up	ITPR2	
VAPA	942.1562454	476.8706032	0.982368483	1.33E-08	up	VAPA	VAPA
TTLL9	184.7991456	68.26469943	1.436746447	1.41E-08	up	TTLL9	
PPP1R3C	47.43645703	116.0187494	-1.290289808	1.45E-08	down	PPP1R3C	PPP1R3C
METRNL	500.7734584	258.2930447	0.955149305	1.47E-08	up	METRNL	
LOC107054121	20.81945481	74.24340076	-1.834330506	1.53E-08	down	LOC107054121	
OTUD4	297.0467803	491.2031633	-0.725629701	1.84E-08	down	OTUD4	OTUD4
TBC1D20	197.2494842	677.5612503	-1.780329841	1.85E-08	down	TBC1D20	
SPG20	805.2416938	423.7349337	0.926259801	1.98E-08	up	SPG20	SPG20
PLEK2	478.9609931	1000.112552	-1.062182297	2.03E-08	down	PLEK2	PLEK2
MOB2	2015.04891	983.1028099	1.035400654	2.07E-08	up	MOB2	MOB2
MLLT3	431.1385158	204.3357706	1.07720967	2.17E-08	up	MLLT3	MLLT3
AR	57.60202117	197.0399475	-1.774296809	2.18E-08	down	AR	AR
CDCP1	1918.810771	1137.071874	0.754888994	2.25E-08	up	CDCP1	CDCP1
ARID4B	610.7864914	297.7473738	1.036579371	2.36E-08	up	ARID4B	ARID4B

KIAA1467	258.569924	141.7436997	0.867269864	2.38E-08	up	KIAA1467	FAM234B
WBP2	1057.296078	470.9700062	1.166672346	2.42E-08	up	WBP2	WBP2
PLCXD2	58.85759562	8.626006882	2.770463836	2.47E-08	up	PLCXD2	PLCXD2
LOC107054622	45.7674229	13.72824135	1.737174237	2.66E-08	up	LOC107054622	
ARHGAP6	181.8021502	108.2555414	0.747928386	3.10E-08	up	ARHGAP6	ARHGAP6
RFX3	305.6784219	109.8313183	1.476725218	3.30E-08	up	RFX3	RFX3
C1H7ORF60	363.1746198	102.541281	1.824458559	3.38E-08	up	C1H7ORF60	C7orf60
SPTAN1	4737.91792	7270.463342	-0.617794104	3.49E-08	down	SPTAN1	xp_005252202
LRRC45	96.41745488	293.3555596	-1.605284081	4.25E-08	down	LRRC45	LRRC45
FAM84A	93.05064083	210.889503	-1.180399296	4.30E-08	down	FAM84A	FAM84A
LOC425462	40.05612443	151.9289358	-1.923301918	4.44E-08	down	LOC425462	PHRF1
TRMT10B	96.98198537	178.8825529	-0.88322399	4.74E-08	down	TRMT10B	TRMT10B
KCTD12	129.8816601	265.6312044	-1.032226904	4.82E-08	down	KCTD12	KCTD12
ELMO1	1264.88357	629.3581271	1.007051494	5.06E-08	up	ELMO1	ELMO1
LOC107053487	85.68913027	30.76246401	1.477941144	5.51E-08	up	LOC107053487	
FNIP2	1153.888039	488.6351787	1.239673609	5.63E-08	up	FNIP2	FNIP2
PPP1R13B	1975.837147	1117.451355	0.822252012	5.70E-08	up	PPP1R13B	xp_005267543
VGLL4	1854.770436	1096.510891	0.758320494	5.84E-08	up	VGLL4	VGLL4
LRRN4	5282.037401	9568.014698	-0.857125089	6.22E-08	down	LRRN4	LRRN4
NFKBIZ	870.3096174	440.7628104	0.981526238	6.38E-08	up	NFKBIZ	NFKBIZ
ATP1B1	832.6850806	555.1068416	0.585005501	6.42E-08	up	ATP1B1	ATP1B1
FAM98A	455.4435811	835.3650884	-0.875134503	6.51E-08	down	FAM98A	FAM98A
NIPAL3	2238.240463	932.7463685	1.262808296	6.54E-08	up	NIPAL3	xp_005246003
PRRG1	421.8582323	133.9740012	1.654805194	7.06E-08	up	PRRG1	PRRG1
LOC101750112	719.9291276	293.2300919	1.295821726	7.26E-08	up	LOC101750112	
TGM6L	302.5471576	130.0220407	1.218403828	7.44E-08	up	TGM6L	
EPHA7	50.15382494	14.63340549	1.777094178	7.45E-08	up	EPHA7	xp_005248727
RIOK3	3134.007806	1112.379479	1.494359738	8.34E-08	up	RIOK3	RIOK3
TSHZ2	334.6060393	187.9836144	0.83185657	8.66E-08	up	TSHZ2	TSHZ2
LRRC58	773.565958	437.0696188	0.82366121	8.73E-08	up	LRRC58	
FGF14	77.08709253	22.85716853	1.753842617	9.36E-08	up	FGF14	FGF14
PDZD11	672.8862827	441.9498454	0.606480056	9.54E-08	up	PDZD11	xp_005262329
UHRF1BP1L	877.2625205	334.8778526	1.389373668	1.01E-07	up	UHRF1BP1L	UHRF1BP1L
FAM199X	556.9371718	221.3018915	1.331498804	1.04E-07	up	FAM199X	FAM199X
REPS1	1487.621759	984.4049589	0.595683925	1.09E-07	up	REPS1	REPS1
CD81	1555.488588	976.0140421	0.672394001	1.11E-07	up	CD81	CD81
SEMA3C	5918.810959	3168.358954	0.901571587	1.14E-07	up	SEMA3C	SEMA3C
RECQL5	99.29070541	191.1055501	-0.944639099	1.19E-07	down	RECQL5	RECQL5
HIST1H2BO	168.2789473	63.13410698	1.414363189	1.24E-07	up	HIST1H2B8	HIST2H2BE
TET2	2179.073519	1266.671813	0.782672092	1.24E-07	up	TET2	TET2
JAK1	3301.732361	2027.314035	0.703653597	1.27E-07	up	JAK1	JAK1
LANCL1	237.2017075	534.0480774	-1.170855231	1.33E-07	down	LANCL1	LANCL1
TRAK2	284.5114759	163.9256489	0.79544524	1.36E-07	up	TRAK2	xp_005246853
LOC100858624	15.72872007	58.68509287	-1.899592802	1.39E-07	down	LOC100858624	KCNE5
LOC107052462	68.4926467	245.3717857	-1.840948353	1.48E-07	down	LOC107052462	
ZNF423	134.0936691	270.5932728	-1.012884847	1.49E-07	down	ZNF423	xp_005255911
IL13RA1	514.4083179	212.2330613	1.277264557	1.69E-07	up	IL13RA1	IL13RA1
ZBTB5	238.6954636	432.7562915	-0.858383646	1.85E-07	down	ZBTB5	ZBTB5
K123	147.6165334	71.14201761	1.05308052	1.85E-07	up	K123	ENDOD1
LOC107049951	0.929237176	31.12710218	-5.06598059	2.00E-07	down	LOC107049951	
LOC431205	57.85669307	169.7560526	-1.552907244	2.13E-07	down	LOC431205	
RRP1B	1394.270104	651.8231613	1.096957552	2.23E-07	up	RRP1B	RRP1B
EFCAB14	375.0439609	218.6780951	0.778250998	2.55E-07	up	EFCAB14	EFCAB14
EIF4E3	95.61606446	43.67538781	1.130432512	2.60E-07	up	EIF4E3	EIF4E3

HIST1H2B7L2	746.0858757	240.6446669	1.632437246	2.62E-07	up	HIST1H2B7L2	HIST2H2BE
ASAP1	360.9873552	206.3533316	0.806831572	2.93E-07	up	ASAP1	ASAP1
RIN2	1805.525342	1189.574623	0.601972894	2.99E-07	up	RIN2	RIN2
FAM171B	115.298168	31.70117009	1.862761593	3.12E-07	up	FAM171B	FAM171B
LOC101752215	4.467254289	25.38606593	-2.506576554	3.37E-07	down	LOC101752215	
RAB3A	690.1147059	384.6049608	0.843458805	3.41E-07	up	RAB3A	
C4H4orf32	149.8867358	235.6581685	-0.652822971	3.53E-07	down	C4H4ORF32	
MICAL2	1679.941157	691.7408836	1.28010707	3.64E-07	up	MICAL2	xp_005253302
LOC423919	1471.484759	719.0054719	1.033197944	3.74E-07	up	LOC423919	SHTN1
LOC417131	20.68357939	97.97599686	-2.243942474	3.98E-07	down	LOC417131	
KIAA1958	200.5722321	308.5184576	-0.621234916	4.18E-07	down	KIAA1958	xp_005251796
PKDCCb	80.02586332	191.7743599	-1.260871605	4.22E-07	down	PKDCCb	
XRCC3	235.2918366	423.6991226	-0.848588874	4.60E-07	down	XRCC3	xp_005268104
RASGEF1B	889.410529	358.9801596	1.308945373	4.83E-07	up	RASGEF1B	RASGEF1B
CDCA7	108.3451571	212.8877239	-0.974458091	4.84E-07	down	CDCA7	xp_005246940
DARS2	114.9546036	213.4200373	-0.892631389	4.85E-07	down	DARS2	DARS2
H6PD	578.4719316	357.3179735	0.695038474	4.97E-07	up	H6PD	H6PD
ABHD17C	480.3231392	283.813957	0.759059773	5.08E-07	up	ABHD17C	ABHD17C
LOC107055786	1.894375337	32.62952133	-4.106383715	5.11E-07	down	LOC107055786	
LDHA	39604.29731	18708.63121	1.08195297	5.21E-07	up	LDHA	LDHA
SLX4	247.3420159	608.7342843	-1.299305291	5.45E-07	down	SLX4	SLX4
LOC101748464	19.15042068	77.37444846	-2.014481136	5.84E-07	down	LOC101748464	
ABCC1	5397.265447	2337.441892	1.207298143	5.98E-07	up	ABCC1	ABCC1
POLR1B	262.168513	604.4486102	-1.205125263	6.25E-07	down	POLR1B	
TRA2A	2043.435384	1229.954269	0.732391948	6.46E-07	up	TRA2A	TRA2A
BPI	92.6287042	396.4678757	-2.097672738	6.85E-07	down	BPI	BPI
GPR157	101.1741238	523.8283411	-2.372253762	7.02E-07	down	GPR157	GPR157
SRPK2	612.4142009	345.396242	0.826255351	7.11E-07	up	SRPK2	
GOLGA4	610.6824036	363.6405266	0.747909283	7.14E-07	up	GOLGA4	
ITPK1	814.5441413	521.9985961	0.641946956	7.23E-07	up	ITPK1	xp_005267644
BTBD10	1243.378281	633.2027807	0.973525788	7.28E-07	up	BTBD10	BTBD10
DSP	4183.588331	2497.078438	0.744499757	7.65E-07	up	DSP	DSP
ADPRHL2	309.1414086	510.2323444	-0.722887444	7.75E-07	down	ADPRHL2	ADPRHL2
TLE4	45.27632782	146.9596637	-1.698591374	7.94E-07	down	TLE4	TLE4
GABRG1	36.43287999	6.638399139	2.456333763	8.07E-07	up	GABRG1	GABRG1
DAB2IP	1055.130236	2755.973982	-1.385141184	8.41E-07	down	DAB2IP	DAB2IP
TXNDC15	334.8065999	220.3676015	0.603415833	8.44E-07	up	TXNDC15	xp_005272138
LRRK1	67.39410156	150.2037624	-1.156226715	8.77E-07	down	LRRK1	LRRK1
DSC2	1437.683523	593.7904442	1.275720349	8.89E-07	up	DSC2	DSC2
KLHL11	519.3866514	331.207242	0.649074718	8.92E-07	up	KLHL11	KLHL11
MIA3	653.5563494	1232.590891	-0.9153105	9.20E-07	down	MIA3	MIA3
VLDLR	115.0109485	229.6883625	-0.997906556	9.51E-07	down	VLDLR	VLDLR
SRSF3	2153.751629	3498.402819	-0.699844529	9.57E-07	down	SRSF3	SRSF3
ZNF106	491.6109689	224.8144775	1.128782161	9.70E-07	up	ZNF106	ZNF106
UBE2E2	427.6570033	170.4619799	1.327004171	9.91E-07	up	UBE2E2	
TOLLIP	947.0586327	314.6987238	1.589482418	1.01E-06	up	TOLLIP	TOLLIP
TM7SF3	520.6001759	258.4423775	1.010333147	1.09E-06	up	TM7SF3	TM7SF3
NKX2-1	157.1561371	1028.470096	-2.710229329	1.12E-06	down	NKX2-1	xp_005268088
LOC107056213	40.7260145	11.4328602	1.832764265	1.19E-06	up	LOC107056213	
SLC22A15	30.90532492	5.533667846	2.481547478	1.21E-06	up	SLC22A15	SLC22A15
CD44	7625.456104	4665.303737	0.708852621	1.21E-06	up	CD44	CD44
RBMX	1257.418595	2078.539391	-0.725105086	1.27E-06	down	RBMX	RBMX
LOC107054784	9.827844768	43.19565461	-2.135939212	1.30E-06	down	LOC107054784	
RPS6KA5	502.5199913	173.6369244	1.533107217	1.36E-06	up	RPS6KA5	RPS6KA5

SGPL1	1692.129165	919.7118133	0.879585921	1.44E-06	up	SGPL1	SGPL1
EREG	422.1899778	76.03778308	2.473103956	1.47E-06	up	EREG	EREG
ACOT4	17.99584229	51.68185199	-1.52199414	1.51E-06	down	ACOT4	ACOT2
SFPQ	2157.131108	4240.553964	-0.975138879	1.51E-06	down	SFPQ	SFPQ
SMARCA1	483.7873938	202.1561803	1.258902867	1.56E-06	up	SMARCA1	xp_005262517
LOC431656	599.958075	1149.528457	-0.938108587	1.59E-06	down	LOC431656	ATP8B2
PLAG1	161.3792127	44.58339005	1.855876531	1.64E-06	up	PLAG1	PLAG1
ZNFX1	118.0856063	232.6578731	-0.978376886	1.66E-06	down	ZNFX1	
IGF2R	1741.250097	703.5380165	1.307423145	1.74E-06	up	IGF2R	IGF2R
SHPK	286.9080553	607.0255867	-1.081168855	1.75E-06	down	SHPK	SHPK
NR3C2	586.4477319	344.5911697	0.767116799	1.88E-06	up	NR3C2	NR3C2
CLMP	790.0352504	1820.567049	-1.204398943	1.94E-06	down	CLMP	CLMP
LETM2	444.7190467	281.7327234	0.658567051	1.94E-06	up	LETM2	LETM2
NOS2	268.7286062	115.3776018	1.219786723	2.02E-06	up	NOS2	NOS2
LOC107054755	4.977334953	25.66713138	-2.366476683	2.05E-06	down	LOC107054755	
RPAIN	198.8614751	102.7282153	0.952931327	2.07E-06	up	RPAIN	RPAIN
PPP2R2B	605.521052	276.9035585	1.128793525	2.08E-06	up	PPP2R2B	PPP2R2B
GRHL1	1644.494767	1082.072846	0.603846792	2.09E-06	up	GRHL1	
ACER2	217.5438227	60.16272969	1.85436412	2.09E-06	up	ACER2	ACER2
PMP22	2673.896151	1705.806599	0.648489349	2.14E-06	up	PMP22	PMP22
SOAT1	1130.051441	555.5252809	1.024463975	2.29E-06	up	SOAT1	SOAT1
ERCC6L2	268.1792442	100.7986473	1.411721305	2.33E-06	up	CZH9ORF102	ERCC6L2
HBAA	9.519307928	89.94047886	-3.24004197	2.45E-06	down	HBAA	HBA1
TRPC6	351.6061025	139.6708865	1.331928779	2.49E-06	up	TRPC6	TRPC6
CAB39	2039.484017	911.8555813	1.161326946	2.49E-06	up	CAB39	CAB39
LOC107056043	39.72921383	6.795736766	2.54749837	2.57E-06	up	LOC107056043	
COX11	214.7803453	99.74704329	1.106515995	2.82E-06	up	COX11	COX11
PID1	721.2147388	333.4878481	1.112794688	2.83E-06	up	PID1	PID1
ENDOD1	1441.079729	2644.241901	-0.875704007	2.88E-06	down	ENDOD1	ENDOD1
LOC107051649	176.2349039	68.07320913	1.372340655	2.91E-06	up	LOC107051649	
GLE1	278.2472042	445.6144755	-0.679428907	3.01E-06	down	GLE1	GLE1
LOC107051007	0	33.58706209	-20	3.01E-06	down	LOC107051007	
GAN	584.7437799	237.827548	1.297888776	3.13E-06	up	GAN	GAN
AHRR	480.7272215	178.0487572	1.432946133	3.19E-06	up	AHRR	AHRR
ISM1	384.741779	694.6672355	-0.852431553	3.24E-06	down	ISM1	ISM1
PQLC1	526.5070636	947.4313475	-0.84756852	3.24E-06	down	PQLC1	PQLC1
MKS1	186.1323708	357.4684468	-0.941486923	3.32E-06	down	MKS1	MKS1
FOS	997.2288997	329.9278705	1.595774039	3.41E-06	up	FOS	FOS
MAP3K7CL	370.5203347	134.3757659	1.463279744	3.48E-06	up	MAP3K7CL	
RAB31	1021.326218	568.1804073	0.846022758	3.65E-06	up	RAB31	
WBSCR16	111.6471878	247.7123023	-1.149718607	3.67E-06	down	WBSCR16	WBSCR16
LOC428586	1034.702452	423.7242985	1.288018186	3.68E-06	up	LOC428586	
SPECC1L	3532.308807	2149.155221	0.71684179	3.87E-06	up	SPECC1L	SPECC1L
GALNT18	427.3627653	189.5138183	1.17315817	3.97E-06	up	GALNT18	GALNT18
LOC422320	298.6116015	450.8545302	-0.594391801	4.01E-06	down	LOC422320	LNX2
ADPGK	293.3629524	466.3397935	-0.668694852	4.03E-06	down	ADPGK	ADPGK
PDE7B	250.0633221	166.2418713	0.589009667	4.11E-06	up	PDE7B	
LOC100859041	52.47071024	20.97776704	1.322651199	4.29E-06	up	LOC100859041	ANKRD13C
SLC45A3	170.3070298	430.2509204	-1.337040292	4.33E-06	down	SLC45A3	SLC45A3
SNAP47	338.9091091	162.118485	1.063849815	4.50E-06	up	SNAP47	SNAP47
TSPAN5	266.4782859	75.78942803	1.813949455	4.55E-06	up	TSPAN5	TSPAN17
CASP3	1407.272987	416.8755399	1.755213585	4.58E-06	up	CASP3	CASP3
PRKCD	946.0596021	421.842775	1.165225684	4.61E-06	up	PRKCD	PRKCD
ATP6V1A	1979.187348	1219.307843	0.698845769	4.62E-06	up	ATP6V1A	ATP6V1A

ASB12	15.0756088	134.9172722	-3.161786886	4.85E-06	down	ASB12	ASB12
MTHFD2	992.90736	561.0846483	0.823440678	4.93E-06	up	MTHFD2	MTHFD2
HSPA2	16214.61386	7666.061456	1.080737199	5.08E-06	up	HSPA2	HSPA2
CCSER2	473.3960787	179.6301931	1.39801789	5.36E-06	up	CCSER2	CCSER2
SMC3	1416.943401	831.2832613	0.769370065	5.50E-06	up	SMC3	SMC3
LMO4	742.6260269	449.714432	0.723626695	5.79E-06	up	LMO4	LMO4
ESCO1	741.1039117	466.8715354	0.666650208	5.93E-06	up	ESCO1	ESCO1
HCN2	35.253346	11.91191063	1.565355359	5.98E-06	up	HCN2	HCN2
LCP1	91.07992491	203.5971876	-1.160512626	6.64E-06	down	LCP1	LCP1
SLK	2506.303901	1540.444195	0.702214939	6.71E-06	up	SLK	SLK
DNM1L	3102.0921	1952.603072	0.667842814	7.23E-06	up	DNM1L	DNM1L
NABP1	390.2923677	931.9124549	-1.255639182	7.95E-06	down	NABP1	NABP1
NLGN4X	105.4840487	52.80031911	0.998406297	8.02E-06	up	NLGN4X	NLGN4X
ATOH8	139.7626214	318.5550722	-1.188564237	8.12E-06	down	ATOH8	ATOH8
INPP5D	12.95151718	40.94313095	-1.660500321	8.35E-06	down	INPP5D	INPPL1
PLXNC1	123.7032005	254.3387008	-1.039868176	8.35E-06	down	PLXNC1	
PHTF2	549.0689456	223.8755679	1.294290225	8.91E-06	up	PHTF2	PHTF2
SLC25A38	116.1785155	196.2089596	-0.75604762	9.07E-06	down	SLC25A38	SLC25A38
LOC107055870	0.929237176	14.62657151	-3.976400956	9.09E-06	down	LOC107055870	
LOC107050667	127.8200084	509.6062481	-1.995269278	9.23E-06	down	LOC107050667	
TNFAIP8L3	38.73298649	13.10179681	1.563798064	9.48E-06	up	TNFAIP8L3	TNFAIP8L3
DENND1A	156.5719226	91.86556288	0.76922947	9.86E-06	up	DENND1A	DENND1A
LOC418424	74.62381163	142.3569138	-0.931804605	9.86E-06	down	LOC418424	CD200
GPC2	933.4677721	527.2213982	0.824191288	9.87E-06	up	GPC2	
WNT5A	527.0076572	920.832344	-0.805114585	1.00E-05	down	WNT5A	WNT5A
USP32	558.6836932	303.2305187	0.881616751	1.00E-05	up	USP32	USP32
PSTPIP1	2.686278737	21.73705139	-3.016475337	1.04E-05	down	PSTPIP1	PSTPIP1
LOC107050933	109.4061948	223.8794239	-1.033027511	1.06E-05	down	LOC107050933	
RIF1	836.1809458	1506.803293	-0.849604018	1.07E-05	down	RIF1	RIF1
GTF3C4	86.34881185	239.5271712	-1.471941088	1.15E-05	down	GTF3C4	GTF3C4
ARHGEF3	330.7073519	85.52657579	1.951110433	1.15E-05	up	ARHGEF3	ARHGEF3
ALDH18A1	2949.326499	1317.738084	1.162321895	1.15E-05	up	ALDH18A1	
SLC35G1	469.4033042	177.2691615	1.404886417	1.16E-05	up	SLC35G1	SLC35G1
NF2L	24.62308919	57.69457027	-1.228423779	1.17E-05	down	NF2L	
PRPF38B	965.2465318	476.0120299	1.019899431	1.22E-05	up	PRPF38B	PRPF38B
LOC107056777	413.020387	184.4175315	1.163237185	1.22E-05	up	LOC107056777	
SNAPC1	395.4299218	190.7670598	1.051609963	1.25E-05	up	SNAPC1	SNAPC1
CAMK2D	711.7374808	413.8810408	0.782129049	1.25E-05	up	CAMK2D	CAMK2D
FILIP1	216.2319303	122.9157906	0.814909311	1.27E-05	up	FILIP1	FILIP1
ASTE1	8.539286057	37.45917435	-2.13313174	1.34E-05	down	ASTE1	ASTE1
PARD6B	368.0449887	150.0120554	1.294803683	1.35E-05	up	PARD6B	PARD6B
CCNL2	1330.570911	2039.441467	-0.616128703	1.35E-05	down	CCNL2	CCNL2
SLC25A33	362.6746111	97.17445684	1.90002671	1.37E-05	up	SLC25A33	SLC25A33
TECPR1	55.32103686	110.5958373	-0.999396983	1.39E-05	down	TECPR1	TECPR1
STYX	649.77546	297.2665621	1.128184067	1.45E-05	up	STYX	
TNFRSF1A	210.7480047	431.6230272	-1.034252863	1.45E-05	down	TNFRSF1A	TNFRSF1A
DENND3	428.3525204	145.2868033	1.559894912	1.47E-05	up	DENND3	DENND3
GJC2	313.6127917	535.1834316	-0.771049047	1.48E-05	down	GJC2	GJC1
UVSSA	256.5692656	161.0610273	0.67174092	1.50E-05	up	UVSSA	UVSSA
NOC2L	978.8256775	1507.910365	-0.623426819	1.54E-05	down	NOC2L	xp_005244796
PSAT1	423.5144144	149.6429291	1.500886962	1.56E-05	up	PSAT1	PSAT1
NUFIP1	491.0965363	264.927367	0.890409765	1.57E-05	up	NUFIP1	NUFIP1
PCF11	192.7204999	561.7866804	-1.543512379	1.65E-05	down	PCF11	PCF11
CCDC85C	573.463546	309.0123511	0.892037277	1.66E-05	up	CCDC85C	CCDC85C

GUCY1A2	80.59124035	35.64270792	1.177016089	1.66E-05	up	GUCY1A2	GUCY1A2
LRIG2	578.6175557	929.2518296	-0.683459527	1.69E-05	down	LRIG2	LRIG3
LOC422372	257.8598945	430.1008698	-0.738087644	1.74E-05	down	LOC422372	
LOC422301	142.1933547	239.1722688	-0.75019608	1.75E-05	down	LOC422301	xp_005277951
LOC107057150	9.937006082	53.63433562	-2.432273728	1.77E-05	down	LOC107057150	
MAP3K5	2425.725022	1180.938052	1.03848273	1.82E-05	up	MAP3K5	
COQ5	404.0862998	231.8697254	0.801348975	1.89E-05	up	COQ5	COQ5
IPPK	519.2479689	212.2740349	1.290495755	1.95E-05	up	IPPK	xp_005252191
TBCCD1	115.3165687	291.6065842	-1.338423481	1.99E-05	down	TBCCD1	TBCCD1
LOC107055658	10701.20924	82.21474084	7.024161028	2.03E-05	up	LOC107055658	
MTFR1L	723.3460549	475.0310943	0.606664058	2.03E-05	up	FAM54B	xp_005245999
LOC107056573	55.54885805	24.08632347	1.205543056	2.13E-05	up	LOC107056573	
FASLG	50.38821643	15.59308853	1.692179679	2.16E-05	up	FASLG	FASLG
HIST1H2B7	55.89211072	11.27856425	2.309061232	2.18E-05	up	HIST1H2B7	HIST2H2BE
LOC107056112	48.05702076	11.60102768	2.0504946	2.19E-05	up	LOC107056112	
SNAPC3	27.16430563	121.7654446	-2.1643207	2.25E-05	down	SNAPC3	xp_005251617
FAM160A1	271.3877341	146.6527939	0.88795096	2.34E-05	up	FAM160A1	FAM160A1
LOC107050861	45.35921177	130.952208	-1.52957291	2.35E-05	down	LOC107050861	
PHTF1	238.8203936	486.1294862	-1.025414605	2.36E-05	down	PHTF1	PHTF1
PELI2	194.7270899	113.0768462	0.784150051	2.45E-05	up	PELI2	PELI2
PRR5L	47.32627409	121.306964	-1.357949124	2.46E-05	down	PRR5L	xp_005253199
LOC107054527	60.50764416	26.00174847	1.218508775	2.46E-05	up	LOC107054527	
VEPH1	130.6176553	295.4877537	-1.177748424	2.53E-05	down	VEPH1	xp_005247822
MSANTD2	138.2825907	235.8624821	-0.770326414	2.57E-05	down	C11ORF61	MSANTD2
MCMBP	1956.733738	1050.401629	0.897506397	2.67E-05	up	MCMBP	MCMBP
IL17RA	2399.140175	1205.405121	0.992999353	2.82E-05	up	IL17RA	
TAF1D	703.6516073	454.3969623	0.630908106	3.02E-05	up	TAF1D	TAF1D
FAM65C	622.2309791	317.8915863	0.968915391	3.04E-05	up	FAM65C	
FBXW5	399.528585	1021.997085	-1.355020449	3.04E-05	down	FBXW5	FBXW5
SIX4	361.274563	223.7251452	0.691368265	3.07E-05	up	SIX4	SIX4
DCLRE1B	10.91834971	54.38209428	-2.316376899	3.12E-05	down	DCLRE1B	DCLRE1B
CTNNBL1	543.2871553	927.4016396	-0.771479339	3.19E-05	down	CTNNBL1	CTNNBL1
SLC33A1	845.7521812	508.6186267	0.733650695	3.40E-05	up	SLC33A1	SLC33A1
TRUB2	174.4291094	400.0760394	-1.197633405	3.43E-05	down	TRUB2	
LOC107055055	55.20865869	17.78640158	1.634119887	3.54E-05	up	LOC107055055	
LOC100857745	135.9188475	268.9598847	-0.984645487	3.62E-05	down	LOC100857745	
SMYD4	103.8958668	165.9426498	-0.675546466	3.75E-05	down	SMYD4	SMYD4
ZFPM2	167.6676089	95.34882365	0.814316962	3.75E-05	up	ZFPM2	ZFPM2
TAX1BP1	2275.404086	967.7103231	1.233475617	3.78E-05	up	TAX1BP1	TAX1BP1
HIST1H2B8	27.85199612	5.717984869	2.28420202	3.80E-05	up	HIST1H2B8	HIST2H2BE
PPIG	1743.164782	1002.55538	0.798027023	3.81E-05	up	PPIG	PPIG
FBXO28	428.3214313	207.3901474	1.046346508	3.82E-05	up	FBXO28	
LOC107050887	0.611513454	112.9277431	-7.528800004	4.08E-05	down	LOC107050887	
USP53	752.2982507	365.6744317	1.04074498	4.18E-05	up	USP53	USP53
CANX	4559.033806	2273.257459	1.00396702	4.26E-05	up	CANX	xp_005266054
KIAA1211L	133.0526271	82.635467	0.687163975	4.44E-05	up	KIAA1211L	KIAA1211L
RFWD2	150.9715439	272.059384	-0.849644944	4.67E-05	down	RFWD2	
PEX11G	70.18853153	33.29812754	1.075794268	4.78E-05	up	PEX11G	PEX11G
SH3BP2	101.903112	38.62221685	1.39969523	4.88E-05	up	SH3BP2	SH3BP2
PPAPDC1A	183.4344098	59.5792105	1.622383383	4.89E-05	up	PPAPDC1A	PPAPDC1A
GTPBP2	53.63579727	109.8462929	-1.03421808	4.91E-05	down	GTPBP2	GTPBP2
FGFR1OP2	686.7086066	219.0604561	1.648368967	4.94E-05	up	FGFR1OP2	FGFR1OP2
GRB10	596.7178802	228.5773615	1.384366482	4.95E-05	up	GRB10	xp_005271821
LOC107053964	9.061333716	31.06561357	-1.777523231	5.06E-05	down	LOC107053964	

LOC101747384	84.10956195	34.07038402	1.303751613	5.08E-05	up	LOC101747384	
RASSF2	26.15478954	99.45556821	-1.926976984	5.11E-05	down	RASSF2	RASSF2
LOC107054098	12.07730317	40.23601966	-1.736189253	5.16E-05	down	LOC107054098	
BRAF	813.7767299	426.332565	0.932653769	5.17E-05	up	BRAF	BRAF
ATG14	128.7597543	242.2085615	-0.911568134	5.19E-05	down	ATG14	
CDYL	620.8148315	268.4491501	1.209514187	5.24E-05	up	CDYL	CDYL
LOC100859265	814.7013746	289.0020594	1.495191569	5.29E-05	up	LOC100859265	
EML1	372.9135875	230.9910109	0.691004655	5.37E-05	up	EML1	
AAR2	182.9681222	315.0236604	-0.783867873	5.46E-05	down	C20H20ORF4	xp_005260443
USP47	3562.738368	1561.320167	1.190220133	5.70E-05	up	USP47	USP47
SUZ12	486.6408392	267.4987278	0.863325367	5.75E-05	up	SUZ12	SUZ12
GTF2H4	725.6135792	1247.973408	-0.782313833	5.79E-05	down	GTF2H4	xp_005245279
CLEC16A	420.3481341	144.6233132	1.539284537	6.42E-05	up	CLEC16A	CLEC16A
CEMIP	294.655613	155.6690398	0.920547706	6.52E-05	up	CEMIP	
LOC107056992	50.84822234	13.06342744	1.960663873	6.64E-05	up	LOC107056992	
TDH	90.91092862	34.46344803	1.399386686	6.66E-05	up	TDH	
KIAA1644	194.2934979	113.7508399	0.772360424	6.97E-05	up	KIAA1644	KIAA1644
CIT	69.75638641	136.4324599	-0.967789719	7.07E-05	down	CIT	CIT
LOC101747670	59.18830795	114.1586749	-0.947656374	7.27E-05	down	LOC101747670	
LRRC47	159.6929784	303.0031495	-0.924031909	7.61E-05	down	LRRC47	
CTDP1	486.8841059	257.3687744	0.919741378	7.90E-05	up	CTDP1	CTDP1
SETBP1	158.9476328	97.21892265	0.709242479	7.92E-05	up	SETBP1	SETBP1
STAM2	453.7963954	164.6764362	1.462411017	7.99E-05	up	STAM2	STAM2
XIAP	254.3780588	106.2440508	1.259592179	8.40E-05	up	XIAP	XIAP
CYP46A1	395.1107667	235.7401329	0.745059773	8.59E-05	up	CYP46A1	CYP46A1
GPR146	671.4176457	418.2340947	0.682899777	9.24E-05	up	GPR146	GPR146
LOC423740	37.79805249	95.25601631	-1.333498312	9.28E-05	down	LOC423740	PRSS12
CACUL1	539.6202698	306.9733078	0.813831327	9.43E-05	up	CACUL1	
LOC107054426	5.308484027	27.69006681	-2.382996708	9.64E-05	down	LOC107054426	
CDC34	406.0155275	227.4623521	0.835907122	9.97E-05	up	CDC34	CDC34
SLCO5A1	132.5305795	243.6390318	-0.878419998	1.00E-04	down	SLCO5A1	
LRP5	848.5666068	537.3804864	0.659083972	1.04E-04	up	LRP5	LRP5
LOC768814	85.34202777	0.839883236	6.666923807	1.04E-04	up	LOC768814	
ATP2B1	1964.508753	865.0877532	1.183250206	1.06E-04	up	ATP2B1	xp_005268977
EIF4EBP2	222.8118825	388.9791748	-0.803866745	1.09E-04	down	EIF4EBP2	
PBX4	87.85366149	159.8464397	-0.863512291	1.11E-04	down	PBX4	PBX3
LOC107055304	247.6302454	427.9466768	-0.789243509	1.11E-04	down	LOC107055304	
VWA2	64.52173605	138.0898709	-1.097750337	1.11E-04	down	VWA2	VWA2
FAM8A1	458.2330627	169.4462506	1.435253842	1.11E-04	up	FAM8A1	FAM8A1
GNPNAT1	1575.246505	613.2241168	1.361091267	1.13E-04	up	GNPNAT1	GNPNAT1
BICC1	3112.472787	1770.681657	0.813756364	1.14E-04	up	BICC1	BICC1
ARF1	4933.628095	1752.1332	1.493536512	1.18E-04	up	ARF1	ARF1
LOC107054899	13.89841818	59.83971922	-2.106182711	1.25E-04	down	LOC107054899	
RASL11B	148.1655953	293.270269	-0.985020336	1.26E-04	down	RASL11B	RASL11B
ARHGAP25	3.256194377	24.75576123	-2.926505584	1.26E-04	down	ARHGAP25	ARHGAP25
TRMT2A	208.862543	322.7620992	-0.627917392	1.28E-04	down	TRMT2A	TRMT2A
CRTC1	523.6710742	998.7556291	-0.931470812	1.30E-04	down	CRTC1	CRTC1
CAPRIN2	287.815975	487.5492101	-0.760401172	1.35E-04	down	CAPRIN2	
FOXK1	361.4459296	187.6176107	0.945984591	1.39E-04	up	FOXK1	
ADRB2	234.4126626	92.74644584	1.337686602	1.39E-04	up	ADRB2	
CABLES2	103.0575538	46.00907047	1.163460039	1.40E-04	up	CABLES2	
LOC107052380	72.3197668	210.9372557	-1.544351996	1.40E-04	down	LOC107052380	
KMT2E	812.6993694	1296.485948	-0.67381289	1.41E-04	down	KMT2E	KMT2E
ITGB1BP1	323.2035861	164.886354	0.970971199	1.42E-04	up	ITGB1BP1	ITGB1BP1

ARPP21	84.69320307	164.1861576	-0.955014402	1.43E-04	down	ARPP21	ARPP21
LOC100858919	11.08823102	48.66473738	-2.133847549	1.43E-04	down	LOC100858919	
LOC427259	224.757639	59.17531664	1.925302725	1.44E-04	up	LOC427259	RFK
C12ORF50	93.791596	37.76694776	1.312334466	1.46E-04	up	C12ORF50	C12orf50
AHCY	954.251147	557.5363978	0.775303024	1.48E-04	up	AHCY	xp_005260372
KATNA1	188.285493	100.5115795	0.905560131	1.50E-04	up	KATNA1	KATNA1
BIN1	1353.173417	737.182622	0.876252774	1.51E-04	up	BIN1	BIN1
CBLB	1282.015499	825.1929686	0.635610271	1.52E-04	up	CBLB	CBLB
GTF3C1	478.0933648	809.5318639	-0.759795484	1.53E-04	down	GTF3C1	GTF3C1
LOC107052931	26.4442042	62.89141089	-1.249911441	1.54E-04	down	LOC107052931	
CHKA	1542.915111	869.2477811	0.827819304	1.59E-04	up	CHKA	CHKA
LOC107056761	118.5748063	45.66520568	1.376630275	1.69E-04	up	LOC107056761	
LOC107051653	70.04272082	23.5105246	1.574928395	1.70E-04	up	LOC107051653	
HSF2	964.6523974	576.9947098	0.741451084	1.73E-04	up	HSF2	HSF2
CHPF2	511.1460554	1078.049339	-1.076615715	1.74E-04	down	CHPF2	xp_005250071
LOC107051754	16.44020795	55.19506598	-1.747310759	1.77E-04	down	LOC107051754	
BACH1	3485.484854	1666.455611	1.064576469	1.77E-04	up	BACH1	BACH1
RNF169	54.91093063	207.5154836	-1.918053718	1.88E-04	down	RNF169	
LOC107057245	26.93953775	4.451156384	2.597473003	1.90E-04	up	LOC107057245	
C11ORF24	659.0910289	432.1625818	0.60890357	1.94E-04	up	C11ORF24	
CUL3	2422.020185	1136.897415	1.091108806	1.97E-04	up	CUL3	CUL3
LOC107053639	43.34238637	81.81787145	-0.916637417	2.00E-04	down	LOC107053639	
TRIM41	102.468516	311.035418	-1.60189817	2.04E-04	down	TRIM41	TRIM41
HIST1H2BF	92.87388908	23.04845525	2.010602987	2.09E-04	up	HIST1H2BF	
DGKB	1385.166813	645.2688688	1.102087399	2.11E-04	up	DGKB	DGKB
ZSWIM3	5.40845846	29.44271186	-2.444621204	2.12E-04	down	ZSWIM3	ZSWIM3
LOC107052504	55.17319445	99.34239881	-0.848442071	2.13E-04	down	LOC107052504	
ACSL1	1646.7078	757.6253429	1.120028083	2.19E-04	up	ACSL1	ACSL1
DUSP7	357.2578546	643.6901114	-0.849400575	2.21E-04	down	DUSP7	
ABCG1	130.1814198	52.31506265	1.315225259	2.27E-04	up	ABCG1	ABCG1
FBXL7	159.8565896	296.2388802	-0.889982785	2.29E-04	down	FBXL7	FBXL7
ATXN2	560.909408	896.2891376	-0.676196431	2.32E-04	down	ATXN2	
SUCO	737.6556605	454.3622799	0.699104449	2.32E-04	up	SUCO	
GALNT2	2687.920874	1169.480881	1.200622393	2.34E-04	up	GALNT2	
RPUSD2	93.14099164	220.9890109	-1.246486483	2.39E-04	down	RPUSD2	RPUSD2
SLC25A21	78.50379813	26.94679628	1.542648695	2.45E-04	up	SLC25A21	SLC25A21
TMPO	933.3441099	521.3127921	0.840259817	2.46E-04	up	TMPO	TMPO
PIP4K2A	596.8945454	299.5170417	0.994837979	2.55E-04	up	PIP4K2A	PIP4K2A
CRYGN	7.800947465	30.72713632	-1.977792056	2.59E-04	down	CRYGN	CRYGN
AUTS2	395.7013052	845.8020687	-1.095908265	2.65E-04	down	AUTS2	AUTS2
CHM	976.1951953	296.6241889	1.718533401	2.76E-04	up	CHM	CHML
SLC5A1	38.98809512	13.15555202	1.567361882	2.78E-04	up	SLC5A1	SLC5A1
C4H20ORF194	2013.262424	1046.838944	0.943495735	2.82E-04	up	C4H20ORF194	C20orf194
KIF5B	5034.871931	3221.677046	0.644143202	2.84E-04	up	KIF5B	KIF5B
GPR183	28.55151702	7.579586793	1.913376296	2.85E-04	up	GPR183	GPR183
LIMK2	1627.675908	3561.287562	-1.129585465	2.85E-04	down	LIMK2	LIMK2
BNC2	89.3380672	50.95935485	0.80992803	2.87E-04	up	BNC2	
LOC100858320	134.039504	45.39510581	1.562049584	2.94E-04	up	LOC100858320	
REEP3	3374.938018	1249.917477	1.43302816	3.02E-04	up	REEP3	REEP3
SLC22A3	92.39868778	41.85036951	1.142632002	3.02E-04	up	SLC22A3	SLC22A3
STARD3NL	174.3631408	88.22454009	0.982843187	3.05E-04	up	STARD3NL	STARD3NL
LOC107056562	295.2539743	143.9750429	1.036137728	3.08E-04	up	LOC107056562	
GTPBP1	419.0390371	995.025027	-1.247648163	3.09E-04	down	GTPBP1	GTPBP1
GNAI1	775.5922435	377.4380733	1.039058416	3.13E-04	up	GNAI1	GNAI1

TSC22D1	640.4350991	1076.963996	-0.749845738	3.22E-04	down	TSC22D1	TSC22D1
LOC101748144	132.9239069	203.7959232	-0.61652459	3.25E-04	down	LOC101748144	
GPR21	103.3268362	165.6081005	-0.680558241	3.29E-04	down	GPR21	GPR21
FAM19A5	92.83682987	42.5705208	1.12484252	3.38E-04	up	FAM19A5	
TPGS2	230.945449	128.8750799	0.841578795	3.39E-04	up	TPGS2	
LOC101749321	8.154572173	28.8246156	-1.821620275	3.47E-04	down	LOC101749321	
PPP3CA	512.0743306	289.6988706	0.821799181	3.48E-04	up	PPP3CA	PPP3CB
TTF2	58.53227997	123.6498134	-1.078955682	3.54E-04	down	TTF2	TTF2
FITM2	188.0236659	107.0241085	0.812978443	3.62E-04	up	FITM2	FITM2
LOC418927	505.3697061	240.2571678	1.072759713	3.65E-04	up	LOC418927	POLR1D
ACSS2	101.241114	182.7512253	-0.852085792	3.68E-04	down	ACSS2	
SNX10	298.5826652	106.3316872	1.48955882	3.71E-04	up	SNX10	SNX10
BAIAP2L1	904.5473772	360.0329345	1.329067184	3.72E-04	up	BAIAP2L1	BAIAP2L1
LIN7B	909.9793483	380.053806	1.259630122	3.73E-04	up	LIN7B	LIN7C
LOC107051508	48.66721246	93.48963543	-0.941856289	3.81E-04	down	LOC107051508	
ETF1	3856.531652	1732.071702	1.154805298	3.83E-04	up	ETF1	ETF1
SLMO1	546.4114242	202.2945982	1.433529851	3.89E-04	up	SLMO1	PRELID3B
CAMKK1	170.1388916	107.3374052	0.664560043	3.93E-04	up	CAMKK1	CAMKK1
MAGI1	1135.877715	2254.352484	-0.988905581	3.94E-04	down	MAGI1	
ST3GAL5	290.8189405	97.83362485	1.571718931	4.07E-04	up	ST3GAL5	ST3GAL5
RDH10	456.2185807	237.210253	0.943558836	4.11E-04	up	RDH10	RDH10
FAM84B	295.912034	192.3471343	0.621456033	4.11E-04	up	FAM84B	FAM84B
LOC107054512	102.0751885	34.14022091	1.580087934	4.12E-04	up	LOC107054512	
KCNMA1	77.19187877	38.73042311	0.99498181	4.13E-04	up	KCNMA1	KCNMA1
IRAK2	2220.580184	1323.055337	0.747063263	4.14E-04	up	IRAK2	IRAK2
AFG3L2	1389.649911	821.1818391	0.758947849	4.14E-04	up	AFG3L2	AFG3L2
RUNX1	989.8671833	561.891617	0.816943086	4.15E-04	up	RUNX1	RUNX1
MIR103A1	28.31275045	5.942117705	2.252402819	4.21E-04	up	MIR103-1	
SPSB3	73.99260265	130.2854554	-0.816223085	4.27E-04	down	SPSB3	SPSB3
PLA2G4A	1157.095099	519.197244	1.156152811	4.44E-04	up	PLA2G4A	PLA2G4A
SEPT2	1855.158903	741.0042398	1.323989063	4.47E-04	up	LOC416777	SEPT2
CCDC92	341.2989546	209.1838642	0.706264424	4.50E-04	up	CCDC92	CCDC92
C1H21ORF59	132.2456765	73.04826277	0.856298688	4.52E-04	up	C1H21ORF59	C21orf59
FAM107A	48.41912241	219.557956	-2.180952978	4.54E-04	down	FAM107A	xp_005264890
RAB28	164.2908327	58.40015419	1.492207898	4.58E-04	up	RAB28	RAB28
P2RY11	31.05303073	138.4501566	-2.1565607	4.60E-04	down	P2RY11	
ITGA2	723.4063939	257.8543743	1.488249829	4.63E-04	up	ITGA2	ITGA2
DDX10	779.0873526	484.4726134	0.68536998	4.65E-04	up	DDX10	DDX10
UBE3C	810.9162332	395.3475372	1.036431456	4.79E-04	up	UBE3C	UBE3C
TMEM38B	168.589664	73.73232269	1.193146979	4.94E-04	up	TMEM38B	TMEM38B
MIPOL1	407.4475536	249.6802308	0.706532773	5.06E-04	up	MIPOL1	MIPOL1
LOC107050372	37.38310751	0	20	5.14E-04	up	LOC107050372	
TMX4	888.3751453	489.8760891	0.858752155	5.26E-04	up	TMX4	TMX4
DST	3920.606103	2600.912723	0.592058715	5.56E-04	up	DST	DST
UXS1	731.6768343	335.9961668	1.122761808	5.62E-04	up	UXS1	UXS1
MIR92A1	0.317723722	10.29893937	-5.018579151	5.63E-04	down	MIR92A1	
MAL2	414.4432108	266.6475225	0.636240506	5.86E-04	up	MAL2	MAL2
TMEM180	475.0766469	719.8106054	-0.599457068	6.12E-04	down	TMEM180	xp_005270211
ERI2	22.46309658	50.37286184	-1.165089877	6.12E-04	down	ERI2	ERI2
FGF2	307.711901	199.3948827	0.625951857	6.19E-04	up	FGF2	xp_005262893
ELMSAN1	769.3302535	451.0418121	0.770341863	6.21E-04	up	C5H14ORF43	ELMSAN1
CHST9	180.5653978	109.590883	0.720393668	6.49E-04	up	CHST9	CHST9
TMEM71	41.90876535	14.080658	1.573537266	6.52E-04	up	TMEM71	
EIF4E2	497.1458504	824.8466425	-0.73045675	6.57E-04	down	EIF4E2	xp_005247030

TMEM260	83.94435585	142.3237832	-0.761671534	6.65E-04	down	C5H14ORF101	TMEM260
HIST1H46L4	99.78370713	26.97826381	1.887006762	6.72E-04	up	HIST1H46L4	xp_005277469
SEPP1	74.09986887	119.5035376	-0.689510431	6.74E-04	down	SEPP1	SEPP1
MSH6	158.5686042	381.8735683	-1.267987913	6.78E-04	down	MSH6	
WISP1	654.7298672	1216.592292	-0.893874071	6.85E-04	down	WISP1	WISP1
ACADS	231.8260967	425.1364644	-0.874883026	6.90E-04	down	ACADS	ACADS
KL	120.9696271	218.5186455	-0.853111523	6.91E-04	down	KL	KL
UBE3A	1459.601063	775.1880908	0.912955795	7.04E-04	up	UBE3A	UBE3A
MGAT3	204.3869484	121.3276017	0.752395276	7.09E-04	up	MGAT3	MGAT3
TBC1D5	672.90482	337.6175166	0.995012698	7.10E-04	up	TBC1D5	
LOC101747432	2.176198073	25.28559306	-3.538433837	7.12E-04	down	LOC101747432	
GPR137C	70.64402577	166.6116083	-1.237849456	7.38E-04	down	GPR137C	
LPGAT1	1651.398885	754.4019034	1.130283415	7.38E-04	up	LPGAT1	LPGAT1
MAMLD1	183.6831002	561.7266822	-1.612649436	7.43E-04	down	MAMLD1	xp_005278219
LOC107056747	113.7843931	24.27195123	2.228940691	7.44E-04	up	LOC107056747	
CDC42EP3	2150.283524	1057.574427	1.023767701	7.80E-04	up	CDC42EP3	CDC42EP3
LOC107056127	943.8962465	394.4594465	1.258751299	7.96E-04	up	LOC107056127	
TXNRD3	1855.489939	768.4053982	1.271860619	8.08E-04	up	TXNRD3	TXNRD3
BCL10	792.7157245	407.7314393	0.95918439	8.09E-04	up	BCL10	BCL10
IFNGR1	300.6139376	94.66593874	1.666994564	8.28E-04	up	IFNGR1	IFNGR1
APPL1	677.5433755	349.6786071	0.954283773	8.33E-04	up	APPL1	APPL1
TNFSF15	143.2349431	76.64042563	0.902206012	8.42E-04	up	TNFSF15	TNFSF15
DNAJC27	41.85520054	99.36730378	-1.24736433	8.53E-04	down	DNAJC27	DNAJC27
ESRRG	124.9813872	67.22422699	0.894660092	8.60E-04	up	ESRRG	ESRRG
TGIF2	87.43785843	341.5424852	-1.965735078	8.65E-04	down	TGIF2	TGIF2
COMMD10	142.1438648	67.55206488	1.073280057	8.67E-04	up	COMMD10	COMMD10
RNF128	500.6975699	321.0204917	0.641274062	8.76E-04	up	RNF128	RNF128
YDJC	35.32514797	79.6059273	-1.172180251	8.88E-04	down	YDJC	YDJC
SESN3	71.15715975	29.01569875	1.294175254	9.05E-04	up	SESN3	SESN1
DDRGK1	1670.538189	913.882878	0.870231775	9.18E-04	up	DDRGK1	
TBC1D2	158.2580357	246.127689	-0.637128213	9.20E-04	down	TBC1D2	TBC1D2
STAM	643.4436094	257.9202154	1.318888864	9.38E-04	up	STAM	STAM2
ARHGAP10	548.294858	291.2896769	0.91249737	9.48E-04	up	ARHGAP10	ARHGAP10
LOC107056445	95.18056588	35.14655375	1.437283797	9.51E-04	up	LOC107056445	
ACAP2	518.7700106	268.5267204	0.950029428	9.52E-04	up	ACAP2	ACAP2
CCDC71L	25.93893534	119.8797693	-2.208397041	9.62E-04	down	CCDC71L	
TIGD5	16.88475681	100.2715816	-2.570119477	9.66E-04	down	TIGD5	
MNR2	3.141336235	23.83920712	-2.923885977	9.83E-04	down	MNR2	MNX1
SAP30L	709.6455	342.0372055	1.052945249	0.001001262	up	SAP30L	SAP30L
PNISR	642.6684501	1177.55617	-0.873649324	0.001001397	down	PNISR	PNISR
SOCS4	54.03204141	106.8256443	-0.983370923	0.001014958	down	SOCS4	SOCS4
TYW1	80.87306309	345.1672359	-2.093564367	0.001027772	down	TYW1	
LOC107055768	2.734146718	18.64864037	-2.769907883	0.00104629	down	LOC107055768	
NR1D2	130.9794568	81.37781835	0.686633044	0.00105722	up	NR1D2	NR1D2
LOC107054104	14.68899982	1.262136778	3.540795997	0.001059649	up	LOC107054104	
LMBR1	177.5046406	80.0656508	1.148601397	0.001059972	up	LMBR1	xp_005249611
LMF1	153.3968806	290.8224627	-0.92286956	0.001061451	down	LMF1	LMF2
JAM3	1160.463503	707.3101774	0.714286224	0.001063377	up	JAM3	JAM3
UPP1	104.2309542	47.48857396	1.13413145	0.001069085	up	UPP1	xp_005249894
C19H17ORF85	258.9232486	122.4851078	1.079918158	0.001081275	up	C19H17ORF85	NCBP3
PIGW	47.69288742	91.33158221	-0.937339698	0.001086554	down	PIGW	xp_005276951
CSF2RB	3.005460816	15.79037769	-2.393387563	0.001102311	down	CSF2RB	CSF2RB
BLK	6.46597913	22.33216412	-1.788182308	0.001112097	down	BLK	BLK
TPST1	447.5622297	271.8067444	0.719507036	0.001123077	up	TPST1	TPST1

ZNF821	314.8928466	722.6617715	-1.198459594	0.001146355	down	ZNF821	xp_005256091
NSMCE4A	381.6604259	216.9934881	0.814637854	0.001148133	up	NSMCE4A	NSMCE4A
TMEM86A	175.2985	90.54973874	0.953031268	0.001165617	up	TMEM86A	TMEM86A
ANAPC10	99.32352614	36.1409365	1.45850159	0.001167335	up	ANAPC10	ANAPC10
CCPG1	1478.761186	673.5852951	1.134456534	0.00116871	up	CCPG1	CCPG1
PIDD1	39.92346587	102.2967272	-1.357451113	0.001183326	down	PIDD1	
ORMDL3	453.3022624	769.901566	-0.764200643	0.001186134	down	ORMDL3	ORMDL3
CYB5D2	75.70615131	171.5142756	-1.179846229	0.001214116	down	CYB5D2	CYB5D2
TRIM32	154.0998916	257.5772458	-0.741139306	0.001239396	down	TRIM32	
NSD1	543.3025066	1551.762895	-1.514080527	0.001254249	down	NSD1	
NOP14	707.5073443	428.6251717	0.723028547	0.001266899	up	NOP14	NOP14
FANCM	294.2968533	982.6197962	-1.739361188	0.001271643	down	FANCM	
MCU	543.5165869	335.4478526	0.696235555	0.001286738	up	MCU	MCU
MRPS7	312.9328144	535.5117028	-0.775065155	0.001288714	down	MRPS7	MRPS7
CAMK1D	284.5326183	174.9387371	0.701744266	0.001290686	up	CAMK1D	CAMK1D
ACBD6	144.0710879	252.2812389	-0.808252078	0.001329515	down	ACBD6	ACBD6
ALDH6A1	174.2046532	101.904278	0.773568542	0.001329745	up	ALDH6A1	ALDH6A1
LRRC41	165.3593256	276.9354243	-0.743945198	0.001336122	down	LRRC41	LRRC41
SRBD1	330.6041876	155.1591367	1.091356345	0.001356376	up	SRBD1	SRBD1
TMEM204	61.08268329	106.243368	-0.798537443	0.001411302	down	TMEM204	TMEM204
ARHGAP12	950.8697339	346.0954753	1.45807763	0.001423722	up	ARHGAP12	
FARP2	226.1251318	358.7282002	-0.665769819	0.001459485	down	FARP2	FARP2
PLA2G4B	120.4529761	54.194532	1.152250838	0.001495183	up	PLA2G4B	
ZFP36L1	4187.370369	1746.124825	1.261887832	0.001496629	up	ZFP36L1	
FYTTD1	1944.393506	1083.409456	0.843741633	0.001512447	up	FYTTD1	FYTTD1
NLRP3	24.99291936	130.3900051	-2.383241951	0.001518528	down	NLRP3	
CD3E	140.0631295	211.3316174	-0.593431397	0.001531749	down	CD3E	CD3E
LOC107054268	25.70308549	5.670367807	2.180427333	0.001569917	up	LOC107054268	
GNL2	1048.086744	578.5092784	0.857346122	0.001630235	up	GNL2	
CEP85	60.2569106	184.3743436	-1.613439301	0.001670274	down	CEP85	CEP85
ACTR5	112.2270387	207.3658663	-0.885758132	0.0016833	down	ACTR5	ACTR5
WAC	1300.272709	812.4906192	0.678391173	0.001692118	up	WAC	
STX11	59.59417571	11.81281635	2.334818373	0.001693993	up	STX11	xp_005267245
CALCOCO2	934.8561263	612.5245328	0.609976724	0.001717004	up	CALCOCO2	xp_005256988
TTI1	99.95401363	270.8176902	-1.437985576	0.001718803	down	TTI1	TTI1
TMEM255A	477.7979531	285.8067356	0.741360757	0.001745309	up	TMEM255A	
LOC415787	42.33521365	15.983043	1.405316062	0.001754655	up	LOC415787	CES1
TRIB2	1337.059991	563.410503	1.246805834	0.001763507	up	TRIB2	TRIB2
STK17B	409.1868063	210.2033597	0.960973899	0.001814636	up	STK17B	STK17B
NADK	803.9131592	345.4117416	1.218722541	0.001822099	up	NADK	NADK
CEP250	78.30675444	346.8269926	-2.147007525	0.001823251	down	CEP250	CEP250
GPR75	27.38818848	62.77851293	-1.196717009	0.001844625	down	GPR75	GPR75
IL4R	927.3247767	562.4439645	0.721365332	0.001868637	up	IL4R	IL4R
TRAF2	346.5422338	851.0685516	-1.296244155	0.001873441	down	TRAF2	
LOC107051299	17.21444753	47.15079254	-1.45366214	0.001874991	down	LOC107051299	
RGS17	37.1373493	7.500637923	2.30778564	0.001878134	up	RGS17	RGS17
PRSS12	41.70735813	75.55011292	-0.857131981	0.001891749	down	PRSS12	PRSS12
DDAH1	688.2417364	445.4427913	0.627675226	0.001917051	up	DDAH1	DDAH1
C1QC	44.88751207	131.3097914	-1.548588457	0.001941596	down	C1QC	xp_005246040
SLMO2	4816.869742	2554.058276	0.915304467	0.001965537	up	SLMO2	PRELID3B
LOC107054414	14.20854998	1.594543732	3.15554376	0.0019893	up	LOC107054414	
ST3GAL1	851.4388204	417.4552971	1.028281154	0.002001498	up	ST3GAL1	ST3GAL1
ERC1	4282.330871	2836.319018	0.594376459	0.002040408	up	ERC1	ERC1
ST3GAL3	132.6252938	311.518125	-1.231960159	0.002047551	down	ST3GAL3	ST3GAL3

LACC1	83.4574723	21.81765028	1.935545492	0.002051036	up	LACC1	LACC1
NKIRAS1	42.35433583	12.89304982	1.715916096	0.002056895	up	NKIRAS1	NKIRAS1
KCTD15	85.11060109	164.1767611	-0.947839186	0.002093767	down	KCTD15	xp_005259301
MIER2	139.5787414	443.6607073	-1.668377559	0.002132965	down	MIER2	MIER2
UBN1	270.7076355	450.5218649	-0.734861545	0.002150784	down	UBN1	UBN1
LOC107056966	41.9729754	9.193512249	2.190772704	0.002179774	up	LOC107056966	
LOC107049080	1.451284835	91.56913355	-5.97945877	0.002185409	down	LOC107049080	
MTCH1	51.75630564	116.7883166	-1.174089409	0.002199342	down	MTCH1	MTCH1
GLI2	222.7599396	469.2156235	-1.074761245	0.002209847	down	GLI2	xp_005263697
RBM26	805.6188312	455.7243842	0.82193584	0.002242705	up	RBM26	RBM26
LOC107057295	33.18763099	156.3443401	-2.236009437	0.002319757	down	LOC107057295	
RNGTT	464.9782211	277.8546546	0.742832735	0.00232283	up	RNGTT	RNGTT
ZEB1	226.1074795	138.7406096	0.704618587	0.002330025	up	ZEB1	ZEB1
SDR16C5	653.5602416	224.5550718	1.541250915	0.002339477	up	SDR16C5	SDR16C5
TLR1B	44.72143249	100.8795307	-1.173595164	0.002363891	down	TLR1B	
ZNF277	260.8409577	89.28372873	1.546701237	0.002375305	up	ZNF277	ZNF277
WEE1	581.5726647	928.8978897	-0.675560554	0.002404513	down	WEE1	WEE1
EFTUD2	1615.565434	3051.622067	-0.917537117	0.002412421	down	EFTUD2	EFTUD2
LOC107050147	2.734146718	23.47533264	-3.101983033	0.002475261	down	LOC107050147	
SPAST	958.1832284	557.4344148	0.781499487	0.002528991	up	SPAST	SPAST
FAM107B	1656.712231	928.04931	0.836049662	0.002598061	up	FAM107B	FAM107B
RP11-574K11.31	205.6956779	338.6744189	-0.71938754	0.002604505	down	RP11-574K11.31	
PLD6	40.04459417	81.73050644	-1.029267175	0.00262415	down	PLD6	FLCN
KDM4C	217.8486944	115.0414025	0.921173297	0.002628743	up	KDM4C	KDM4C
LOC107054816	172.3833631	52.32064069	1.720168432	0.00264828	up	LOC107054816	
LOC416357	48.01250623	87.7491593	-0.869975057	0.00270124	down	LOC416357	
TNFRSF18	44.25395972	14.29409669	1.630387096	0.002701271	up	TNFRSF18	TNFRSF18
LOC107053428	124.2107742	59.78499674	1.054934935	0.002706503	up	LOC107053428	
FAF1	595.0290525	359.9756756	0.725060686	0.002711898	up	FAF1	
MTMR6	888.205264	418.7251672	1.084889488	0.002724625	up	MTMR6	MTMR6
GNA13	1128.981517	488.0413637	1.209946534	0.002739314	up	GNA13	GNA13
LOC428754	25.80451828	8.172353674	1.658800153	0.002776846	up	LOC428754	HSD17B11
BCL2L11	88.5341968	335.4111004	-1.921623719	0.00283424	down	BCL2L11	
SCN8A	68.06195993	132.4197614	-0.960197835	0.002836352	down	SCN8A	SCN8A
FAM102B	305.664833	145.2962545	1.072953065	0.002843584	up	FAM102B	xp_005270829
IGFALS	1.846507356	15.73199863	-3.090831051	0.002899359	down	IGFALS	IGFALS
YLPM1	554.8838759	863.6812385	-0.63831307	0.002922844	down	YLPM1	
LOC107050010	8.13209654	26.85018357	-1.723232705	0.00293417	down	LOC107050010	
ARHGEF4	158.3435747	428.4040216	-1.435913697	0.00293571	down	ARHGEF4	ARHGEF4
C3ORF33	20.37009414	61.31750483	-1.589846344	0.00300256	down	C3ORF33	
FAM214B	0.941204171	19.25577104	-4.354639369	0.003004562	down	FAM214B	FAM214B
LIFR	178.1402246	108.4727724	0.715680359	0.00301504	up	LIFR	xp_005248360
SRRL	90.09654959	49.18297566	0.873312833	0.003030835	up	SRRL	
LOC107054364	0.317723722	13.9309859	-5.454380743	0.003041924	down	LOC107054364	
MIR1306	21.47402443	58.22281994	-1.438992125	0.003053052	down	MIR1306	
CLMN	548.9272252	277.8572213	0.982271159	0.003086537	up	CLMN	CLMN
INTS12	375.4979813	183.4817445	1.033168623	0.003088919	up	INTS12	INTS12
LOC770141	74.53142912	206.1305773	-1.467637701	0.003103076	down	LOC770141	
LOC420030	2.493921795	39.97586331	-4.002641059	0.003151006	down	LOC420030	LOC100507656
CELF1	1002.70364	528.2348537	0.924643864	0.003164757	up	CELF1	CELF1
C17ORF80	66.64525433	140.5737006	-1.076752659	0.003262509	down	C17ORF80	ATP5J2
FRMPD1	3.105435249	19.03784422	-2.61600273	0.003271412	down	FRMPD1	FRMPD1
ITGB8	120.9401329	65.20583448	0.891220105	0.003307585	up	ITGB8	ITGB8
LOC107052611	57.2254841	31.12241047	0.878703959	0.003367308	up	LOC107052611	

FER1L4	804.528357	1802.492799	-1.163778318	0.003395961	down	FER1L4	
KLHL3	118.7656933	197.3040997	-0.732302775	0.003413335	down	KLHL3	
CRYBA4	38.53960793	291.6040985	-2.919597197	0.003418883	down	CRYBA4	CRYBA4
LOC107051184	16.79383266	1.711233998	3.294822562	0.003509692	up	LOC107051184	
LOC422264	729.1288282	366.8773579	0.990875873	0.003524835	up	LOC422264	PCDH11X
AEBP2	169.9167672	88.71863708	0.937519116	0.003530214	up	AEBP2	AEBP2
HBG2	0.522047659	22.12411296	-5.405294283	0.003551757	down	HBG2	HBE1
EME1	53.60908316	158.8478402	-1.567096108	0.003555897	down	EME1	EME1
LOC107057413	1.145528108	31.42618801	-4.77788252	0.00358393	down	LOC107057413	
RCAN3	1186.292596	544.6063613	1.123174151	0.003598346	up	RCAN3	RCAN3
LOC100857473	11.06005856	0.871350762	3.665961622	0.00368939	up	LOC100857473	
CBS	646.1414222	424.8037093	0.605053601	0.00373197	up	CBS	CBS
RBM17	1546.976407	613.2639891	1.334871051	0.003739045	up	RBM17	RBM17
KLHL14	49.09382429	109.7097286	-1.160078005	0.003752171	down	KLHL14	KLHL14
PDPR	600.0645928	1625.052119	-1.437296279	0.003775125	down	PDPR	
TBL1XR1	748.3898032	428.7148998	0.803771344	0.003802746	up	TBL1XR1	TBL1XR1
STRN3	1098.669145	584.5177925	0.91043815	0.003812753	up	STRN3	
LOC107054983	0.635447444	45.36075846	-6.157528144	0.003835593	down	LOC107054983	
C2ORF88	291.9100611	645.2365286	-1.144304179	0.003869584	down	C2ORF88	
CTBP1	2537.442321	1522.61923	0.736819824	0.00389663	up	CTBP1	CTBP1
LOC107056588	67.08424296	16.30392427	2.040754684	0.003906079	up	LOC107056588	
MARCO	44.20900845	198.9148739	-2.169738876	0.003910009	down	MARCO	MARCO
P2RY6	5.738149177	40.96618233	-2.835776073	0.003945715	down	P2RY6	P2RY6
LOC107055675	1660.600065	61.58621304	4.75295343	0.00397407	up	LOC107055675	
SHOC2	516.9883251	265.2499286	0.962779338	0.003997763	up	SHOC2	xp_005270243
ZFYVE26	617.6412517	334.3006204	0.88562308	0.004055782	up	ZFYVE26	ZFYVE26
OSGIN2	128.1508959	57.818913	1.148230173	0.004078371	up	OSGIN2	
SUGP1	192.2587239	403.4545599	-1.069357132	0.004106155	down	SUGP1	SUGP1
MPEG1	11.91311869	114.5151005	-3.264914806	0.004182167	down	MPEG1	MPEG1
LOC101751485	2.404456001	14.00923894	-2.542596151	0.004201413	down	LOC101751485	
LOC769676	1898.64265	1053.055978	0.850386268	0.004211875	up	LOC769676	xp_005267079
MAP3K14	591.1113988	2027.507023	-1.778204965	0.004246584	down	MAP3K14	MAP3K14
SLC44A1	1033.41286	644.4621971	0.681249102	0.004297564	up	SLC44A1	SLC44A1
LOC107056461	175.0321343	51.34798552	1.769240229	0.004329258	up	LOC107056461	
MIR1725	2.493921795	17.62906396	-2.821467744	0.004356805	down	MIR1725	
PDZD8	446.5092476	296.2543381	0.591853776	0.00442257	up	PDZD8	PDZD8
PEX6	264.295073	669.9978809	-1.342007002	0.00444829	down	PEX6	
LOC107051523	4.298831338	128.8108603	-4.905165916	0.004527204	down	LOC107051523	
ABL2	651.3287548	1656.035531	-1.346275802	0.004538353	down	ABL2	ABL2
PXK	158.7736496	98.0120073	0.695941092	0.0045822	up	PXK	
BAIAP2L2	8.324453482	37.53218864	-2.172700957	0.004707236	down	BAIAP2L2	BAIAP2L2
NUBP1	657.947571	437.2707508	0.589445777	0.0047105	up	NUBP1	NUBP1
TAB3	1082.340355	601.4068991	0.84774092	0.004714345	up	TAB3	TAB3
IGDCC4	58.41026664	169.8648868	-1.540093784	0.004725411	down	IGDCC4	IGDCC4
LOC107056301	39.61638738	16.39611671	1.272743163	0.004727132	up	LOC107056301	
YES1	852.5637025	434.6659807	0.971900448	0.004730634	up	YES1	YES1
PNO1	221.1236012	363.9581323	-0.718919484	0.004804012	down	PNO1	PNO1
MCM9	37.56687785	71.91496992	-0.936830896	0.004804706	down	MCM9	
DPF3	14.12389599	1.864015636	2.921652235	0.004816047	up	DPF3	DPF3
CITED2	13.84631173	44.36111082	-1.679793758	0.004881629	down	CITED2	CITED2
DLG1	1415.914353	773.3947984	0.872457034	0.004890155	up	DLG1	xp_005269350
ASNS	3913.339855	1350.380886	1.535034018	0.004926328	up	ASNS	ASNS
NFIL3	1549.087925	587.7228171	1.398211219	0.004929686	up	NFIL3	NFIL3
LOC101748260	98.41371128	149.1022632	-0.599370922	0.00498083	down	LOC101748260	

SFMBT2	118.801021	67.81147062	0.808945997	0.004983297	up	SFMBT2	
DCP1B	49.10972962	100.6952685	-1.03591511	0.005055047	down	DCP1B	DCP1B
UNC119B	981.4367296	1576.107302	-0.683398588	0.0050608	down	UNC119B	MLEC
HPS4	180.1258243	347.9074035	-0.949698348	0.005063498	down	HPS4	HPS4
VNN1	2284.733714	1166.393293	0.969971699	0.005104366	up	VNN1	VNN1
ARHGAP11A	164.2054687	101.1043463	0.699657158	0.005120116	up	ARHGAP11A	xp_005254864
PSAP	8024.244998	5182.723206	0.63065531	0.005134295	up	PSAP	PSAP
SMYD3	52.98092751	21.30106202	1.314547739	0.005143193	up	SMYD3	SMYD3
LOC100858951	16.34504533	51.14836358	-1.645834705	0.005268702	down	LOC100858951	PHYHD1
LOC107055795	17.71985299	56.63517805	-1.676331804	0.005304814	down	LOC107055795	
ACPP	580.1199177	204.6609912	1.503115005	0.005320455	up	ACPP	ACPP
OIP5	65.83467703	36.46734102	0.852242682	0.00532443	up	OIP5	OIP5
LOC107050883	7.256424173	24.60297959	-1.76150235	0.005400117	down	LOC107050883	
LOC107056873	62.34876637	25.65319198	1.281222666	0.005414849	up	LOC107056873	
RPS6KA6	405.3472593	181.1568084	1.161919362	0.005541906	up	RPS6KA6	RPS6KA6
DOK3	8.321536767	67.89842431	-3.028456209	0.005632651	down	DOK3	xp_005266040
ATP10D	30.71005127	67.69636252	-1.140367396	0.005640083	down	ATP10D	ATP10D
NUCB2	5731.121384	3217.26233	0.832983877	0.005660821	up	NUCB2	NUCB2
GORASP1	1402.114872	725.8400817	0.949880919	0.005672522	up	GORASP1	GORASP1
CDC23	119.7293847	268.8660667	-1.167110416	0.005684668	down	CDC23	CDC23
ZNF407	474.0646739	306.217133	0.630528887	0.005858578	up	ZNF407	
PDK4	181.4591707	78.0104163	1.217906294	0.005918717	up	PDK4	PDK4
TPBG	161.4120873	274.7882009	-0.767571439	0.005929056	down	TPBG	TPBG
CYB5R4	149.396799	67.24187776	1.151717319	0.005956549	up	CYB5R4	CYB5R4
AKT3	475.3860418	251.4532968	0.918809075	0.005995034	up	AKT3	
RCBTB2	119.2449965	237.8691323	-0.996239337	0.006013268	down	RCBTB2	RCBTB2
EXOC3	1151.679536	567.8719797	1.020101699	0.006023308	up	EXOC3	EXOC3
SHQ1	369.0371411	166.6259329	1.147153068	0.006052537	up	SHQ1	SHQ1
LOC107055056	30.88328603	65.64506911	-1.087860382	0.006128888	down	LOC107055056	
ZC3H7B	648.0494018	1511.862821	-1.222151541	0.006187123	down	ZC3H7B	ZC3H7B
PTPN21	1189.356448	553.4943031	1.103540781	0.006189628	up	PTPN21	PTPN21
LOC769660	117.721598	243.6050107	-1.049164777	0.006252422	down	LOC769660	
VRK2	157.8155301	260.1087057	-0.720875504	0.006269011	down	VRK2	VRK2
BRCC3	169.0272328	323.5670619	-0.936809049	0.006272382	down	BRCC3	BRCC3
JAKMIP2	46.53874577	111.762753	-1.263935228	0.006340632	down	JAKMIP2	JAKMIP2
UBXN10	34.46422271	69.03537375	-1.002236311	0.00634531	down	UBXN10	UBXN10
TRMT61A	95.03228673	160.7091576	-0.75796249	0.006347685	down	TRMT61A	TRMT61A
SBDS	1922.711254	835.1567374	1.203023235	0.006491016	up	SBDS	SBDS
LOC419662	58.91275538	242.923008	-2.043847203	0.006496995	down	LOC419662	
SNX16	189.1674355	105.3036947	0.8451077	0.006501213	up	SNX16	SNX16
DENND6A	256.7269067	127.6188098	1.008393523	0.00657814	up	DENND6A	DENND6A
CRYM	155.5431477	317.825275	-1.030919019	0.006595162	down	CRYM	xp_005255183
MED12L	81.79063341	155.6860208	-0.928631868	0.006708176	down	MED12L	
MCF2	41.18239376	79.25668805	-0.944504992	0.006820644	down	MCF2	
TAF4B	114.1869459	64.98100581	0.813307748	0.006986199	up	TAF4B	xp_005258395
PRPF4	399.6902741	636.6859914	-0.671699555	0.007047822	down	PRPF4	PRPF4
DUSP16	379.9624863	711.2835701	-0.904567851	0.007053562	down	DUSP16	DUSP16
LAMP1	5104.681898	3016.014925	0.759177493	0.007091112	up	LAMP1	LAMP1
RP11-2C24.9	411.1262848	656.4212676	-0.67504037	0.007129179	down	RP11-2C24.9	
ADCK1	51.17588136	118.6461262	-1.213129048	0.007164726	down	ADCK1	ADCK1
TAF6L	35.84808065	160.2304048	-2.160180152	0.007236245	down	TAF6L	
SCAMP1	427.7634826	215.1244546	0.991641793	0.007305637	up	SCAMP1	SCAMP1
SAT1	5822.972201	3660.487378	0.669719981	0.007314091	up	SAT1	SAT1
EPS15	1504.72845	908.1025224	0.728576067	0.007319457	up	EPS15	xp_005270674

LACTB2	692.00147	262.3300113	1.399392237	0.007387249	up	LACTB2	LACTB2
LOC107053494	284.9419992	122.5740002	1.21701529	0.007416861	up	LOC107053494	
EHHADH	53.30421146	103.9650358	-0.963776994	0.007428803	down	EHHADH	EHHADH
LOC107056604	3.017427812	64.61666522	-4.420515135	0.007520309	down	LOC107056604	
MARS2	70.83987276	194.8900217	-1.460026701	0.007582503	down	MARS2	MARS2
STX12	876.4663901	420.2055268	1.060603632	0.0075851	up	STX12	STX12
UBXN7	214.1413962	415.7908444	-0.957294277	0.007634199	down	UBXN7	UBXN7
LOC107050288	29.7955612	77.41971967	-1.377603663	0.007711118	down	LOC107050288	
PPIL2	317.7203974	634.7129641	-0.998346597	0.007723662	down	PPIL2	PPIL2
IQSEC3	32.07041195	64.10207045	-0.99912807	0.007781842	down	IQSEC3	IQSEC3
LOC107051875	95.74318973	270.5740399	-1.498781651	0.007865517	down	LOC107051875	
RORA	84.56329769	36.3343655	1.218696925	0.00793928	up	RORA	RORA
SPSB4	43.23044494	20.1601715	1.100539773	0.00805521	up	SPSB4	SPSB4
DNAJA4	1232.309665	654.2927588	0.913356625	0.008060528	up	DNAJA4	DNAJA4
GON4L	487.6571757	1207.980319	-1.308657759	0.00806325	down	GON4L	
ATP6AP2	1069.086993	506.7397461	1.077062355	0.008069587	up	ATP6AP2	xp_005272630
GSTCD	201.3400379	106.5612754	0.917950836	0.008074119	up	GSTCD	
UGP2	1358.473105	641.6392188	1.082151772	0.008409022	up	UGP2	UGP2
YIPF1	343.7121607	203.3810507	0.757015628	0.008420937	up	YIPF1	YIPF1
LOC107054608	57.48072933	97.89222056	-0.768115847	0.008425881	down	LOC107054608	
LOC107050448	1.846507356	50.35478667	-4.769258011	0.00845491	down	LOC107050448	
LRCH2	402.9580853	197.566674	1.028290171	0.008580244	up	LRCH2	LRCH2
LOC100858649	245.8235889	90.47767864	1.441989541	0.00860661	up	LOC100858649	
ZMYM3	78.07076799	318.6664951	-2.029192974	0.008616573	down	ZMYM3	
TMEM169	222.243152	379.1812343	-0.770748601	0.008647337	down	TMEM169	TMEM169
BBS4	41.30498042	73.80140896	-0.837332612	0.008667571	down	BBS4	BBS4
ELF2	277.7390186	519.4137898	-0.903154446	0.008681497	down	ELF2	ELF2
CSNK1A1	4199.58784	2410.322478	0.801021566	0.008804452	up	CSNK1A1	CSNK1A1
RPIA	336.7010849	199.1684588	0.757479176	0.008826366	up	RPIA	RPIA
KCNK1	610.0192435	327.5803664	0.897005863	0.008846075	up	KCNK1	KCNK1
DEPDC5	349.9491758	620.7926896	-0.826966158	0.009068732	down	DEPDC5	DEPDC5
ID4	391.5678127	692.6028039	-0.822766049	0.009124948	down	ID4	ID4
IRX1	12.654374	36.40902982	-1.524660158	0.009199254	down	IRX1	IRX1
LOC107049214	22.05299035	52.08030353	-1.239763561	0.009227455	down	LOC107049214	
DGKH	178.4237789	100.1300411	0.833433021	0.009314392	up	DGKH	DGKH
DDEFL1	7.608590524	39.00086227	-2.357804894	0.00941436	down	DDEFL1	ASAP1
CCBL1	86.6503301	202.825627	-1.226962798	0.009435498	down	CCBL1	xp_005252352
ADARB1	693.9157467	397.8400546	0.802571971	0.009475065	up	ADARB1	ADARB1
LOC107057546	0.317723722	24.11641241	-6.246098687	0.009497523	down	LOC107057546	
GP5	25.42258451	56.58907529	-1.154412858	0.009558939	down	GP5	
LOC107054579	1.475218825	11.43976204	-2.955056166	0.009635408	down	LOC107054579	
FOXO1	322.8968462	154.8524835	1.060178829	0.009759612	up	FOXO1	FOXO1
LOC107054531	23.54163453	152.5702191	-2.696186986	0.009865785	down	LOC107054531	
CBX8	41.30162697	78.54502077	-0.927321207	0.009910307	down	CBX8	
TEAD1	2802.123297	1181.550479	1.245839171	0.010001371	up	TEAD1	xp_005253150
SPAG9	1115.05362	740.4111515	0.590714558	0.010058159	up	SPAG9	SPAG9
ZNF502	9.365632085	32.27020284	-1.784754376	0.010238188	down	ZNF502	ZSCAN2
GPCPD1	634.1926757	300.3647679	1.078205618	0.010328457	up	GPCPD1	xp_005260816
LYSMD2	327.4105102	142.9695964	1.195392255	0.010379627	up	LYSMD2	LYSMD2
NRBF2	420.9174764	215.5394497	0.965585464	0.010398256	up	NRBF2	NRBF2
LOC107052787	32.04180275	4.716072299	2.764297579	0.010474316	up	LOC107052787	
RIC1	754.7053387	408.2726522	0.886380547	0.010537155	up	RIC1	
TP53BP1	456.3644934	756.0240264	-0.728245534	0.010570615	down	TP53BP1	TP53BP1
ATRNL1	481.1607038	307.8735897	0.644180709	0.01065343	up	ATRNL1	ATRNL1

PIGL	77.22880138	35.63359594	1.115900901	0.010706656	up	PIGL	
LOC100859013	120.7415058	237.529561	-0.976185372	0.010725667	down	LOC100859013	MMAB
LOC107049790	3.232260387	17.40955497	-2.429263995	0.010823063	down	LOC107049790	
CASZ1	222.2885131	133.8644425	0.731660599	0.010845727	up	CASZ1	CASZ1
NAMPT	1201.023565	423.6589366	1.503289251	0.010891057	up	NAMPT	xp_005250158
EPS15L1	826.6060971	507.5277539	0.703713288	0.010938444	up	EPS15L1	
LOC107053673	14.17600244	43.12954216	-1.605225643	0.010954995	down	LOC107053673	
CPT1A	756.6448096	310.7856797	1.283696188	0.010971895	up	CPT1A	CPT1A
LOC427362	7.09850986	28.48151451	-2.004437757	0.011006909	down	LOC427362	MROH7
KCNJ16	1149.141563	2142.334669	-0.898627336	0.011497542	down	KCNJ16	KCNJ16
TRIP13	26.22032134	72.82544066	-1.473757159	0.011524827	down	TRIP13	
LOC107056074	0.317723722	9.356169553	-4.880073292	0.01153923	down	LOC107056074	
RBM5	1023.215376	2613.686813	-1.352976431	0.011569169	down	RBM5	RBM5
CIDEA	362.5987341	152.0474029	1.253852715	0.011590831	up	CIDEA	CIDEA
UBE2D1	285.952837	108.7107637	1.395282427	0.011668958	up	UBE2D1	UBE2D1
MIS12	25.77285577	66.74310213	-1.372766338	0.011698687	down	MIS12	MIS12
SUPT6H	1163.295869	2201.725308	-0.920416412	0.011744559	down	SUPT6H	SUPT6H
LOC107055997	22.49184238	3.388722476	2.730588447	0.01187022	up	LOC107055997	
TARS	1761.077327	672.7117801	1.388397831	0.012095515	up	TARS	TARS
FADS2	1196.890755	2013.450265	-0.750378358	0.012104077	down	FADS2	FADS2
FAM49B	736.8344226	361.062102	1.029093462	0.012112558	up	FAM49B	xp_005251021
CXCR4	28.19511219	6.813468466	2.048983775	0.012116725	up	CXCR4	CXCR4
LOC427201	587.475462	891.6486635	-0.601946763	0.012153636	down	LOC427201	
ZCCHC10	450.1197613	174.8341065	1.364322251	0.012238472	up	ZCCHC10	ZCCHC10
LOC107051288	0.317723722	16.79691411	-5.724279589	0.012263181	down	LOC107051288	
BRIP1	134.1858881	61.96457396	1.114717409	0.012317091	up	BRIP1	BRIP1
LOC107055376	39.723517	218.8334523	-2.461768029	0.012355216	down	LOC107055376	
SHB	805.8576478	445.9763387	0.853557844	0.012388484	up	SHB	SHB
RLIM	1110.22857	714.1645908	0.636528214	0.012425314	up	RLIM	RLIM
SAMD8	143.1743982	55.30823955	1.372207212	0.012661712	up	SAMD8	SAMD8
LOC107055600	2.610238295	29.0995429	-3.478743066	0.012750055	down	LOC107055600	
BBS9	159.4085237	76.29076616	1.063148417	0.012805226	up	BBS9	
KCTD7	123.919628	67.71858235	0.871781042	0.012873323	up	KCTD7	KCTD7
DPYSL4	32.57903425	63.76048366	-0.968718735	0.013109664	down	DPYSL4	DPYSL4
LOC100857724	2.505888791	23.4661528	-3.227187041	0.013114198	down	LOC100857724	xp_005277413
RPP25L	242.6193528	402.1841975	-0.729161764	0.01345309	down	RPP25L	
PRKCB	5.715673544	24.92399657	-2.124540001	0.013585917	down	PRKCB	
LOC107051503	0.635447444	10.04782714	-3.98296693	0.013624455	down	LOC107051503	
MCM3	285.6120682	814.5520619	-1.511949971	0.013931059	down	MCM3	MCM3
SLC25A36	577.849594	244.4274968	1.241287439	0.013993262	up	SLC25A36	SLC25A36
MBOAT2	226.2633621	95.49752848	1.244467693	0.014012916	up	MBOAT2	MBOAT2
TREM-B2	9.35366509	47.97542565	-2.358691928	0.014092351	down	TREM-B2	
WRN	323.6236046	488.0379415	-0.592676472	0.014139972	down	WRN	xp_005273689
SRRM4	4.784978011	18.61331268	-1.95975064	0.014300319	down	SRRM4	SRRM4
ITGA6	4165.120056	2108.963069	0.981824249	0.014423716	up	ITGA6	ITGA6
ISY1	514.2067587	272.8149308	0.91442597	0.014448664	up	ISY1	ISY1-RAB43
RYR1	20.55529589	74.89095798	-1.865281405	0.014467975	down	RYR1	
IMPA2	201.5116777	99.6147636	1.016431966	0.014474466	up	IMPA2	IMPA2
LOC107052159	90.8223363	353.0834291	-1.95889006	0.014551319	down	LOC107052159	
GEM	69.674349	24.21460729	1.524750012	0.014630157	up	GEM	GEM
TJAP1	174.8778852	496.1034389	-1.504293098	0.014654069	down	TJAP1	
LOC107049528	24.5771163	82.4259694	-1.745783299	0.014709099	down	LOC107049528	
PEX5	445.0637847	779.3794193	-0.808313724	0.014713068	down	PEX5	PEX5
RIMBP3C	8.00672976	42.22604111	-2.398847975	0.014723513	down	RIMBP3C	

SLC7A11	159.6181078	72.39242512	1.140713674	0.014943226	up	SLC7A11	SLC7A11
CMTM3	1425.870708	800.7297812	0.8324558	0.014988267	up	CMTM3	CMTM3
CDC73	1599.111096	831.7042458	0.94312767	0.015046231	up	CDC73	CDC73
SCML2	168.7519803	86.49239699	0.964259207	0.015206872	up	SCML2	SCML2
LOC107057347	138.9882067	317.4216395	-1.19143801	0.01525046	down	LOC107057347	
LOC107053186	73.78885206	320.9798115	-2.121007783	0.015274298	down	LOC107053186	
LOC417551	123.4168391	71.53659592	0.786785877	0.015306969	up	LOC417551	
LOC107054900	56.30587054	189.0127909	-1.747126615	0.015333143	down	LOC107054900	
LOC421935	54.40390328	94.09613814	-0.79042535	0.015347985	down	LOC421935	
CDH6	53.17941801	23.33317728	1.188485216	0.015625615	up	CDH6	CDH6
DSCC1	38.11723457	15.54933163	1.293590885	0.015719416	up	DSCC1	DSCC1
MLXIPL	4.365821499	19.63516709	-2.169114824	0.015725669	down	MLXIPL	MLXIPL
C5H14ORF105	8.867518417	57.0975867	-2.686827443	0.015755065	down	C5H14ORF105	xp_005267862
DHX57	253.2075635	382.2327641	-0.594130948	0.015786455	down	DHX57	DHX57
ELK3	2339.40222	3816.349695	-0.706053443	0.015987618	down	ELK3	ELK3
RBPJ	490.1573753	207.4730964	1.240320761	0.016049301	up	RBPJ	RBPJ
LOC770022	30.00148009	6.395838772	2.2298282	0.016058609	up	LOC770022	HIST2H3A
FCHSD2	104.640339	171.8650292	-0.715836898	0.016229976	down	FCHSD2	
GPR37L1	16.36416751	77.05888687	-2.235421136	0.016232311	down	GPR37L1	GPR37L1
CCSAP	49.27245575	23.40301418	1.074087025	0.016386327	up	CCSAP	
HARS2	71.53882032	178.8205982	-1.321714696	0.016752165	down	HARS2	HARS
LOC107056309	111.995564	36.41882456	1.620685325	0.017060121	up	LOC107056309	
CAPNS2	75.90274672	43.89800634	0.789996674	0.017066806	up	CAPNS2	CAPNS2
LOC107051392	7.189434012	26.78718066	-1.897592642	0.01715997	down	LOC107051392	
AVD	915.4139206	1610.842476	-0.815319284	0.017196664	down	AVD	
VPS54	802.8559502	460.6629393	0.801429623	0.017257672	up	VPS54	VPS54
LOC107052740	153.8716182	41.21328561	1.900545762	0.017337236	up	LOC107052740	
CA2	213.1301217	135.6350646	0.652004309	0.017459728	up	CA2	CA2
CDR2L	77.0711872	195.8301873	-1.345339655	0.017470845	down	CDR2L	CDR2L
LOC107053244	8.209595339	25.44900098	-1.632226007	0.017564728	down	LOC107053244	
UGDH	1641.555019	735.011074	1.159225213	0.017578606	up	UGDH	xp_005262725
MGA	662.7208475	1192.032106	-0.846949885	0.017663486	down	MGA	
NMT2	644.6848465	276.4546167	1.221551408	0.017809827	up	NMT2	NMT2
OAT	2637.386349	1146.687931	1.201636108	0.017892909	up	OAT	xp_005269925
SLC2A3	688.1365308	327.1870988	1.07257897	0.017988977	up	SLC2A3	SLC2A3
LRP3	108.6717945	186.3137784	-0.777756828	0.018102769	down	LRP3	LRP3
TRIB1	249.9103831	482.1740221	-0.948143082	0.018112967	down	TRIB1	
EEF1E1	1424.038374	535.1303355	1.412025804	0.01815897	up	EEF1E1	EEF1E1
GCC2	745.155336	389.1850921	0.937084755	0.018266004	up	GCC2	GCC2
PSPH	97.85325573	30.52452749	1.680650889	0.018284689	up	PSPH	PSPH
LOC107055669	119.5424129	47.06480611	1.344802011	0.018305594	up	LOC107055669	
KCNB1	48.76135346	98.38210086	-1.012657692	0.018388747	down	KCNB1	KCNB1
DR1	466.6421432	238.9825684	0.965411215	0.018590187	up	DR1	DR1
LOC107057238	4.365821499	21.74636694	-2.316449346	0.018680513	down	LOC107057238	
KLHL42	185.2438195	92.97457455	0.994517263	0.018701198	up	KLHL42	KLHL42
YPEL5	498.0570851	178.9908991	1.476424873	0.018813832	up	YPEL5	xp_005264423
TSC22D2	1379.66015	863.0675124	0.676767612	0.018945358	up	TSC22D2	TSC22D2
GTF2E1	359.4531748	577.0383529	-0.682863365	0.019229317	down	GTF2E1	xp_005247458
STK3	313.4298795	167.4441016	0.904463163	0.01928085	up	STK3	STK3
ACP5	5.116127086	67.24001999	-3.716196147	0.019284533	down	ACP5	
LOC101748428	55.46755752	196.7338372	-1.826529011	0.019312225	down	LOC101748428	
CTDSPL	1586.838191	931.2479469	0.76891778	0.019398638	up	CTDSPL	CTDSPL
ARL6IP5	2214.036748	1134.535587	0.964577305	0.019743566	up	ARL6IP5	ARL6IP5
RHOBTB1	302.459735	115.9203379	1.383609391	0.019866742	up	RHOBTB1	RHOBTB1

FBRSL1	338.5403274	218.5134746	0.631605454	0.019893721	up	FBRSL1	
CCL5	151.9652644	64.76480994	1.230459555	0.02013651	up	CCL5	CCL5
SMIM14	178.3975131	71.05817346	1.328022994	0.020149297	up	C4ORF34	SMIM14
BNIP2	850.5449551	308.8557421	1.461454338	0.02014958	up	BNIP2	BNIP2
SPATA7	110.0571108	68.73367063	0.67916345	0.020324079	up	SPATA7	SPATA7
TMEM135	511.5096327	196.335241	1.381442258	0.020649168	up	TMEM135	TMEM135
FCN2	1.655608772	10.79565365	-2.705016896	0.020655714	down	FCN2	FCN1
TAF1	867.5004519	1319.275279	-0.604809214	0.020886595	down	TAF1	
LOC107052100	40.4933815	70.9461749	-0.809038779	0.02088675	down	LOC107052100	
PRDM16	2.92650366	17.32661022	-2.565739445	0.021273006	down	PRDM16	PRDM16
LOC107049302	1.882408342	48.59904515	-4.690276445	0.021282755	down	LOC107049302	
MIR181B2	0.305756727	18.71468496	-5.935642713	0.021290211	down	MIR181B2	
UBE2J1	832.6549361	499.6252595	0.736872329	0.021503884	up	UBE2J1	UBE2J1
GTF3C3	97.73039586	160.4620413	-0.715352815	0.021514923	down	GTF3C3	GTF3C3
LOC107051384	1.792942547	14.72794378	-3.038154861	0.021554423	down	LOC107051384	
C6ORF57	286.4033559	131.3097104	1.125074789	0.021945504	up	C6ORF57	SDHAF4
FAM154B	11.59831168	2.043640952	2.504701157	0.02196399	up	FAM154B	
COG4	370.5947956	851.9997409	-1.201010371	0.022152902	down	COG4	COG4
LOC107056212	10.70643385	1.140822665	3.230331565	0.022359543	up	LOC107056212	
PLCB2	24.4173069	55.74318962	-1.190891468	0.022545167	down	PLCB2	PLCB2
PDZD2	33.97105745	62.74083939	-0.885098704	0.022614097	down	PDZD2	PDZD2
LOC107050556	5.324689494	78.58076868	-3.883406977	0.022627233	down	LOC107050556	
TUSC3	60.56077223	30.87915427	0.971750361	0.022649992	up	TUSC3	TUSC3
STAB1	95.39526185	377.8781876	-1.985931727	0.022708125	down	STAB1	STAB1
TNFRSF11B	837.4273468	513.8313674	0.704669067	0.022856734	up	TNFRSF11B	TNFRSF11B
LOC101747844	19.71844123	5.257294101	1.90715316	0.022860152	up	LOC101747844	xp_005258420
ZAK	1453.501823	935.9872438	0.634972107	0.022923276	up	ZAK	ZAK
LOC107054540	44.57460015	109.3188617	-1.294248581	0.022956383	down	LOC107054540	
LOC107051099	1.883866699	10.82712118	-2.522880906	0.022976936	down	LOC107051099	
ANKS3	261.9300196	694.8504037	-1.407520994	0.023177893	down	ANKS3	xp_005255173
LOC107056014	2471.022411	1138.711315	1.117706053	0.023411173	up	LOC107056014	
PROSER2	435.5752658	673.8241922	-0.629450193	0.02354233	down	C1H10ORF47	PROSER2
CARS	1155.461018	509.3062492	1.181863264	0.023579624	up	CARS	CARS
BNIP3L	1735.12113	841.0014673	1.044856159	0.023682621	up	BNIP3L	BNIP3L
LOC101750271	19.0470928	4.02206886	2.243561126	0.023696395	up	LOC101750271	
ZNF341	37.53433031	144.520626	-1.944992761	0.02387379	down	ZNF341	xp_005260641
MMP24	65.38866981	36.02659209	0.859978488	0.024049707	up	MMP24	MMP24
MRAP	5.28309168	17.75037807	-1.748395402	0.024221166	down	MRAP	MRAP
MS4A15	3.244227382	30.74328586	-3.244324525	0.024262552	down	MS4A15	MS4A8
IGSF9B	108.2948092	56.66678129	0.934388929	0.024350717	up	IGSF9B	IGSF9B
BCAR3	108.4848343	63.07248266	0.782410747	0.024802108	up	BCAR3	BCAR3
BNIP1	136.0493108	81.89461019	0.732289238	0.025191783	up	BNIP1	BNIP1
QRICH1	1235.46384	1953.280386	-0.660846272	0.025242178	down	QRICH1	xp_005265306
INPP4A	444.2571072	256.1587428	0.794356724	0.025375548	up	INPP4A	INPP4A
CNR1	29.19469298	63.57526723	-1.122759482	0.025428565	down	CNR1	CNR1
GK	446.0224891	211.1255467	1.079015296	0.02545733	up	GK	GK
TMLHE	261.4765571	396.6893407	-0.601328026	0.025573702	down	TMLHE	TMLHE
TUB	639.1859429	391.4449795	0.70742614	0.025717958	up	TUB	
RAB32	485.6080836	215.3991652	1.172779779	0.025828802	up	RAB32	RAB32
RASSF10	29.46732884	65.15311439	-1.144717854	0.025942081	down	RASSF10	RASSF10
CPEB4	216.4393075	114.1429367	0.923120944	0.026346129	up	CPEB4	CPEB4
PKIA	54.87486611	14.29872054	1.940259455	0.026473746	up	PKIA	PKIA
NCKAP1	3843.082458	2346.424133	0.711800117	0.026780217	up	NCKAP1	NCKAP1
GOLIM4	431.6277543	656.4538929	-0.60490605	0.026948093	down	GOLIM4	

CEP57	74.6549008	137.2272755	-0.878258387	0.027073456	down	CEP57	CEP57
LOC107051357	5.75157453	63.34458492	-3.461192432	0.027149255	down	LOC107051357	
DCP1A	469.2356027	213.0081559	1.13940381	0.027237885	up	DCP1A	DCP1A
ARPP19	2075.132768	942.7519792	1.138253465	0.027274989	up	ARPP19	ARPP19
ARHGAP29	604.2073626	373.4649584	0.694070881	0.027396881	up	ARHGAP29	xp_005271406
MEIS1	402.299916	651.1794951	-0.694783835	0.027419634	down	MEIS1	MEIS1
LOC107055609	50.01414779	19.02099885	1.394743247	0.027624497	up	LOC107055609	
LOC107055629	0.317723722	20.81981452	-6.034040596	0.02781777	down	LOC107055629	
LOC107050675	2.813103875	15.73206649	-2.483473458	0.027850334	down	LOC107050675	
ATMIN	350.8997266	158.9650138	1.142349542	0.028034621	up	ATMIN	ATMIN
BPHL	31.01872471	12.10457595	1.357586834	0.028060693	up	BPHL	BPHL
SLC23A2	453.5417505	217.289821	1.061614771	0.028083263	up	SLC23A2	xp_005260959
LOC107050831	38.87103021	364.9264364	-3.230838419	0.028160305	down	LOC107050831	
C12H3ORF18	91.47558417	170.8133704	-0.900962277	0.028189176	down	C12H3ORF18	
LOC101751203	52.72992074	164.8720163	-1.644652818	0.028363018	down	LOC101751203	MMP11
LOC107055276	3.424617328	14.72331994	-2.104088326	0.028396677	down	LOC107055276	
SKIDA1	3.02793645	14.41313281	-2.25097712	0.028448542	down	C2H10ORF140	
PHLDA3	6.373596621	81.75440243	-3.681116801	0.028501838	down	PHLDA3	PHLDA3
LOC101751717	0.953171166	9.868201824	-3.371980007	0.028762489	down	LOC101751717	
LOC107056791	31.00035094	77.35525724	-1.319214796	0.028775133	down	LOC107056791	
LOC101751061	59.88972394	166.5763485	-1.475803186	0.028832249	down	LOC101751061	
PLXDC2	478.568239	285.9845752	0.742787317	0.028902417	up	PLXDC2	PLXDC2
CD34	35.39345989	88.92476261	-1.329102419	0.028935288	down	CD34	CD34
NAA50	1360.326794	622.6526279	1.127453848	0.029004163	up	NAA50	NAA50
VPS8	1012.008922	1601.501082	-0.662202764	0.029190172	down	VPS8	xp_005247309
FBXO15	11.87152088	2.250177805	2.399393862	0.029209404	up	FBXO15	FBXO15
ZFAND1	248.8602755	86.41096655	1.526049634	0.029271059	up	ZFAND1	ZFAND1
INO80D	99.35111372	205.6331934	-1.049465118	0.029432879	down	INO80D	
LOC107050865	12.88890209	95.39259362	-2.887747882	0.029438147	down	LOC107050865	
PRDM5	85.48948155	45.5753591	0.907492901	0.02952561	up	PRDM5	PRDM5
GMIP	21.73818334	81.34359365	-1.903797349	0.029564211	down	GMIP	GMIP
LOC100858581	9.133135687	25.55271911	-1.484294647	0.029769271	down	LOC100858581	
LOC107050830	5.38452447	121.2806345	-4.493386457	0.029775015	down	LOC107050830	
RCAN1	855.5609442	295.8445555	1.532031281	0.029977357	up	RCAN1	RCAN1
DYRK3	875.7913208	550.2479902	0.670505182	0.030019039	up	DYRK3	DYRK2
LOC101750055	18.44608798	75.87741684	-2.040355681	0.03007152	down	LOC101750055	
FRMPD4	109.8799377	70.18233456	0.646748154	0.030468675	up	FRMPD4	FRMPD4
TMC3	314.4818823	481.0441691	-0.613192464	0.030587293	down	TMC3	TMC3
NUB1	659.1654205	312.6385595	1.076144836	0.031021354	up	NUB1	NUB1
LOC107050873	15.58846958	75.32529732	-2.272655166	0.031037028	down	LOC107050873	
IBA57	551.1431414	865.111917	-0.650459722	0.031186626	down	IBA57	IBA57
STEAP2	476.1351853	174.1695813	1.450878566	0.031266548	up	STEAP2	STEAP2
FOXO3	797.3387172	494.3801447	0.689571922	0.031422248	up	FOXO3	FOXO3
RP3-468K18.7	676.9910813	293.2505766	1.207002883	0.031550142	up	RP3-468K18.7	
FBXL21	143.0415761	55.18408733	1.374110316	0.032212543	up	FBXL21	xp_005266392
C5H14ORF169	112.9107909	391.6860434	-1.794514351	0.032257912	down	C5H14ORF169	C14orf169
CXADR	1916.370064	729.4461547	1.39350279	0.032282153	up	CXADR	CXADR
HS3ST2	0.635447444	8.601509057	-3.758745076	0.032376398	down	HS3ST2	
FAM168B	936.9698074	395.7465661	1.243425727	0.032399466	up	FAM168B	FAM168B
CXXC4	16.85031419	69.26351553	-2.039320123	0.032461527	down	CXXC4	
FRS3	258.4172583	575.312442	-1.154643248	0.03254552	down	FRS3	FRS3
LRPPRC	1922.056863	986.5740147	0.962151824	0.032581141	up	LRPPRC	LRPPRC
CLK4	756.1644964	360.4173657	1.069031588	0.032615304	up	CLK4	CLK4
PLEKHA3	849.0101687	362.4918504	1.227833273	0.032882617	up	PLEKHA3	PLEKHA3

LOC107052454	11.07056719	56.75434988	-2.358001831	0.032956687	down	LOC107052454	
LEMD2	821.2011595	1449.53689	-0.81978448	0.033147377	down	LEMD2	LEMD2
ERGIC1	3354.27667	1733.298385	0.952481658	0.033260502	up	ERGIC1	
PACSIN2	2842.293941	1341.408764	1.083306828	0.033311239	up	PACSIN2	PACSIN2
ATG2B	650.4843313	325.6365197	0.998251801	0.033380261	up	ATG2B	
CAMTA1	438.7697186	766.902417	-0.805579054	0.033642399	down	CAMTA1	CAMTA1
CD1B	2.493921795	13.34449289	-2.419756352	0.033698579	down	CD1B	CD1B
VILL	123.4261896	324.5363622	-1.394731582	0.033721826	down	VILL	VIL1
LOC769755	68.77212606	136.4440535	-0.988413668	0.033754349	down	LOC769755	NIPA2
RBMS3	556.5906984	213.3614041	1.383317575	0.034122069	up	RBMS3	RBMS3
RP11-463D19.2	228.0641698	90.57884734	1.332193723	0.03473727	up	RP11-463D19.2	
GOLGB1	1004.201202	2779.452653	-1.468752449	0.034824325	down	GOLGB1	
FAM185A	277.0187652	129.4581241	1.097498204	0.034969998	up	FAM185A	
CHCHD5	379.009748	677.1230508	-0.837183078	0.035019048	down	CHCHD5	CHCHD5
LOC107052014	7.700973033	34.62015768	-2.168499646	0.035378992	down	LOC107052014	
UBE2E1	673.2426337	394.5275369	0.77100054	0.035527506	up	UBE2E1	UBE2E1
ZBTB7A	89.13418	310.5816422	-1.800921889	0.035687741	down	ZBTB7A	ZBTB7A
RUNX2	203.0633737	131.1918755	0.630251667	0.035808145	up	RUNX2	RUNX2
LOC101750701	131.5095331	218.3669407	-0.731587074	0.035808796	down	LOC101750701	
XKR8	8.891452407	64.27341534	-2.85373113	0.035822468	down	XKR8	XKR8
WDR91	246.2189634	596.6900219	-1.277039772	0.036531283	down	WDR91	WDR91
PIP5K1B	122.6129688	59.59998389	1.040727735	0.03674314	up	PIP5K1B	PIP5K1B
SENP8	142.6021392	352.5315	-1.30575655	0.036791588	down	SENP8	SENP8
DISC1	186.5970635	114.6712542	0.702422501	0.036838122	up	DISC1	DISC1
GMFB	953.4197986	363.6346248	1.390622007	0.036941811	up	GMFB	GMFB
LOC428971	14.0611443	67.55228153	-2.26429049	0.036945703	down	LOC428971	SLC2A9
LOC107053887	13.18269182	2.19642259	2.58541743	0.037048794	up	LOC107053887	
LCT	5.815647977	27.09495407	-2.220012352	0.03708366	down	LCT	LCT
KLF5	582.360497	1320.481342	-1.181079514	0.03714076	down	KLF5	KLF5
SCAI	283.876902	145.9699769	0.959593801	0.037142544	up	SCAI	
HIST1H2B5	52.27367809	22.13487496	1.23976345	0.037186296	up	HIST1H2B5	HIST2H2BE
LOC107051444	3.435125966	38.51636951	-3.487036815	0.037503544	down	LOC107051444	
LOC107053327	271.1108867	598.8023659	-1.143196875	0.037677865	down	LOC107053327	
EHMT1	464.6828248	1105.915094	-1.2509224	0.037847162	down	EHMT1	EHMT1
CCNE2	14.75161491	35.32568681	-1.25984471	0.038114726	down	CCNE2	xp_005251152
HHIPL1	312.5847268	479.3966339	-0.61697249	0.038153446	down	HHIPL1	HHIPL1
SLC7A5	858.4329345	553.4483497	0.633256743	0.038216467	up	SLC7A5	SLC7A5
TMCO4	27.52274214	70.23085103	-1.351480702	0.038228564	down	TMCO4	xp_005245883
C16ORF45	278.0582122	180.6289789	0.622357579	0.038331816	up	C16ORF45	C16orf45
GJD3	30.21763442	84.03368884	-1.475577092	0.038435966	down	GJD3	
LOC107056560	135.0889845	57.51695152	1.23185092	0.038632559	up	LOC107056560	
LOC107054476	4.083998763	26.7519887	-2.711591809	0.03878997	down	LOC107054476	
NCOA2	380.879768	760.0574435	-0.996772803	0.039057037	down	NCOA2	
LOC107049278	153.8239696	8.353629013	4.202733444	0.039113046	up	LOC107049278	
DESI1	597.0942923	381.4107655	0.646613213	0.039966126	up	DESI1	DESI1
DDHD2	918.5039335	456.0752757	1.010013937	0.040830039	up	DDHD2	
LOC100857714	3.400683338	15.27834542	-2.167591737	0.040831845	down	LOC100857714	LY9
LOC107049738	54.18280054	162.5520695	-1.584995055	0.04089471	down	LOC107049738	
RHOT1	755.1084879	389.3381868	0.955660081	0.041002034	up	RHOT1	RHOT1
CHST13	43.38968101	97.20532254	-1.163683332	0.041070498	down	CHST13	CHST11
FHL1	495.4939818	314.4272959	0.656141069	0.041084161	up	FHL1	FHL1
DEGS2	39.7623347	17.69890085	1.167742702	0.041088753	up	DEGS2	DEGS2
MRM1	61.43688133	110.6149606	-0.848369634	0.041359162	down	MRM1	
PDLIM3	856.3320362	567.6397152	0.593194767	0.041860016	up	PDLIM3	PDLIM3

LOC101748371	18.55670765	36.70520968	-0.984044075	0.042266106	down	LOC101748371	
LOC107051218	1.757041562	11.39525452	-2.697212925	0.042481628	down	LOC107051218	
HCLS1	0.623480449	8.213001035	-3.719493254	0.042657893	down	HCLS1	HCLS1
TET1	198.8934108	113.3737897	0.810908331	0.042738879	up	TET1	TET1
CRYBG3	529.7622479	337.3438959	0.65112498	0.042893931	up	CRYBG3	CRYBG3
FAM53A	455.0041019	263.5824474	0.787625251	0.042912469	up	FAM53A	FAM53A
LOC101749146	97.42053726	45.95869623	1.083888068	0.043122112	up	LOC101749146	
MKNK2	754.9217701	323.2053819	1.223875929	0.04330497	up	MKNK2	
LOC107057132	10.69490359	131.7881721	-3.623225513	0.043563519	down	LOC107057132	
MIR6602	100.194987	44.18051823	1.181328082	0.043862912	up	MIR6602	
LOC107056628	299.1310441	161.5704292	0.888614464	0.043920348	up	LOC107056628	
TTPAL	167.8707477	100.3072527	0.742924932	0.043996924	up	TTPAL	TTPAL
PPM1A	2156.141184	1115.958404	0.950168395	0.044324803	up	PPM1A	PPM1A
XBP1	1074.2872	2128.880907	-0.98671551	0.044359332	down	XBP1	XBP1
ABCD3	1078.85349	429.6064288	1.32841147	0.044542405	up	ABCD3	ABCD3
TMEM30C	71.77626512	40.37644406	0.829992996	0.044647599	up	TMEM30C	
ATRIP	252.5484822	383.248056	-0.601718102	0.04471758	down	ATRIP	ATRIP
WASF3	123.058091	54.96058246	1.162870318	0.044829116	up	WASF3	WASF3
MYO6	726.2335119	460.2436048	0.658035829	0.044895464	up	MYO6	MYO6
CXXC5	270.3831779	155.494721	0.798139794	0.045046072	up	CXXC5	
KIF1BP	518.0152203	344.4229458	0.588813228	0.045194248	up	KIF1BP	
CEP85L	149.1977236	83.78684809	0.832429815	0.045241394	up	CEP85L	CEP85L
LOC101751422	813.7849529	3269.660724	-2.006421433	0.045263238	down	LOC101751422	
IRX2	19.81126047	83.78264447	-2.080330748	0.045717886	down	IRX2	IRX2
LOC107051872	40.97528971	90.16289382	-1.137779671	0.046020614	down	LOC107051872	
LOC107054407	2.72363808	11.80591451	-2.115902886	0.04686264	down	LOC107054407	
CHRNA7	32.2121208	13.00511624	1.308524355	0.046996636	up	CHRNA7	CHRNA7
CHL1	120.7473392	76.58342516	0.656887308	0.047257291	up	CHL1	CHL1
NFAM1	10.70643385	24.72125202	-1.207273791	0.047321379	down	NFAM1	NFAM1
LOC425623	48.93123477	327.7058602	-2.743573878	0.04747306	down	LOC425623	
LOC107051513	0.522047659	16.3908101	-4.97256183	0.047927054	down	LOC107051513	
ZNHIT6	310.8548053	124.2485827	1.323011485	0.048026003	up	ZNHIT6	ZNHIT6
RP5-1021I20.4	154.6867342	82.1339253	0.913299324	0.048084854	up	RP5-1021I20.4	
SMNDC1	1019.522954	527.2056845	0.951456426	0.048438312	up	SMNDC1	SMNDC1
LOC107056098	1.780975552	24.79032522	-3.799037581	0.048484328	down	LOC107056098	
DNAH12	64.97870018	25.94716171	1.32439015	0.048547096	up	DNAH12	
ACCS	76.8166519	138.1299581	-0.846535261	0.048632755	down	ACCS	ACCSL
LOC107050600	11.40930819	155.1613009	-3.765485556	0.048692472	down	LOC107050600	
KDSR	367.8829994	180.1693953	1.029893041	0.048816269	up	KDSR	KDSR
CBLL1	226.8291913	502.7667299	-1.148282868	0.048852311	down	CBLL1	CBLL1
RALGAPA1	931.4671252	582.5694666	0.677074763	0.048862491	up	RALGAPA1	RALGAPA1
PRAM1	1.985299489	10.8585887	-2.451408043	0.04911812	down	PRAM1	PRAM1
UNKL	283.8643347	151.3458148	0.907352817	0.049336437	up	UNKL	UNKL
C5H15ORF52	8.845042783	31.56018558	-1.835164662	0.049464294	down	C5H15ORF52	C15orf52
INAFM2	59.16203059	121.3808646	-1.036797527	0.049839442	down	INAFM2	
